# Context-invariant socioemotional encoding by prefrontal ensembles

**DOI:** 10.1038/s41467-025-59575-8

**Published:** 2025-07-01

**Authors:** Nicholas A. Frost, Kevin C. Donohue, Damhyeon Kwak, Vikaas S. Sohal

**Affiliations:** 1 Department of Neurology, University of Utah, Salt Lake City, UT USA; 2 School of Medicine, University of California, San Francisco, CA USA; 3 Department of Psychiatry and Behavioral Sciences, University of California, San Francisco, CA USA; 4 Weill Institute for Neurosciences, University of California, San Francisco, CA USA; 5 Kavli Institute for Fundamental Neuroscience, University of California, San Francisco, CA USA

**Keywords:** Neural circuits, Social behaviour, Emotion

## Abstract

The prefrontal cortex plays a key role in social interactions, anxiety-related avoidance, and flexible context-dependent behaviors, raising the question: How do prefrontal neurons represent socioemotional information across different environments? Are contextual and socioemotional representations segregated or intermixed, and does this cause socioemotional encoding to remap or generalize across environments? To address this, we imaged neuronal activity in the ventromedial prefrontal cortex of mice engaged in social interactions or anxiety-related avoidance within different environments. Neuronal ensembles representing context and social interaction overlapped more than expected by chance while nevertheless remaining largely orthogonal. Anxiety-related representations similarly generalized across environments while remaining largely orthogonal to contextual information. This shows how the ventromedial prefrontal cortex multiplexes parallel information streams using overlapping ensembles rather than transmitting information through largely distinct populations, thereby achieving context-invariant encoding alongside the context-specific reorganization of population-level activity.

## Abstract (web summary)

How the ensemble encoding of social and anxiety-related behaviors interacts with encoding of context in the prefrontal cortex of mice is not fully understood. Here authors examine how prefrontal neurons encode socioemotional behaviors in different contexts and reveal that the prefrontal cortex encodes context-invariant representations of these behaviors in parallel with representations of context.

## Introduction

The medial prefrontal cortex (mPFC) plays critical roles in regulating social interactions^1–3^ and anxiety-related behaviors^4–7^. Both types of behaviors can occur in a variety of different locations. Some socioemotional behaviors generalize across many environments and situations, while others require specific adaptations. Information pertinent to social interactions or anxiety-related behaviors must therefore be represented in a reliable manner which is also flexible across different situational contexts.

Social interactions are encoded by single prefrontal neurons^8,9^, neuronal ensembles^10,11^, and changes in neuronal interactions^12–15^. Underscoring the critical role of the mPFC for social interaction in mice^16^ and humans^17^, disrupting prefrontal computations or ensemble activity alters social behaviors^2,11,16^. Similarly, the mPFC encodes information pertinent to anxiety-related states^4,5^, presumably reflecting the influence of anxiety-related input from sources including the ventral hippocampus^6,7,18,19^.

Both social interactions^8^ and anxiety-related behaviors^20^ trigger specific patterns of mPFC output. However, the mPFC also encodes other types of information including location^8,21,22^ and context^23^, which may influence how social interactions or anxiety-related behaviors are encoded when they occur at different locations or in different environments. Underscoring the challenge of understanding how contextual and socioemotional encoding interact in prefrontal cortex, recordings in the mPFC of rodents and PFC of marmosets have demonstrated that activity underlying both exploration^23^ and social signals (specifically vocalizations)^24^ remaps dynamically in response to changes in context, and that mouse mPFC neurons may only be active when social investigation occurs at specific locations^8^. Thus, the mPFC encodes information relevant to socioemotional behavior and contextual information *in parallel*, providing a mechanism by which the mPFC can regulate these behaviors flexibly and in a context-dependent manner.

We therefore sought to understand how the population level or ensemble encoding of social and anxiety-related behaviors interacts with encoding of context in the mPFC of mice exposed to distinct environments. In general, we anticipate four general possibilities: (1) socioemotional and contextual information are encoded by largely non-overlapping neuronal ensembles; (2) the same neurons encode socioemotional and contextual information but socioemotional representations are context-invariant; (3) the same neurons encode socioemotional and contextual information, but socioemotional encoding fails to generalize across contexts; (4) socioemotional encoding undergoes remapping or interacts nonlinearly with contextual information, such that different ensembles encode socioemotional information in different contexts.

To address this question, we utilized microendoscopic calcium recordings of prefrontal neurons as mice engaged in social interactions or anxiety-related behaviors across different environments. Consistent with previous reports, we find that overall population-level activity within the ventromedial PFC (vmPFC) is dynamically reorganized in a context-dependent manner. Nevertheless, the specific neuronal ensembles which strongly encode socioemotional information are context-invariant. Furthermore, representations of both social and anxiety-related information maintain a near-orthogonal orientation relative to representations of contextual information. This enables socioemotional encoding to generalize across contexts, even though the underlying socioemotional and context-encoding ensembles utilize highly overlapping populations of neurons.

## Results

### Encoding of social and contextual information within the mPFC

**Fig. 1:**
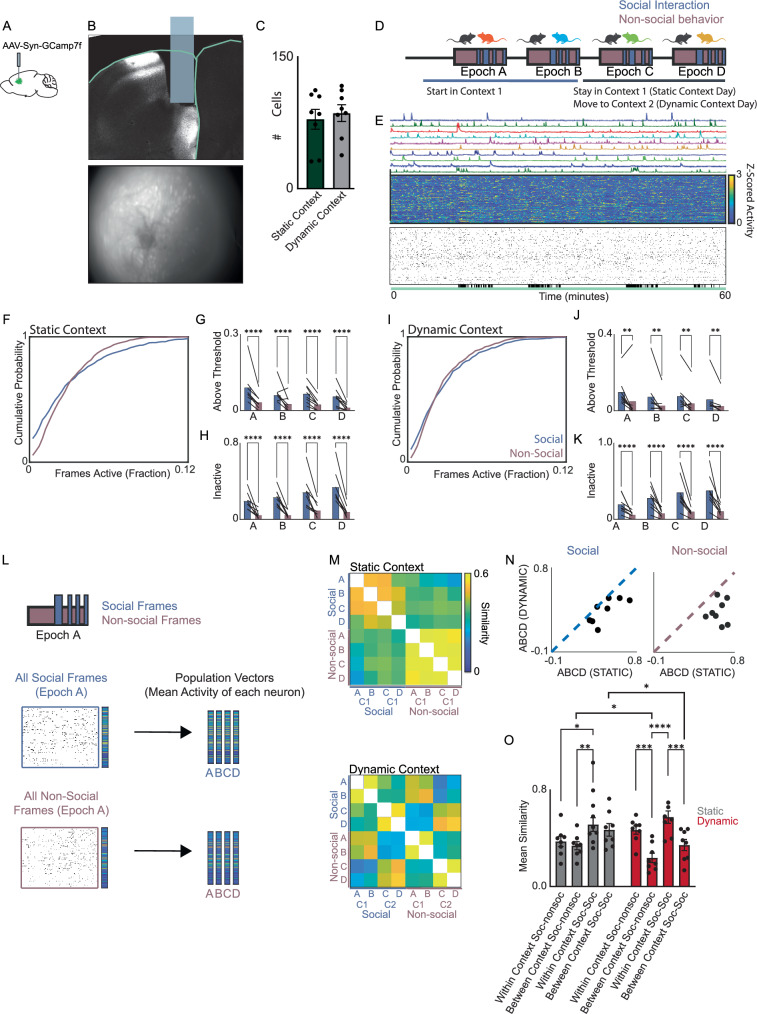
Reorganization of prefrontal network-level activity underlies dynamic contextual experience. **A** Schematic of surgeries and lens implantation. **B** Coronal section showing GRIN lens placement (top) and an example FOV during behavior (bottom). **C** The number of cells segmented for each mouse. Error bars represent SEM. **D** Schematic of experiment to image Ca^2+^ activity during social interaction occuring in different contexts. **E** Example of typical dF/F0 traces (top) Z-scored Ca^2+^ traces (middle) and binary raster of identified events (bottom) during static context imaging session. Black lines below the raster indicate social interaction bouts. **F** Cumulative probability function for proportion of frames that each neuron was active. **G** The proportion of highly active neurons (> 7.5% of frames) during social bouts and non-social periods. **** indicates *p* < 0.0005. **H** The proportion of inactive neurons during social interaction and non-social behavior. **** indicates *p* < 0.0005. **I** Cumulative distributions of activity as in F but for dynamic context. **J** The proportion of highly active neurons (> 7.5% of frames) during social interaction and non-social bouts. ** indicates *p* < 0.01. **K** The proportion of inactive neurons during social and non-social bouts by epoch. **** indicates *p* < 0.0005. **L** Schematic for calculating population activity vectors during social and non-social bouts within different epochs (A, B, C, or D). **M** Pairwise correlations between population activity vectors generated for social or non-social bouts. **N** Scatterplot of the average similarity between population activity vectors associated with social interaction (left) or non-social periods (right) within the static (*x*-axis) or dynamic (*y*-axis) context. Each dot is the mean of epochs A vs C, A vs D, B vs C, and B vs D). *p* < 0.01 for both. **O** The mean similarity (correlation) between social or non-social population activity vectors within the same context (i.e., epochs A & B or C & D) or between contexts. Error bars represent SEM. * indicates *p* < 0.05; ** indicates *p* < 0.01; *** indicates *p* < 0.001; **** indicates *p* < 0.0005. Source data are provided as a Source Data file.

We performed optical GCaMP measurements of multineuron patterns of prefrontal activity (Fig. 1a–e) in 8 mice. Each mouse was tested during sequential interactions with novel sex-matched juvenile conspecifics on two days. On the first day (‘static context’), all four interactions occurred within a single environment—the mouse’s home cage, and novel juveniles were introduced sequentially, interleaved with periods during which the mouse was alone (Figs. 1d and Supplementary Fig. 1). On a separate day (deemed ‘dynamic context’ because the social interactions occurred in two distinct locations), mice engaged in sequential social interactions within two distinct settings. Mice were first exposed to two novel juveniles in their home cage. They were then moved to a second context (which was familiar to them) where two additional novel juvenile conspecifics were sequentially introduced. The resulting dataset therefore contained periods of time during which the mouse was exposed to a novel juvenile or alone during 4 successive epochs (epochs ‘A’, ‘B’, ‘C’, and ‘D’). In the static context experiment all four epochs occurred in the home cage, whereas in the dynamic context experiment epochs A and B occurred in the home cage, while epochs C and D took place in a distinct environment / context.

We recorded activity from similar numbers of neurons in each case (Fig. 1c; mean 86 + /− 10 cells in the static context, 79 + /− 11 cells in the dynamic context, *n* = 8 mice). Individual neurons were identified using a combined PCA/ICA algorithm^25^, and events were detected as previously described^12^ to generate binary rasters (Fig. 1e) corresponding to periods when each neuron was active.

We calculated the mean activity of each neuron during social interaction or nonsocial periods. ‘Social interaction’ was defined as periods of active interaction with the novel juvenile (Supplementary Fig. 1), whereas ‘nonsocial’ included all timepoints the mouse was not engaged in interaction, regardless of whether the conspecific was present. We have previously observed that social interaction is encoded by both neurons whose activity is positively modulated during social interaction, as well as negatively-modulated neurons^12^. Notably, here we observed that the cumulative distribution of activity across neurons shifts between social interaction and the nonsocial condition, such that more cells had either very low or very high activity during social interaction in both the static (Fig. 1f; *p* < 0.01, two-sided KS test, *n* = 688 neurons from 8 mice, see also Supplementary Fig. 2) and dynamic context (Fig. 1i; *p* < 0.05, two-sided KS test, *n* = 632 neurons from 8 mice, see also Supplementary Fig. 2). We obtained similar results when we analyzed data by mouse—again, a significant difference in the number of neurons which were very active (> 7.5% of frames) during social interaction (Fig. 1g, j; static context: *p* = 0.0034; dynamic context: *p* = 0.0007, 2-way RM ANOVA with post-hoc Šidák correction, *n* = 8 mice). There was a similar difference in the number of silent neurons (Fig. 1h, k; static context: *p* = 0.0005; dynamic context: *p* = 0.0006, 2-way RM ANOVA with post-hoc Šidák correction, *n* = 8 mice).

This shift in activity could be driven by distinct sets of neurons in each epoch, or by a single socially-encoding ensemble whose members were consistently modulated by social interaction across epochs. To generate similarity matrices for each experimental day, we first obtained population activity vectors corresponding to the mean activity of each neuron during each social interaction or nonsocial epoch; then we calculated the similarities, i.e., pairwise correlation coefficients, between these population activity vectors (Fig. 1l–m). In the static context, population activity vectors for social interaction were very similar to each other, but distinct from those associated with nonsocial periods (Fig. 1m, top). By contrast, in the dynamic context, population activity vectors for social interaction and nonsocial activity were similar to each other if they were from the same context, but very different from both types of vectors from the other context (Fig. 1m, bottom).

To further quantify these changes in ensemble encoding we compared the similarity between social and nonsocial population activity vectors from epochs A and B with the same type of vectors (social or nonsocial) from epochs C and D. In both the social and nonsocial cases, these similarities were significantly decreased in the dynamic compared to the static context (Fig. 1n; social: static context mean correlation coefficient 0.47 + /− 0.05, dynamic context mean correlation coefficient 0.34 + /− 0.05, *p* < 0.01; non-social: static context mean correlation coefficient 0.60 + /− 0.03, dynamic context mean correlation coefficient 0.31 + /− 0.05, *p* < 0.05, two-sided signed-rank test, *n* = 8 mice). We obtain similar results when we computed the similarity for vectors from epochs B and C (Supplementary Fig. 3).

We next quantified the similarity between population activity vectors of different types, i.e., social vs. nonsocial (Fig. 1o). We computed this separately ‘within-context’ (i.e., between epochs A and B and also between C and D) and ‘between-context’ (i.e., between either A or B and either C or D). In the static context condition, the similarity between social and nonsocial activity vectors was not different within vs. between-context as the context does not actually change (*p* = 0.9). In the static context condition, the similarity between social activity vectors was also not different within vs. between-context (*p* = 0.8). However, in the dynamic context condition (in which epochs A and B have a different context than epochs C and D), social and nonsocial patterns of activity were significantly more similar within than between contexts (*p* = 0.0005). In the dynamic context condition, social activity vectors were also more similar within than between contexts (*p* = 0.0005; *p* < 0.01 for Experiment Day x Ensemble Similarity interaction by 2-way ANOVA with posthoc Tukey’s multiple comparison test, *n* = 8 mice). We found similar results when we restricted analysis only to neurons that were active in both contexts (Supplementary Fig. 4).

### Social interactions are robustly represented across different contexts

**Fig. 2:**
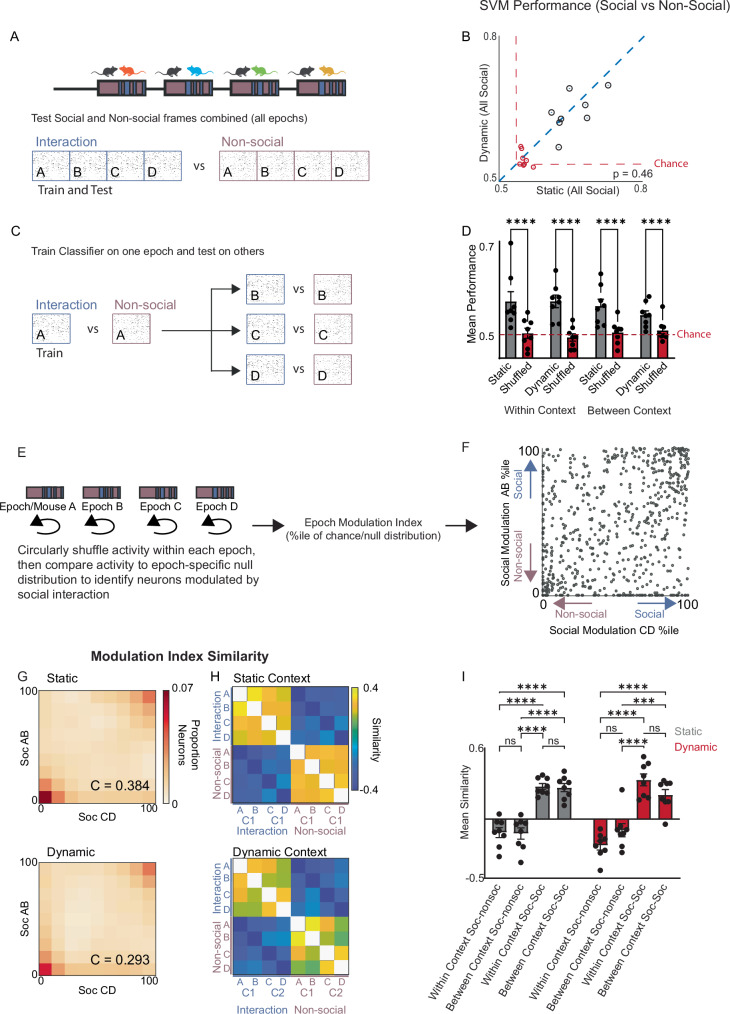
Context-invariant representations of social interactions. **A** Support Vector Machine (SVM) classifier was trained using binarized Ca^2+^ activity to discriminate frames corresponding to either social interaction or non-social periods across all epochs. **B** Performance in static (*x*-axis) vs. dynamic context (*y-*axis). **C** A linear classifier was trained to distinguish frames corresponding to social interactions vs. non-social periods for one epoch (A, B, C, or D), and then tested on the other epochs. **D** Classifier performance on real data (gray bars) was compared to the chance level based on randomly shuffled data which maintained the number of active cells in each frame and the overall number of events for each cell (red bars). Error bars represent SEM. **** indicates *p* < 0.0005. **E** Calculating the epoch-specific modulation index for each neuron by comparing its activity during social interactions to a null distribution generated by circularly shuffling its activity across both social and non-social frames within the epoch. **F** The social modulation index for individual neurons calculated using all social and non-social frames from epochs A&B (*y*-axis) or Epochs C&D (*x*-axis). **G** Heatmaps constructed from 10 × 10 matrices representing the proportion of neurons that fall into bins corresponding to deciles of social modulation indices. The social modulation index deciles are plotted for epochs A and B on the *y*-axis and for epochs C and D on the *x*-axis for the static (top) or dynamic context (bottom) experiments. **H** Similarity matrices constructed by calculating the correlations between vectors of modulation indices for different epochs (A–D) and behaviors (social interaction vs. nonsocial frames) within either the static context (top) or dynamic context (bottom) experiment. **I** The similarity between vectors of modulation indices for either social interaction (‘Soc)’ or non-social bouts (‘nonsoc’) within the same context (i.e., epochs A & B or C & D) or between contexts (i.e., epochs A & C or D or B & C or D) for either the static (gray bars, left) or dynamic (red bars, right) context experiment. Error bars represent SEM. *** indicates *p* < 0.001; **** indicates *p* < 0.0005. Source data are provided as a Source Data file.

Taken together, these data indicate that population activity during social interaction is strongly affected by context. In fact, in the dynamic context condition, social activity vectors were on average more similar to nonsocial activity vectors from the same context (mean correlation of 0.47) than to social activity vectors from the other context (mean correlation of 0.34). To examine whether the encoding of social interactions could be robust to changes in context, we trained a linear classifier to distinguish social from nonsocial timepoints based on the observed neural activity. Surprisingly, a linear classifier trained on all epochs, then tested on held out frames, distinguished social vs. nonsocial periods with similar accuracy regardless of the static vs. dynamic context condition (Fig. 2a-b; mean performance on real data: 62.3 + /− 1.7% in static vs 61.1 + /− 1.7% in dynamic, *p* = 0.46; mean performance on shuffled data 50.2 + /− 0.3% in static vs. 49.4 + /− 0.6% in dynamic, *n* = 8 mice, see also Supplementary Table 1). Note: the classifier performed with similar accuracy when trained and tested within individual epochs for both the static vs. dynamic context condition (Supplementary Fig. 5a-b, Supplementary Table 1).

Strikingly, the classifier performed significantly above chance levels even when trained on a single epoch then tested on an epoch from the other context, in both the static and dynamic context conditions (Fig. 2c-d; *p* < 0.005 for performance on real vs. shuffled data, *p* = 0.5 for within vs. between context, 2-way RM ANOVA with posthoc Tukey’s multiple comparisons test, *n* = 8 mice, see also Supplementary Table 1). This occurred despite a high false negative rate (Supplementary Table 1) which likely reflects the sparse nature of calcium activity. This indicates that despite the context-dependent reorganization of population-level activity patterns, specific patterns of activity persist which encode social interaction and are context-invariant.

We sought to determine whether the same neurons consistently encode social interaction across different contexts. For this, we calculated a ‘social modulation index’ (and ‘nonsocial modulation index’) within each epoch, (Fig. 2e), by expressing the observed activity level of each neuron as a percentile relative to a null distribution generated by circularly shuffling the data. In this way, neurons that increased or decreased activity during social interaction would have a high (e.g., > 90%ile) or low (e.g., < 10%ile) social modulation index, respectively. We first calculated a joint social modulation index for epochs A and B (‘AB’), and separately, for epochs C and D (‘CD’) (Fig. 2f). For both the static and dynamic context conditions, neurons that were strongly positively or negatively modulated by social interaction in AB tended to be similarly modulated in CD, as evidenced by a positive correlation between the vector of social modulation indices in AB and the corresponding vector in CD (Fig. 2g; static context: AB vs CD correlation coefficient = 0.384, p < 0.0001; dynamic context: AB vs CD correlation coefficient 0.293, *p* < 0.0001, two-tailed t-test, *n* = 688 neurons and 632 neurons from 8 mice, respectively). Notably, while we did not observe a strong relationship between modulation index and neuron activity (Supplementary Fig. 6), there was a strong correlation between the weight assigned by our linear SVM classifier (β) and modulation index (Supplementary Fig. 7).

To further explore this context-invariance of encoding, we generated similarity matrices by calculating the correlation between vectors of social or nonsocial modulation indices in each epoch (A, B, C, or D) for both the static (Fig. 2h, top) and dynamic (Fig. 2h, bottom) context conditions. This showed that social modulation index vectors from each epoch were similar to social modulation index vectors from other epochs, but distinct from nonsocial modulation index vectors (Fig. 2i). The similarity between social modulation index vectors was significantly greater than between social and nonsocial vectors for both within and between-context comparisons in both the static and dynamic-context conditions (*p* > 0.0001 for comparison (social-social and social-nonsocial comparisons within or between context), *p* = 0.09 for experiment day (static vs dynamic) *p* = 0.12 for interaction; 2-way RM ANOVA, posthoc testing performed using Tukey’s multiple comparisons test, *n* = 8 mice). We observed similar findings regardless of whether nonsocial interaction was defined as the mouse being alone vs. periods when a conspecific was present but the mice did not interact (Supplementary Fig. 8). Notably, in both cases we were able to discriminate social interaction from non-social behavior, whether alone or simply non-interacting (Supplementary Fig. 9). Taken together, these data confirm that despite context-driven changes in population-level activity patterns, the ensemble of neurons whose activity is strongly modulated by social interactions is largely consistent across contexts.

### Parallel encoding utilizes independent ensembles of overlapping neurons

**Fig. 3:**
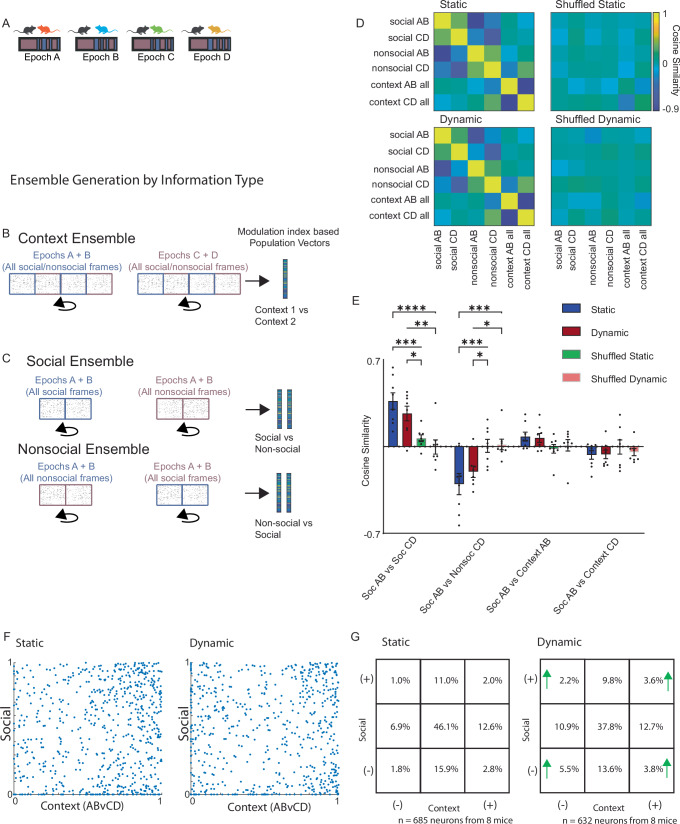
Representations of social information and context are heavily overlapping but near-orthogonal. **A** Schematic of dividing each dataset into sub-rasters corresponding to period of social interaction or non-social behavior (non-social rasters comprised frames that occurred between the social bouts plus the 6000 frames preceding introduction of the novel conspecific). **B** Schematic of generating vectors of modulation indices corresponding to the context-specific modulation of each neuron (i.e., the difference in their activity during epochs A and B vs. C and D, based on both social and non-frames, expressed as a %ile relative to the difference expected by chance based on shuffling each neuron’s activity 10,000 times). **C** Schematic of generating vectors of modulation indices corresponding to social behavior (top) by calculating the difference between each neuron’s activity during social (Soc) vs. nonsocial (Nonsoc) frames within a single context (i.e., either epochs AB or CD). This difference was expressed as a %ile relative to the difference expected by chance based on shuffled data. We generated vectors corresponding to nonsocial modulation indices via an analogous approach (bottom). **D** The similarity between pairs of social or context-specific ensembles quantified by calculating the correlation between vectors of the corresponding modulation indices. Real (left) and shuffled (right) datasets. **E** Similarities between social, nonsocial, and context-specific modulation index vectors on the static context (blue bars) or dynamic context (red bars) experiment days. Shuffled vectors were generated by randomly re-assigning the neuron associated with each modulation index value. The similarity between context and behavior-specific vectors do not differ from the chance level calculated using shuffled vectors. Error bars represent SEM. * indicates *p* < 0.05; ** indicates *p* < 0.01; *** indicates *p* < 0.001; **** indicates *p* < 0.0005. **F** Scatter plots of the social modulation index (calculated in context AB) vs. the context modulation index of each neuron in the static (left) or (dynamic) context experiment. **G** 9 × 9 table showing the proportion of neurons which were strongly positively (+) or negatively (-) modulated (i.e., modulation index > 90%ile or < 10%ile) by social interaction or context. Green arrows demarcate squares in which significantly greater overlap is observed in the dynamic context compared to static context. Source data are provided as a Source Data file.

Next, we sought to better understand whether this social ensemble might be non-overlapping and/or orthogonally oriented with respect to representations of contextual information. To test this, we calculated the modulation index vectors based on the degree to which neurons were modulated by context (A-B vs C-D) (Fig. 3a, b). Specifically, for each neuron, we computed the difference in activity observed during epochs A and B vs. during C and D as a percentile relative to the distribution of such differences calculated from circularly-shuffled data. We also calculated social (or nonsocial) modulation index vectors for each context (either epochs A and B or C and D) by calculating the activity of each neuron during social (or nonsocial) frames during epochs A and B (or C and D) as a percentile relative to the distribution observed in circularly-shuffled data (Fig. 3c). This resulted in a set of modulation vectors for each mouse which representing the modulation of each neuron by context, or by social interaction (within a context). Finally, we generated a similarity matrix for these modulation vectors (by computing the correlations between them). Social modulation vectors derived from epochs A and B (‘social AB’) were similar to those derived from epochs C and D (‘social CD’) (Fig. 3d, e; social AB-social CD correlation: static context real vs shuffled data, *p* < 0.005; dynamic context real vs shuffled data, *p* < 0.01). In contrast, on both days the similarity between the social modulation vectors and context modulation vectors was near 0 (indistinguishable from the similarity to shuffled data-derived vectors) indicating that the these representations were near-orthogonal (i.e., the angle between social and context modulation vectors was ~90 degrees) (social AB-context AB correlation: static context real vs shuffled data, *p* = 0.50; dynamic context real vs shuffled data, *p* = 0.83; social AB-context CD correlation: static context -real vs shuffled data, *p* = 0.80; dynamic context real vs shuffled data, *p* = 0.99; *p* < 0.0001 for comparison vector type (social vs. nonsocial vs. context), *p* = 0.56 for experiment day (static vs. dynamic context), *p* < 0.0001 for both comparison (real and shuffled soc-soc, soc-nonsoc, soc-context) and comparison-experiment day interaction, 2-way RM ANOVA with posthoc Tukey’s multiple comparison test, *n* = 8 mice). Notably, the similarity between vectors representing social interaction and those representing context also was not significantly different from 0 (social AB-social CD correlation vs. 0, *p* < 0.001 in the static context and *p* < 0.005 in the dynamic context; social AB-nonsocial CD correlation vs. 0, *p* < 0.01 in the static context and *p* < 0.005 in the dynamic context; social AB-context AB correlation vs. 0, *p* = 0.06 in the static context and *p* = 0.09 in the dynamic context; social AB-context CD correlation vs. 0, *p* = 0.09 in the static context and *p* = 0.11 in the dynamic context, uncorrected, two-sided one sample t-tests, *n* = 8 mice, Fig. 3e).

Social and context representations could be orthogonal because they are composed of non-overlapping neuronal ensembles. Alternatively, neurons which are strongly positively modulated by social interaction could be divided between positive and negative context-modulation such that context-driven changes in activity are roughly balanced (and the same for neurons which are strongly negatively modulated by social interaction). To compare overlap between social and context representations (the amount of overlap observed on the dynamic context day) to the level expected by chance / the passage of time (the amount of overlap on the static context day), we organized cells which were strongly positively (> 90%ile) modulated, non-modulated (10–90%ile) or negatively modulated (< 10%ile) by social interaction or context into 3 × 3 tables (Fig. 3f, g), corresponding to the static vs. dynamic context day, and determined whether any of the strongly modulated groups overlap significantly more or less on the dynamic day than on the static day. Surprisingly, we observed significantly more overlap on the dynamic day compared to the static day in every case (positive social-positive context: 3.6% in dynamic vs. 2.0% in static, *p* < 0.01; positive social-negative context: 2.2% in dynamic vs. 1.0% in static, *p* < 0.0001; negative social-positive context: 3.8% in dynamic vs. 2.8% in static, *p* < 0.05; negative social-negative context: 5.5% in dynamic vs. 1.8% in static, *p* < 0.0001; χ^2^ test; *n* = 632 cells in dynamic and 685 cells in static). Thus, of the neurons which were positively modulated by social interaction, a much larger fraction (38% vs. 21%) were also positively or negatively modulated by context in the dynamic context condition compared to the static context condition. Representations for different variables (social interaction vs. context) could achieve near-orthogonality by using non-overlapping neuronal ensembles, or alternatively by counterbalancing (within each ensemble) positive and negative modulations associated with the other variable. In this context, our data indicate that near-orthogonality is maintained via the latter strategy. Thus, while a substantial fraction of neurons are modulated by both context and social interaction, neurons which are positively modulated by one variable can be positively or negatively modulated by the other, permitting an approximately orthogonal relationship between representations of social interaction and those of context.

### Anxiety-related information is persistently represented in different contexts

**Fig. 4:**
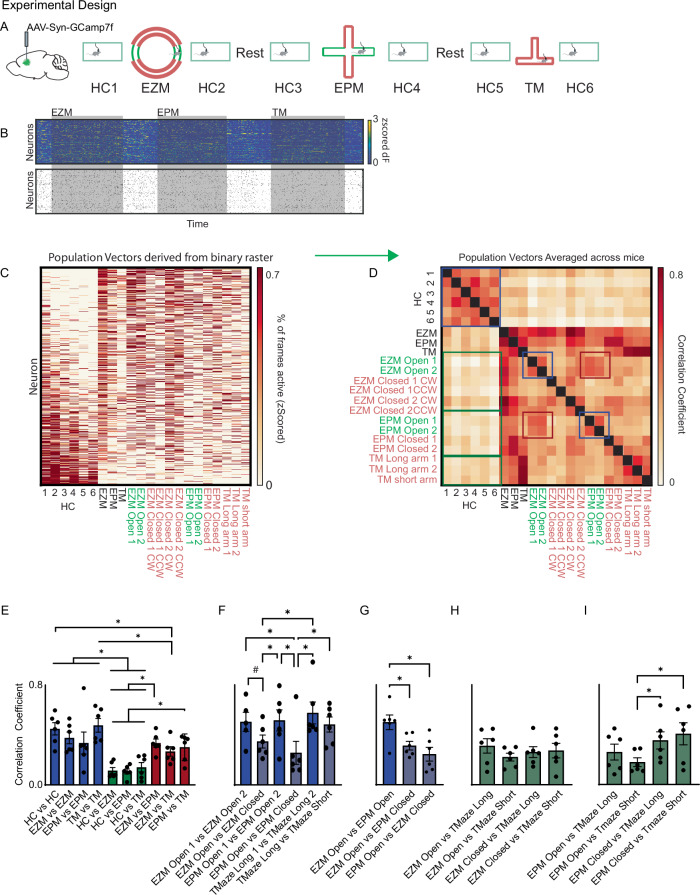
Context-invariant patterns of activity for anxiety-related information. **A** Experimental design: Ca^2+^ activity was recorded throughout a session in which mice explored the elevated zero maze (EZM), elevated plus maze (EPM), and a T-shaped maze). Mice were recorded within their home cage before and after exploring each of these novel mazes, and recording blocks were separated by 30 minutes of rest. **B** Example Z-scored Ca^2+^ activity (top) and binary activity raster (bottom). **C** Behavior was subdivided into each home cage epoch and the time mice spent within each arm of the EZM, EPM, and T-Maze. EZM closed arms were further divided by the direction of travel (clockwise, CW; counter-clockwise, CCW). We generated population activity vectors for each epoch and arm by calculating the mean activity of each neuron within each maze and each subregion (arm). **D** A similarity matrix by computing correlations between population activity vectors corresponding to different epochs / specific arms for each mouse, then averaging the resulting 6 similarity matrices. **E** The averaged similarities (correlations) between population activity vectors from various mazes. HC vs. HC = average similarity of population activity vectors from adjacent home cage epochs, e.g., HC1 vs. HC2. EZM vs. EZM = average similarity of all pairs of different population activity vectors from the EZM, e.g., EZM closed arm 1 CW vs. EZM open arm 2, etc. EPM vs. EPM and TM vs. TM were computed similarly. Data in E-I are subsets of mixed-effects analysis with matching across rows and between column, *n* = 6 mice except for EPM Open vs. EPM Open comparison as one mouse did not explore one of the arms. Error bars represent SEM. Asterisks represent discoveries. **F** The averaged similarities (correlations) between population activity vectors associated with specific arms within the same maze. *n* = 6 mice. Error bars represent SEM. Asterisks represent discoveries. #: q value = 0.059. **G**–**I** The averaged similarities (correlations) between population activity vectors associated with specific arms in different mazes. *n* = 6 mice. Error bars represent SEM. Asterisks represent discoveries. Source data are provided as a Source Data file.

We next tested whether these general principles (orthogonal representations for different types of information enabling context-invariance) extend to a distinct prefrontal-dependent behavior, namely anxiety-related avoidance. For this, we measured prefrontal neuronal activity in mice over epochs in which they explored an elevated zero maze (EZM), elevated plus maze (EPM), and a T-shaped maze (T-Maze) (Figs. 4a and Supplementary Fig. 10). The EZM and EPM both utilize open and closed arms positioned atop an elevated platform and are commonly used to assay anxiety-related avoidance in rodents. By contrast, the T-Maze consists of three closed arms. Each epoch was preceded and followed by a period of recording when the mouse was alone in its home cage and two 20-minute rest periods during which the camera was turned off.

We recorded 633 neurons from 6 mice. As expected, mice displayed a preference for the closed arms of the EZM and EPM, but no clear preference for specific arms in the T-Maze (Supplementary Fig. 10). As before, we converted the activity of each neuron to a binary raster corresponding to frames in which it was active Fig. 4b). Visual examination of pooled population activity vectors (Fig. 4c)., which showed activity across all mice during epochs in the home cage or specific mazes, illustrated gross differences in network activity. We then generated population activity vectors for specific types of exploration (home cage, EZM / EPM / Tmaze, EZM / EPM open / closed arms, etc.) and each mouse, calculated correlations between these vectors to generate similarity matrices (for each mouse), then averaged across mice to obtain a mean similarity matrix (Fig. 4d). This similarity matrix shows that distinct patterns of activity are associated with home cage exploration vs. exploration of the different mazes (**green boxes in** Fig. 4d and Supplementary Fig. 11). Specifically, the similarity between activity vectors associated with different arms within the same maze was significantly higher than the similarity between activity vectors for each maze and those associated with home cage exploration (**blue vs. green bars in** Fig. 4e; EZM vs. EZM mean correlation coefficient 0.38 + /− 0.05, EPM vs. EPM mean correlation coefficient 0.34 + /− 0.09, Tmaze vs. Tmaze mean correlation coefficient 0.48 + /− 0.05, HC vs. EZM mean correlation coefficient 0.12 + /− 0.02, HC vs. EPM mean correlation coefficient 0.11 + /− 0.02, HC vs. Tmaze mean correlation coefficient 0.14 + /− 0.02; *p* < 0.0001, mixed-effects analysis, post-hoc control of false discovery rate performed using two-stage method of Benjamini, Krieger, and Yekutieli, *n* = 6 mice). Furthermore, the activity vectors for the different mazes (EZM, EPM, Tmaze) were significantly more similar to each other than to those from the home cage (**red vs. green bars in** Fig. 4e).

As expected, there is high similarity between population activity vectors associated with each of the two open arms from the same maze (EZM or EPM) (**blue boxes in** Fig. 4d and Supplementary Fig. 11). However, we also observed very high similarity between population activity vectors from the EZM open arms and those from the EPM open arms (**red boxes in** Fig. 4d**and** Supplementary Fig. 11). Activity vectors for the EZM open arms were significantly more similar to those for the EPM open arms than to those for the EPM closed arms (and vice-versa) (Fig. 4g; EZM Open vs. EPM Open mean correlation coefficient 0.50 + /− 0.06, EZM Open vs. EPM Closed mean correlation coefficient 0.31 + /− 0.04, EPM Open vs. EZM Closed mean correlation coefficient 0.25 + /− 0.06; post-hoc control of false discovery rate performed using two-stage method of Benjamini, Krieger, and Yekutieli, *n* = 6 mice). Otherwise, activity vectors for different maze subregions were relatively dissimilar, except for the EPM closed arms and the TM long arms, perhaps reflecting geometric similarity (Fig. 4h–i). We observed similar results using population activity vectors based on Z-scored calcium traces instead of binary activity rasters (Supplementary Fig. 12).

**Fig. 5:**
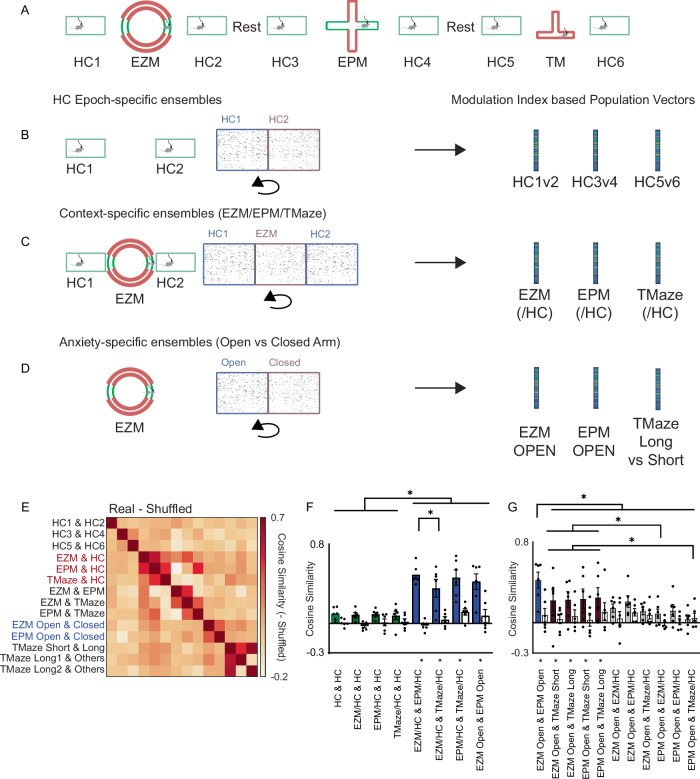
Anxiety-related information and context are encoded by largely orthogonal ensembles. **A**–**D** Context-specific modulation indices for the EZM, EPM, or T-maze were each generated by expressing the mean activity of each neuron within that maze as a %ile relative to a distribution of mean values expected based on shuffling across all frames from that maze and the adjacent HC epochs. Anxiety-specific ensembles were generated by expressing the mean activity of each neuron during open arm exploration as a %ile relative to a distribution of mean values derived after shuffling across all frames from that maze (EZM or EPM). We calculated modulation index vectors for the long or short arms of the T-maze analogously, by comparing mean activity in that arm to a distribution expected by chance derived by shuffling across all T-maze frames. **E** The similarity matrix showing the difference in similarity (correlation) between each pair of modulation index vectors (associated with context, anxiety-specific encoding, or specific arms of the T-maze) between real and shuffled data. **F** The average similarity (correlation) between pairs of modulation index vectors corresponding to differences between HC epochs (‘HC & HC’), differences between specific mazes and the adjacent HC epochs (EZM/HC, EPM/HC, or TMaze/HC), or anxiety-related information in the EPM vs. EZM (EZM open & EPM open). Panels F and G show subsets of the data from a single statistical analysis, and have been separated into two panels for clarity. Gray bars represent shuffled data. Shuffled data not shown but * below *x*-axis indicate similarities that are significantly different (*p* < 0.05) from those calculated from shuffled values. Error bars represent SEM. **G** The average similarity (correlation) between vectors of modulation indices associated with specific arms within a maze, i.e., the open arms of the EZM or EPM, or the long or short arms of the T-maze. Shuffled data not shown but * below *x*-axis indicate comparisons that differ significantly from shuffled data. Error bars represent SEM. Asterisks represent discoveries. Source data are provided as a Source Data file.

These findings suggest that prefrontal representations of anxiety-related information, like social information, persist across different contexts. To quantify the degree to which anxiety-related representations overlap with representations of context we again generated vectors of modulation indices, corresponding to the degree to which each neuron’s activity differed between contexts or sub-regions of a maze (Fig. 5a–d). Specifically, we calculated the difference between each neuron’s mean activity in two conditions (e.g., home cage vs. EZM, EZM open vs. closed arms, etc.) as percentiles relative to distributions based on shuffled data. We did this to identify ensembles of neurons which were differentially active between different home cage epochs (Fig. 5b), ensembles which distinguished each maze from the adjacent home cage epochs (Fig. 5c), and ensembles which discriminated different compartments within a maze, e.g., the open vs. closed arms (Fig. 5d). We then computed the similarity between different vectors of modulation indices to quantify the similarity between neuronal ensembles encoding specific contexts and/or anxiety-related information (Fig. 5e).

The modulation index vectors distinguishing each maze from adjacent home cage epochs were significantly more similar to each other than were vectors distinguishing different home cage epochs or shuffled data (**green vs. blue bars in** Fig. 5f; *p* < 0.005 for shuffled vs. real data, *p* < 0.0001 for both ensemble comparison and interaction, 2-way RM ANOVA, *n* = 6 mice). This indicates that largely overlapping ensembles encode the contexts associated with the EZM, EPM, and Tmaze. The similarity between modulation index vectors for the EZM open vs. closed arms and vectors for the EPM open vs. closed arms was also significantly higher than the similarity between modulation index vectors for different home cage epochs, or the similarity between vectors distinguishing each maze from home cage epochs (Fig. 5f; posthoc control of false discovery rate performed using two-stage method of Benjamini, Krieger, and Yekutiel, *n* = 6 mice). This confirms that neuronal ensembles encoding anxiety-related information persist across different contexts. We also observed similar results utilizing vectors generated from Z-scored calcium traces instead of binary activity rasters (Supplementary Fig. 13).

Finally, we trained support vector machines (SVMs) to distinguish frames corresponding to different contexts (e.g., EZM vs. adjacent home cage epochs), home cage epochs (e.g., home cage epoch 1 vs. 2), or subregions within the same maze (e.g., the open vs. closed arms of the EZM). We tested each of these SVMs on each of the 14 pairs of mazes (e.g., EZM vs. HC, EZM vs. EPM, etc.), epochs (HC1 vs. HC2, etc.), and subregions (e.g., EZM/EPM open vs. closed, TM short vs. long) (Supplementary Fig. 14a). If similar neuronal ensembles encode contexts corresponding to each maze, or anxiety-related information across two contexts (EZM and EPM), then the classifier should perform above chance in one context after having been trained on neural activity from the other. Indeed, the classifier performed well above chance when trained to distinguish one maze from adjacent home cage frames and then tested on a different maze (Supplementary Fig. 14b–c). The classifier also performed equally well at distinguishing open arm frames from closed arm frames, when trained on one maze (EZM or EPM) and then tested on the other maze (but not when tested on Tmaze arms) (Supplementary Fig. 14d); In contrast, SVMs performed at chance levels when they were trained to distinguish context (e.g., EZM vs. HC), and then tested on discrimination of open arms vs. closed arms of the EZM or EPM, again supporting the finding that ensemble representations for context are largely orthogonal to those for anxiety-related information (Supplementary Fig. 14d).

## Discussion

Successful behavior requires reliable representations of pertinent information across varying contexts and conditions. While ensemble representations and other forms of multineuron encoding may increase reliability^12,26–29^, information pertaining to multiple behavioral variables will be represented via overlapping ensembles, potentially changing and/or confounding the meaning of changes in the activity of individual neurons. Neuronal activity can also change with the passage of time and accumulation of behavioral experiences^30,31^, potentially further interfering with the stability of representations for particular types of information. Numerous studies have quantified the ability of single neurons and multineuron ensembles to encode social information^10–12,32–34^. Previous studies have found that social ensembles are approximately orthogonal to those associated with exploratory behavior in the amygdala and distinct from those associated with feeding behavior in orbitofrontal cortex^11,32^. Still, it remains unclear how ensembles encoding social information interact with the contemporaneous encoding of other behavioral variables, particularly context, in prefrontal cortex. This is a critical question as contextual features of an environment are known to alter neuronal activity underlying learned behaviors^23,35^, and contextual representations within the mPFC play a key role in episodic memory formation and spatial processing^36^.

**Fig. 6:**
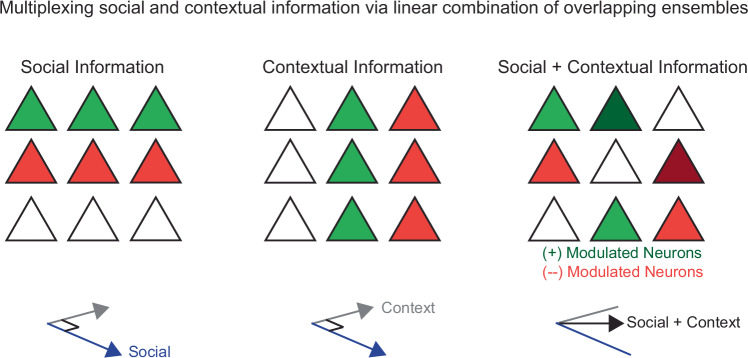
Multiplexing social and contextual information via linear combination of overlapping, orthogonal ensembles. Ensemble representations of social and contextual information are comprised of neurons which may be positively or negatively modulated. These ensembles overlap, however the response of a cell to one type of information does not predict its response to other types of information, resulting in vectors which are largely orthogonal from eachother. Because these vectors sum approximately linearly, persistent encoding of socioemotional behavior can be maintained despite context-driven changes in activity.

Here, we show that neuronal ensembles modulated by social behavior remain largely invariant during changes in the environment, allowing social encoding to generalize across contexts. Notably, we still found extensive context-dependent reorganization of population-level activity *during* social interaction (when we examined population activity vectors), and this context-invariance was only evident when we specifically examined the encoding of specific behavioral variables by individual neurons (using modulation indices) or the entire network (using SVM classifiers). Surprisingly, this context-invariance occurs despite greater than expected overlap between context and social encoding neurons, because these two representations are largely orthogonal (Fig. 6). Social ensembles were also largely orthogonal to ensembles associated with the passage of time in the static context experiment (i.e., ‘drift’)^31,37,38^. Similarly, anxiety-related ensembles, recruited during exploration of maze arms typically viewed as anxiogenic, persist across different maze geometries, enabling context-invariant encoding of anxiety-related information. This type of generalization fits with traditional views of the prefrontal cortex as an ‘executive controller’ that represents abstract features of the environment and behavioral states in order to facilitate decision-making^39^. Of course, it is unclear whether the context-invariance of these representations arises within the prefrontal cortex or upstream regions, or via interactions between them. Other connected regions such as the ventral hippocampus may contribute to contextual or social encoding – or both. However, the context invariance of social encoding suggests that information related to social or contextual information may arrive via behavior-specific inputs from one or more region, and the behavior-specific activity of individual neurons may be maintained by recurrent local or subcortical connections, e.g., persistent activity in corticalthalamic circuits^1^.

The ability of the prefrontal cortex to multiplex socioemotional and contextual representations using overlapping neurons also aligns with the view of prefrontal neurons as possessing mixed selectivity. Such mixed selectivity could facilitate the formation of multimodal representations that store flexible context-specific associations and/or guide context-specific behaviors. It is unknown whether specific mechanisms or circuit parameters are necessary to achieve this multiplexing and maintain discrete representations in near-orthogonal configurations. Furthermore, abnormalities in social and anxiety-related behaviors are common manifestations of many neurodevelopmental^1,9,12,16,19,40,41^ and neurodegenerative disorders^42–45^. As many studies have begun studying representations of social and anxiety-related information in mouse models of disease, it will be important to explore whether the context-dependence of these representations might be disturbed, leading, for example, to abnormal instability of social encoding or the inappropriate generalization of anxiety-related representations. Finally, while this study included both sexes, we were underpowered to detect sex-differences in socioemotional information processing. This remains an important future direction given the strong sex biases in many neuropsychiatric conditions.

## Methods

All experiments were conducted according to the National Institutes of Health (NIH) guidelines for animal research, and protocols were reviewed and approved by the Institutional Animal Care and Use Committee (IACUC) at the University of California, San Francisco (UCSF; protocol number AN185374).

### Stereotactic injection and lens implantation

C57/B6J mice were obtained from Jackson Laboratories (Bar Harbor, ME) and maintained in our animal facility with standard light-dark cycle, humidity between 30–70%, and temperature ranging 68–79 degrees F. To image neuronal activity during social interactions and anxiety-related behaviors we utilized adult male and female mice housed and bred in the UCSF animal facility. We used 5 female and 3 male mice in these experiments, but did not specifically examine sex-specific differences. To image prefrontal ensemble activity during freely moving behavior, mice were injected with AAV8.Syn.gCaMP7f.WPRE.SV40 (Penn Viral Core). Coordinates for injection into mPFC were (in mm relative to Bregma) [+1.7 (AP), –0.3 (ML), and –2.3 (DV)], corresponding to ventromedial PFC. 1 mm GRIN lenses with an integrated baseplate (Inscopix, Palo Alto) were implanted at the same AP and ML coordinates to a depth of 2.1 mm. These coordinates result in an imaging plane near the dorsal aspect of infralimbic cortex. Imaging experiments were performed by mounting a microendoscope (Inscopix nVoke) at least 4 weeks after lens implantation to permit recovery. All surgeries were performed under isoflurance anesthesia and post-operative analgesia was administered in accordance to protocols reviewed and approved by the UCSF IACUC.

### Social behavior

Adult mice were accustomed to the room and handling by observer for at least 3 days prior to experiments, with microendoscope attached. In addition, to mitigate handling or context-induced anxiety, each mouse was habituated to the second context which contained clean bedding for 1 h daily on 5 consecutive days prior to imaging experiments. Each mouse is habituated to a different cage so that there are no unfamiliar rodent odors. The home cage/context 1 measured 11.5 × 7.5 inches and the second context measured 10.5 × 14 inches. On test day, mice were habituated with the scope turned on for 5 minutes prior to starting recordings/acquiring images. During social epochs, novel sex-matched juvenile mice (< 6 weeks) were sequentially introduced to the cage containing the test mouse (either the test mouse’s home cage or the second context). In this manner each mouse engaged in social interaction with 4 novel juveniles, either within their own home cage or spit across the two contexts. Dynamic and static context recordings were obtained on different days and consisted of 10-minute baseline recordings preceding the first and third epoch followed by sequential epochs consisting of 5 minutes of social interaction and interleaved periods in which the mouse was alone. Social behavior was scored manually. Each social bout began when the test mouse moved towards the conspecific target, with its nose oriented towards the conspecific, and its nose either contacted or was immediately adjacent to the conspecific. Each social bout ended when the test mouse or the conspecific target moved apart such that the nose of the test mouse was no longer in contact with, or immediately adjacent to, the target.

### Anxiety-related behavior

Adult mice (3 male and 3 females) were habituated to the room and observer for at least 3 days prior to recording. These mice also completed the social behavior imaging experiments. Imaging experiments were performed over a single day with the microendoscope attached throughout the experiment. Imaging experiments consisted of three primary epochs defined by a primary maze which (EZM, EPM, and tMaze) separated by a rest period of 20 minutes during which the microendoscope was turned off. Each epoch consisted of 10-minute recordings in the home cage before and after 10 minutes in which the mouse explored the respective maze.

### Image acquisition and segmentation

Images were acquired using an nVoke microendoscope (Inscopix, Palo Alto) attached to a laptop computer and synced to a separate video acquisition computer running Anymaze. The frame rate was 20 Hz, and the light power was 0.2 mW. Acquisition was performed using 4 × 4 pixel binning. Throughout all figures ‘frames’ refers to a unit of time (50 msec) corresponding to the acquisition of a single image within a calcium movie. We segmented images as previously described using a modified PCA/ICA approach^12,25^. Specifically, we used the output from the PCA/ICA to identify a set of contiguous pixels representing a single neuron, then averaging the signal within those pixels. We then subtract the signal within the neuron from the average signal in the surrounding pixels to deconvolve the activity of each neuron from the surrounding neuropil, then low pass filtered the resulting traces to remove high-frequency noise using the MATLAB command: designfilt (“lowpassfir,” “PassbandFrequency,” 0.5, “StopbandFrequency,” .65, “PassbandRipple,” 1, “StopbandAttenuation,” 25). We identified calcium transients based on the amplitude and first derivative of deflections from baseline as previously described^12^, adjusting parameters corresponding to the first derivative of the dF/F_0_, the amplitude, and the integrated area under the curve of identified events to achieve > 95% sensitivity and specificity for each dataset.

### Quantifying ensemble activity

We then converted datasets to binary rasters denoting periods in which each neuron was active over the course of the experiment. We generated population vectors corresponding to the fraction of frames each neuron was active during the specified behavior/epoch. We performed this analysis first by pooling all neurons from each mouse to generate a single population vector for each epoch/behavior. Similarity matrices were generated by calculating the pairwise correlation coefficient between each set of population vectors. Analysis of individual mice was performed by generating population vectors corresponding to the fraction of frames each neuron was active within each behavioral epoch and generating a similarity matrix corresponding to the pairwise correlations between each behavior-specific population vector for each mouse. Population vectors based on dF/F_0_ were generated by calculating the mean Z-scored calcium activity for each neuron within each behavior/epoch from which corresponding similarity matrices were generated as above.

### Quantifying behaviorally modulated neurons

The activity of each neuron was quantified as the fraction of frames in which it was active during a particular behavioral epoch. To disambiguate changes in activity driven by social interaction from context-driven changes we calculated the mean activity for each neuron over all frames corresponding to a given behavioral variable in real or shuffled data within a given epoch. We generated a null distribution for each neuron within each epoch by circularly shuffling the data 10,000 times to calculate the activity that would be expected by chance during a given epoch-specific behavior. This permitted us to generate an epoch-specific modulation index for each neuron corresponding to its mean activity during a specified behavior as a percentile relative to the null distribution, with high percentiles (> 90^th^ percentile) denoting that the real activity of the neuron during a behavior was greater than expected by chance, and a low percentile (< 10^th^ percentile) denoting neurons which were less active during a specified behavior than would be expected by chance (compared to all activity during that epoch). Modulation index vectors contained the modulation index of each neuron within a given behavior/epoch and were used to generate similarity matrices. For the social/context experiment, an epoch was defined as beginning 5 minutes preceding the onset of social interaction with each mouse and ending when a given conspecific was removed from the home cage. For the anxiety/context experiment, an epoch was defined as the entire time that a mouse spent within a given maze.

### Linear classifier

We trained a Support Vector Machine to distinguish frames corresponding to social interaction from frames corresponding to non-social behavior. Models were trained using the command “fitclinear” in MATLAB. The classifier was first trained and tested on all social and non-social frames from the four epochs and performance was averaged over 500 iterations, leaving 25% hold out. In a separate analysis, we trained and tested the classifier on social and non-social frames within each epoch independently, in this case, leaving 20% hold out. As the number of frames in which mice engaged in social interaction varied and were not equal to the number of frames in which mice engaged in non-social behavior, we subdivided the data by behavior and randomly selected an equivalent number of frames corresponding to social interaction or non-social behavior on each iteration. Finally, to understand if signatures of neural activity identified in one epoch could inform the classifier across different epochs, we trained the classifier on 100% of frames from each epoch (prior to randomly downsampling on each iteration to balance the data), and then tested the classifier on data from the remaining three epochs. This was performed over 500 iterations. Shuffled data was generated by randomly swapping the identity of the neuron assigned to individual calcium events so that both the number of events in each frame and the number of events assigned to a given neuron were unchanged in the resulting surrogate dataset. We utilized an analogous approach to distinguish frames underlying different mazes, or locations within anxiety-provoking environments. Here we again trained the classifier to distinguish between a pair of contexts or locations based on calcium data, then utilized the trained model to distinguish between a different pair of contexts over 200 iterations. As with social data we utilized 100% of frames within each epoch and utilized balanced training data. F1, Precision, and Recall for each model is reported in Supplementary Table 1, where:

1 $${Precision}=\,\frac{{True\; Positives}}{{True\; Positives}+{False\; Positives}}$$

2 $${Recall}=\,\frac{{True\; Positives}}{{True\; Positives}+{False\; Negatives}}$$

3 $$F1=\frac{2*{Precision}*{Recall}}{{Precision}+{Recall}}$$

### Orthogonality of Ensembles

To compare social ensembles to context specific ensembles we first generated population vectors corresponding to the activity of each neuron during all frames in which mice were engaged in social interaction, and all of the frames in which mice were not engaged in social interaction (non-social frames). We then determined a modulation index for each neuron by comparing its activity during social frames to non-social frames. We removed neurons which were not active during either set of frames. We next generated population vectors corresponding to context by calculating the mean activity of each neuron in epochs A&B, and for epochs C&D. We next determined the degree to which each neuron was modulated in epochs A&B compared to C&D using all frames (social + non-social). We then zero-centered the modulation index of each neuron and compared the cosine similarity between resulting social- and context-specific vectors, where:

4 $$\cos \theta=\,\frac{{Vector}1\cdot {Vector}2}{\left|\left|{Vector}1\right|\right|\left|\left|{Vector}2\right|\right|}$$

Shuffled comparisons were generated by shuffling cell labels on modulation vectors. We used an analogous approach to compare context-specific and anxiety-specific population vectors as detailed in Fig. 5.

### Statistical analysis

Neurons were pooled into population vectors corresponding to entire datasets and activity of these pooled vectors was compared using KS-test and *t*-test as denoted in the manuscript. We then constructed pseudo-ensembles for each behavior corresponding to the activity (or modulation index) of all of the neurons during a specified behavioral epoch. We compared these population vectors by calculating the Pearson correlation coefficient between each pair of vectors. We performed analogous comparisons on population activity and vectors generated for each mouse during each behavior. The activity of population vectors generated for each mouse was compared using repeated measures ANOVA for epoch and behavior (social vs non-social behavior). All ANOVAs were performed using Graphpad Prism. All other statistical comparisons were performed using MATLAB. To compare the degree to which population vectors changed across contexts, we calculated the Pearson correlation coefficient between specified pairs of vectors and compared the result using Wilcoxon signed-rank test. Classifier performance is defined as the mean fraction of frames classified correctly over all iterations in which the classifier was trained and tested. Post-hoc comparisons of population vector correlations, cosine similarity, and classifier performance utilized the two-stage method of Benjamini, Krieger, and Yekutiel to control the rate of false discovery.

### Reporting summary

Further information on research design is available in the Nature Portfolio Reporting Summary linked to this article.

## Supplementary information

Supplementary Information

Reporting Summary

Transparent Peer Review file

## Source data

Source Data

## Data Availability

The data generated in this study have been deposited in the Zenodo database (10.5281/zenodo.10113326). Source data are provided with this paper.

## Code availability

Custom code used to perform analysis is available on author’s Github (nickfrostneuro/Static-Dynamic).
