# Supplementary material for: Context-invariant socioemotional encoding by prefrontal ensembles
Source: Nat Commun. 2025 Jul 1;16:5455. doi: 10.1038/s41467-025-59575-8 (PMC12218259; doi:10.1038/s41467-025-59575-8)
Supplement: Supplementary file 1 — Supplementary Information [file 41467_2025_59575_MOESM1_ESM.pdf]

Supplementary Information

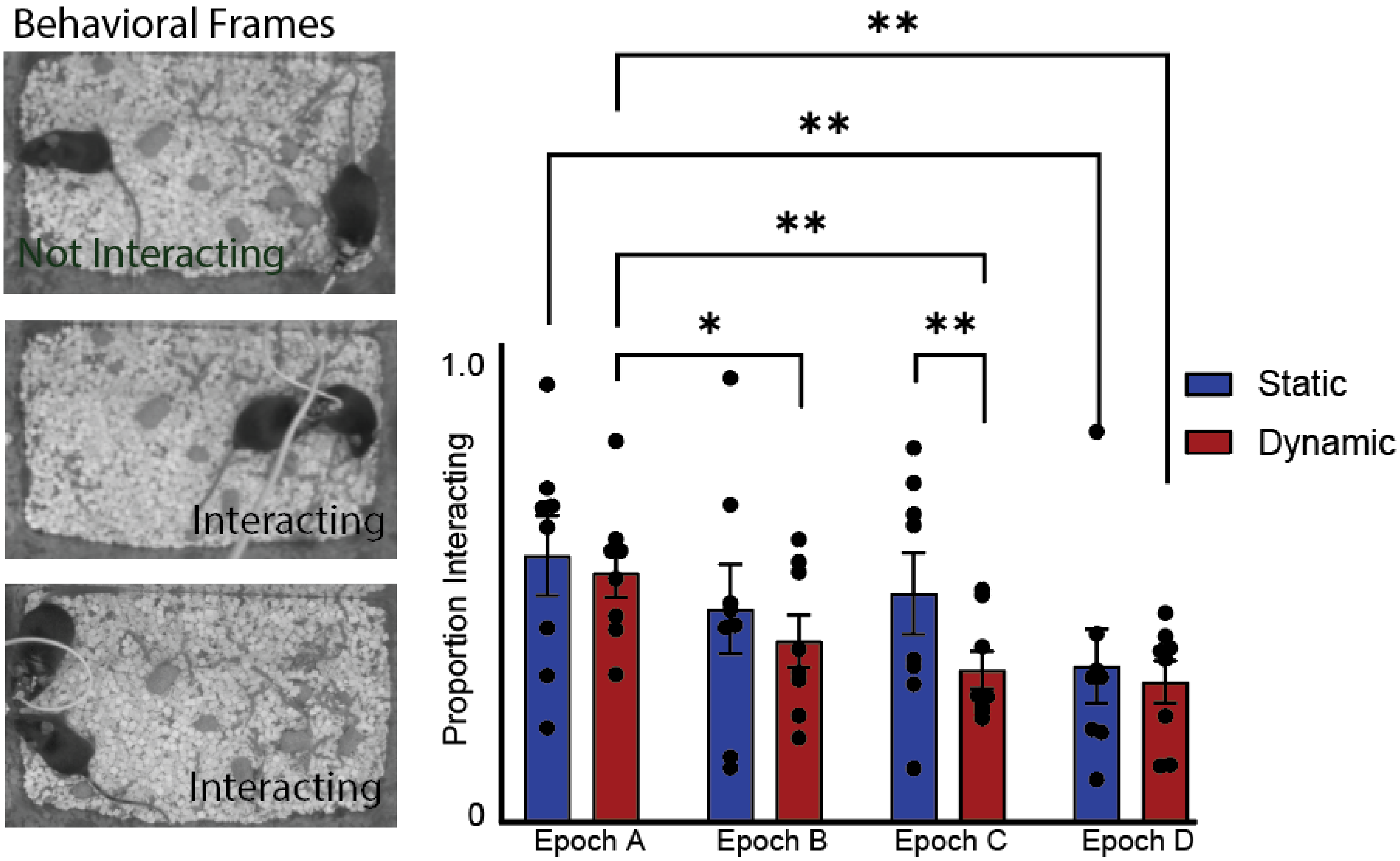

Supplementary Figure 1: Social Interaction Time vs Epoch.

Social behavior was manually scored as interacting frames or non-interacting frames. Left panels demonstrate examples of interacting and non-interacting frames. Right: bar graph depicting the proportion of frames scored as ‘interacting’ by epoch for static and dynamic context experiment (2 Way ANOVA repeating by epoch and experimental day ($p < 0.001$ for epoch, $p = 0.25$ for experimental day and 0.34 for interaction. * indicates $p < 0.05$; ** indicates $p < 0.01$). Source data are provided as a Source Data file.

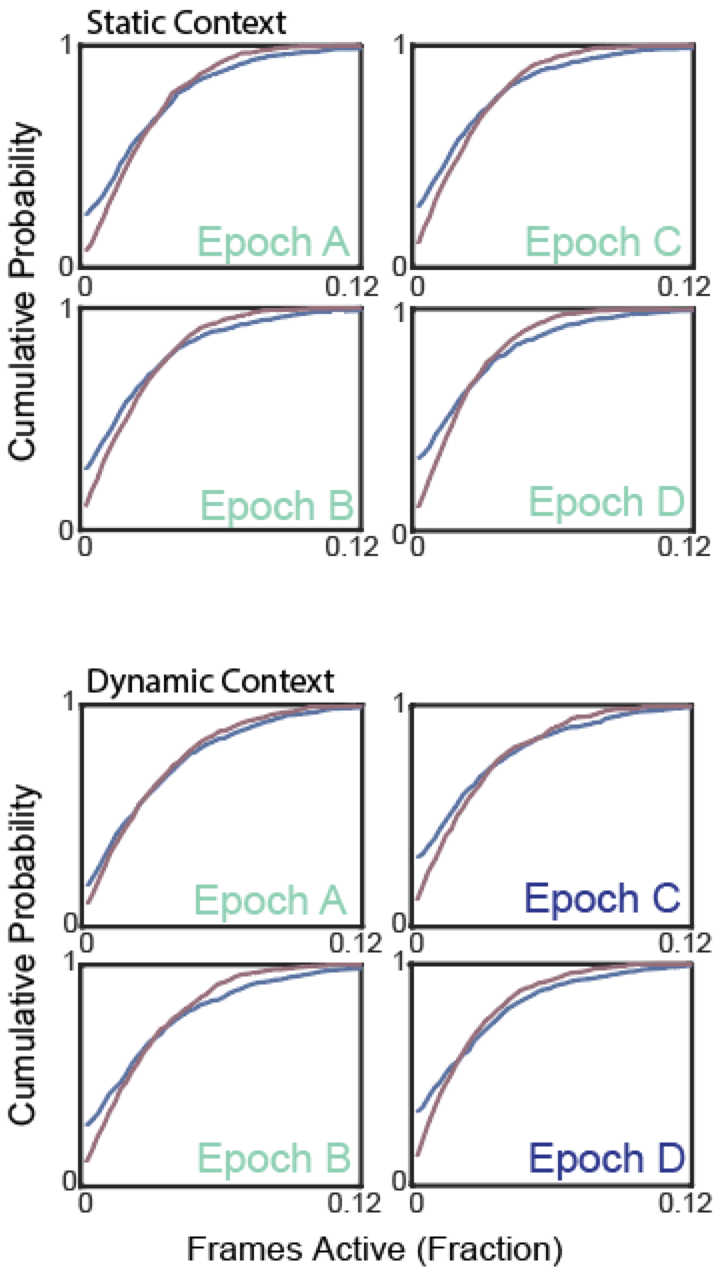

Supplementary Figure 2: Dynamic changes in prefrontal network activity driven by social interaction regardless of context.

Top. Cumulative probability function depicting the proportion of frames that each neuron was active during social (blue) and non-social frames (red) over the entire experiment (left) or during individual epochs (right). Social interaction was associated with an increase in both the proportion of highly active and inactive neurons in the static context experiment (n = 688 neurons from 8 mice. All comparisons nonsignificant by two-tailed paired t-test; All comparisons $p < 0.0001$; KS test).

Bottom. Cumulative distributions of activity for dynamic context experiment (Epoch A: NS by paired t test, $p < 0.001$, KS test; Epoch B: NS by paired t test, $p < 0.0001$, KS test; Epoch C: NS by two-tailed paired t test, $p < 0.0001$, KS test; Epoch D: NS by paired t test, $p < 0.0001$, KS test; n = 632 neurons from 8 mice). Source data are provided as a Source Data file.

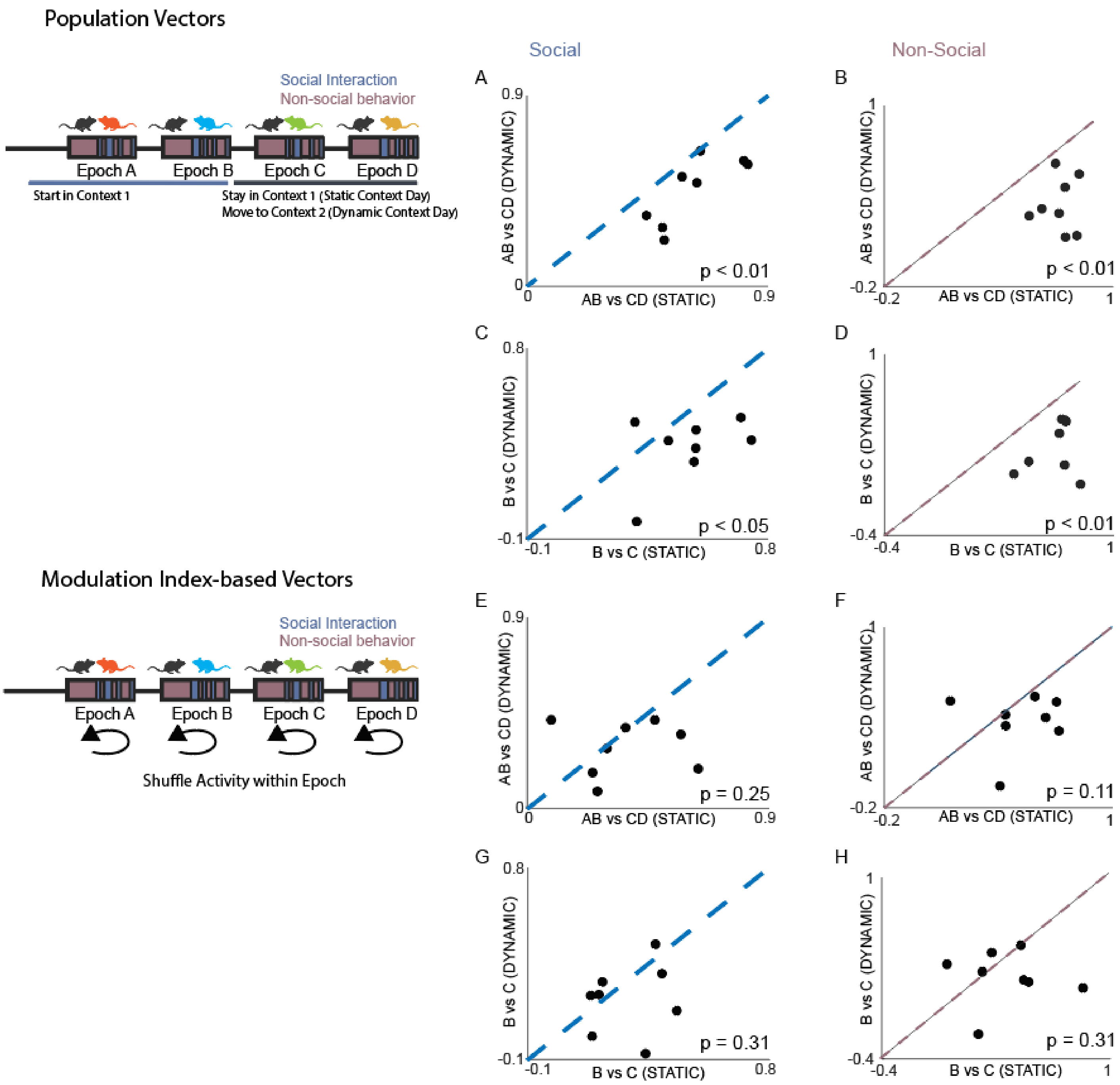

Supplementary Figure 3: Neurons are persistently modulated by social interaction despite context dependent remapping of prefrontal activity. We first calculated population vectors corresponding to the mean activity within each behavior by epoch.

A. Scatterplot of the similarity between population vectors generated by averaging activity across social epochs A and B vs population vectors generated by averaging activity across social epochs C and D in the static context (x axis) and dynamic context (y-axis). $p < 0.01$, two-sided signed-rank; n = 8 mice.

B. Scatterplot of the similarity between population vectors generated by averaging activity across non-social epochs A and B vs population vectors generated by averaging activity across non-social epochs C and D in the static context (x axis) and dynamic context (y-axis). $p < 0.01$, two-sided signed-rank; n = 8 mice.

C. Scatterplot of the similarity between population vectors generated by averaging activity within social epochs B vs population vectors generated by averaging activity within social epoch C in the static context (x axis) and dynamic context (y-axis). $p < 0.05$, two-sided signed-rank; $n = 8$ mice.

D. Scatterplot of the similarity between population vectors generated by averaging activity within non-social epochs B vs population vectors generated by averaging activity within non-social epochs C in the static context (x axis) and dynamic context (y-axis). $p < 0.01$, two-sided signed-rank; $n = 8$ mice.

We next calculated population vectors generated by calculating the Modulation Index for each neuron across each behavior and epoch.

E. Scatterplot of the similarity between modulation vectors generated from the social modulation index generated from epochs A and B vs population vectors corresponding to the social modulation index generated from epochs C and D in the static context (x axis) and dynamic context (y-axis). $p = 0.25$, two-sided signed-rank; $n = 8$ mice.

F. Scatterplot of the similarity between modulation vectors generated from the non-social modulation index generated from epochs A and B vs population vectors corresponding to the non-social modulation index generated from epochs C and D in the static context (x axis) and dynamic context (y-axis). $p = 0.11$, two-sided signed-rank; $n = 8$ mice.

G. Scatterplot of the similarity between modulation vectors generated from the social modulation index generated from epoch B vs population vectors corresponding to the social modulation index generated from epoch C in the static context (x axis) and dynamic context (y-axis). $p = 0.31$, two-sided signed-rank; $n = 8$ mice.

H. Scatterplot of the similarity between modulation vectors generated from the non-social modulation index generated from epoch B vs population vectors corresponding to the non-social modulation index generated from epoch C in the static context (x axis) and dynamic context (y-axis). $p = 0.31$, two-sided signed-rank, $n = 8$ mice. Source data are provided as a Source Data file.

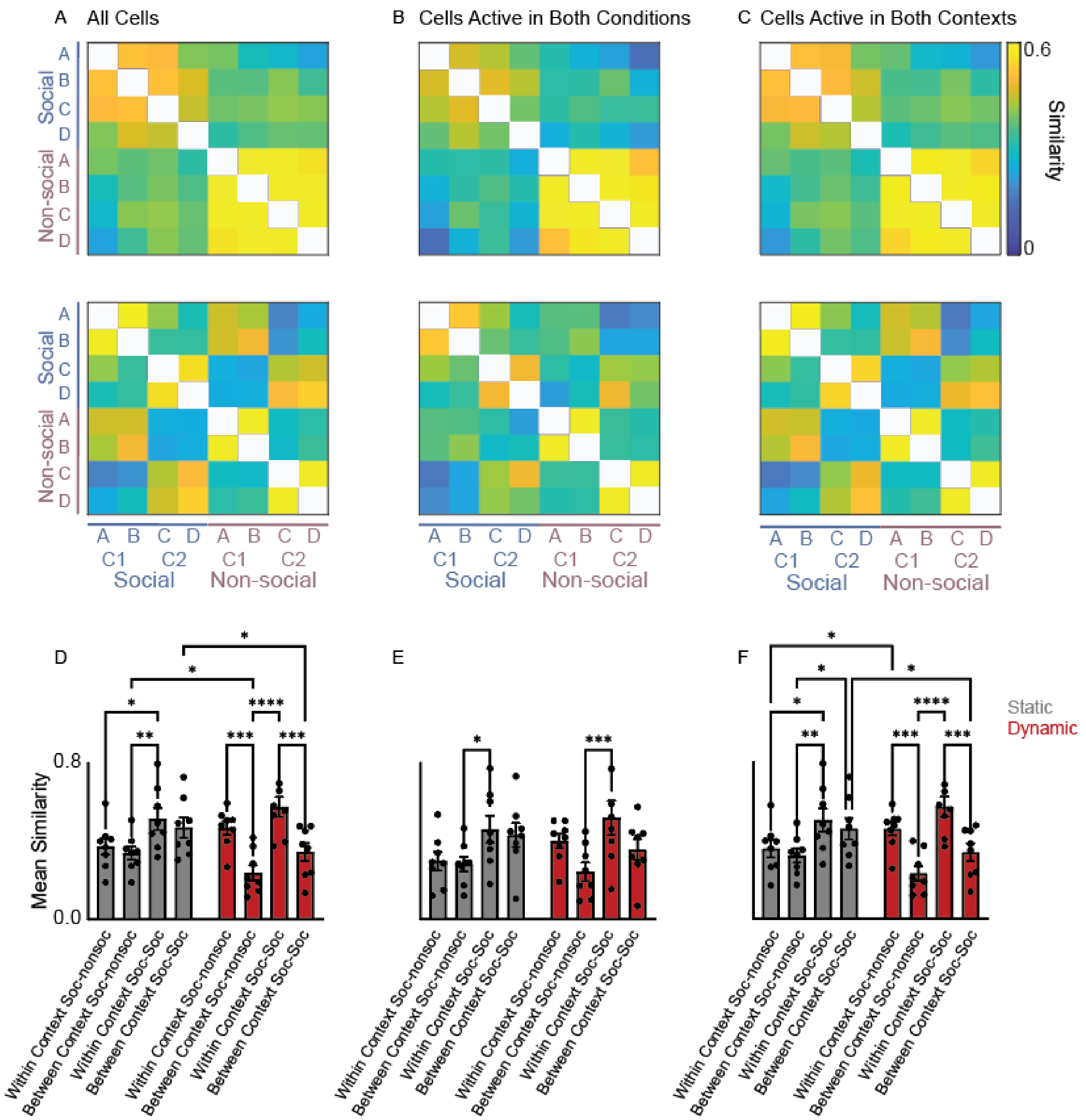

Supplementary Figure 4: Context-dependent social encoding does not depend on cells which are active in only a single context.

A-C. We examined the similarity of (correlation between) population activity vectors using either: all cells (A); only cells that were active within both vectors being compared (i.e., in both contexts) (B); only cells that were active for at least one behavioral condition (either social or nonsocial) in each context (context 1: epochs A & B; context 2: epochs C & D) (C).

D. Bar graph of the similarities of (correlations between) population activity vectors for social or nonsocial behaviors in different contexts (gray and red bars correspond to the static and dynamic context experiments, respectively). In the dynamic context experiment, social vectors are more similar to social vectors from the

same context than to cross-context social vectors; nonsocial vectors are also more similar to social vectors from the same context than to cross-context social vectors (2-way ANOVA, $p = 0.69$ for Experiment Day, $p < 0.005$ for for Comparison Type (social-social and social-nonsocial comparisons within or between context) and $p < 0.01$ for Experiment Day x Comparison Type, posthoc testing performed using Tukey's multiple comparison test).

E. Analogous to D, but for each comparison between a pair of population activity vectors, we excluded cells that were not active in both vectors (as in panel B). Though there remains a trend toward social vectors being more similar to within-context social vectors (compared to between-context comparisons) and non-social vectors also being more similar to within-context social vectors (compared to between-context comparisons) in the dynamic context experiment this is no longer significant (2-way ANOVA $p = 0.88$ for Experiment Day, $p < 0.005$ for Comparison Type (social-social and social-nonsocial comparisons within or between context) and $p = 0.16$ for Experiment Day x Comparison Type, posthoc testing performed using Tukey's multiple comparison test, n = 6 mice).

F. Analogous to D, but in this case we excluded cells that were not active in both contexts (similar to panel C; unlike E, cells were not necessarily active in each vector being compared). In the dynamic context experiment, social vectors are more similar to social vectors from the same context than the other context, and non-social vectors are also more similar to social vectors from the same context than the other context (2-way ANOVA $p = 0.81$ for Experiment Day (Static context vs Dynamic context), $p < 0.005$ for for Comparison Type (social-social and social-nonsocial comparisons within or between context)and $p < 0.01$ for Experiment Day x Comparison Type, posthoc testing performed using Tukey's multiple comparison test). * indicates $p < 0.05$; ** indicates $p < 0.01$; *** indicates $p < 0.001$; **** indicates $p < 0.0005$. Source data are provided as a Source Data file.

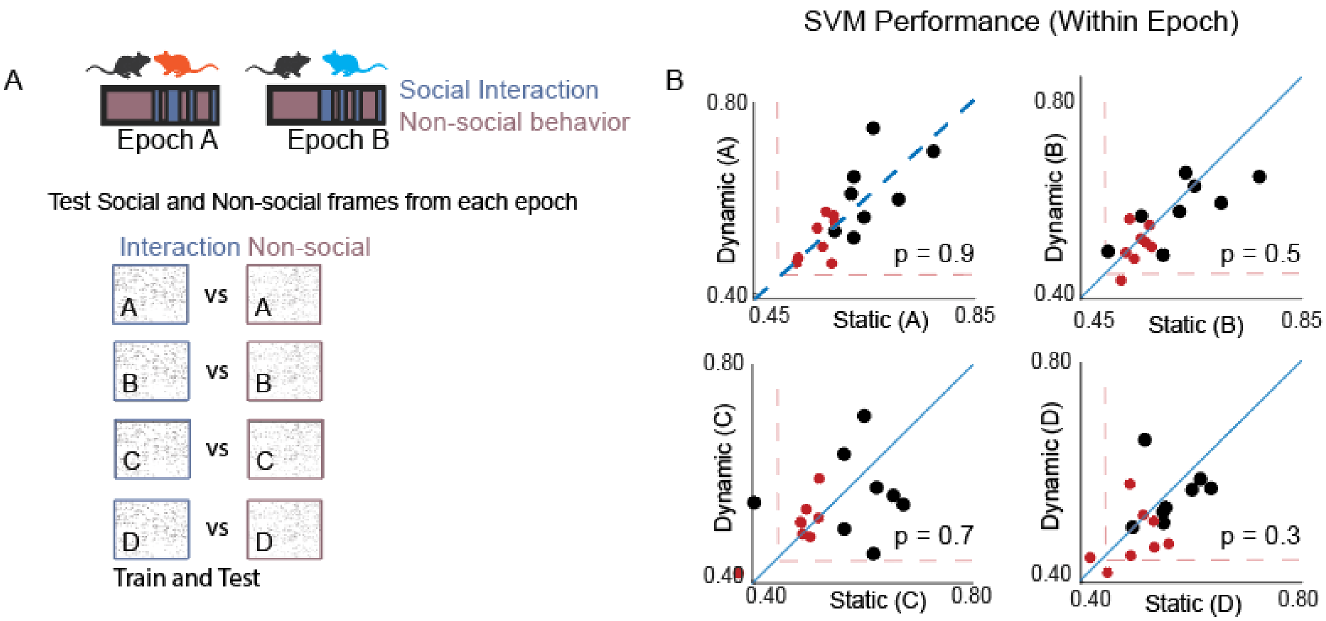

Supplementary Figure 5: Linear Decoder discriminates social interaction regardless of context.

A. A Support Vector Machine classifier was trained using binarized calcium data to discriminate frames corresponding to social interaction from non-social bouts within each epoch.

B. Bar graph of the within-Epoch classifier performance for Epoch A-D. (Epoch A p = 0.9, two-sided signed-rank); Epoch B mean performance static context p = 0.5, two-sided signed-rank; Epoch C p = 0.7 two-sided signed-rank; Epoch D p = 0.3, two-sided signed-rank; n = 8 mice for all comparisons). Source data are provided as a Source Data file.

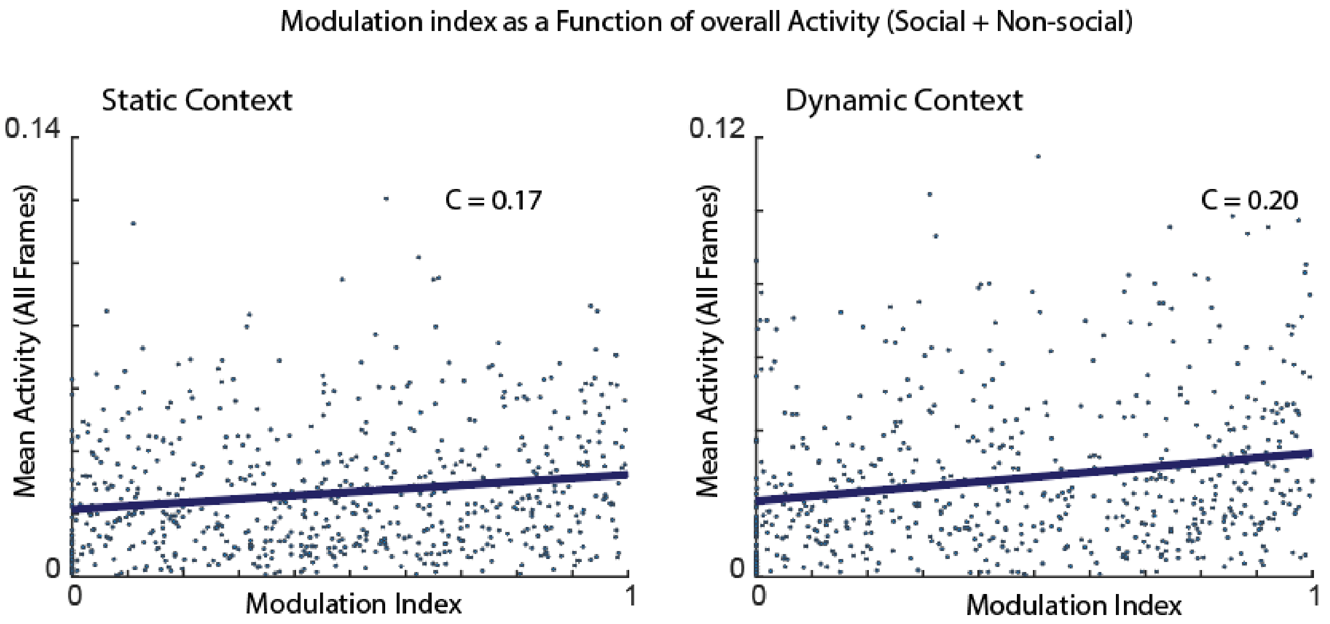

Supplementary Figure 6: Modulation Index vs mean activity.

We plotted the mean activity of each neuron (Y axis) as a function of Modulation Index (X-axis) for Static Context (left) and Dynamic Context (right) experiment day. N = 688 neurons from 8 mice (Static) and 632 neurons from 8 mice (Dynamic). Source data are provided as a Source Data file.

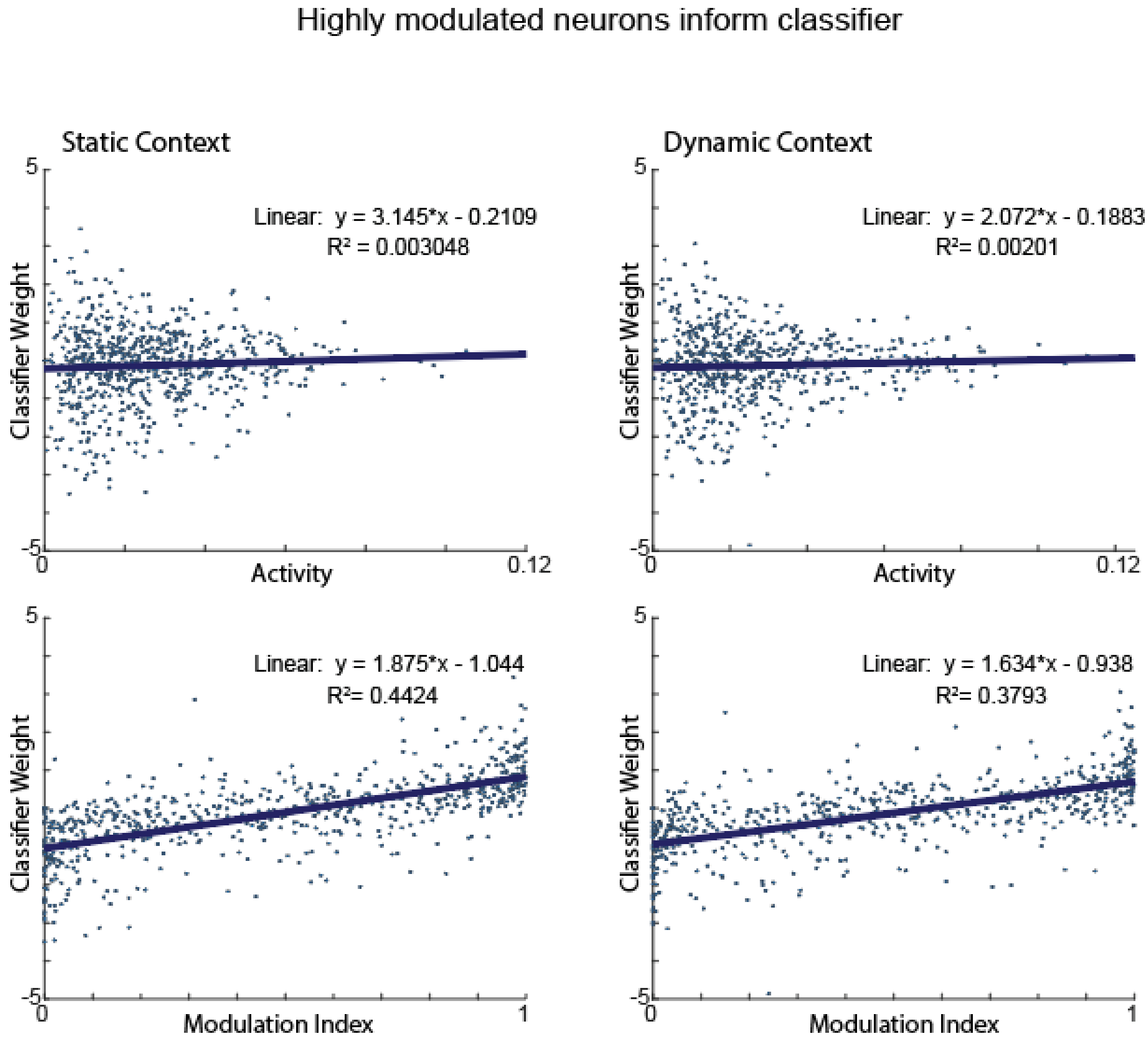

Supplementary Figure 7: Highly modulated neurons inform classifier.

Top: We plotted the weight given to each neuron during training (X axis) as a function of its mean activity (X-axis) for the Static (left) and Dynamic Context (right) experiment day. Bottom: We plotted the weight given to each neuron during training (X axis) as a function of its calculated modulation index (X-axis) for the Static (left) and Dynamic Context (right) experiment day. ‘Activity’ corresponds to the proportion of frames in which a neuron is active. N = 688 neurons from 8 mice (Static) and 632 neurons from 8 mice (Dynamic). Source data are provided as a Source Data file.

Alone vs Social frames

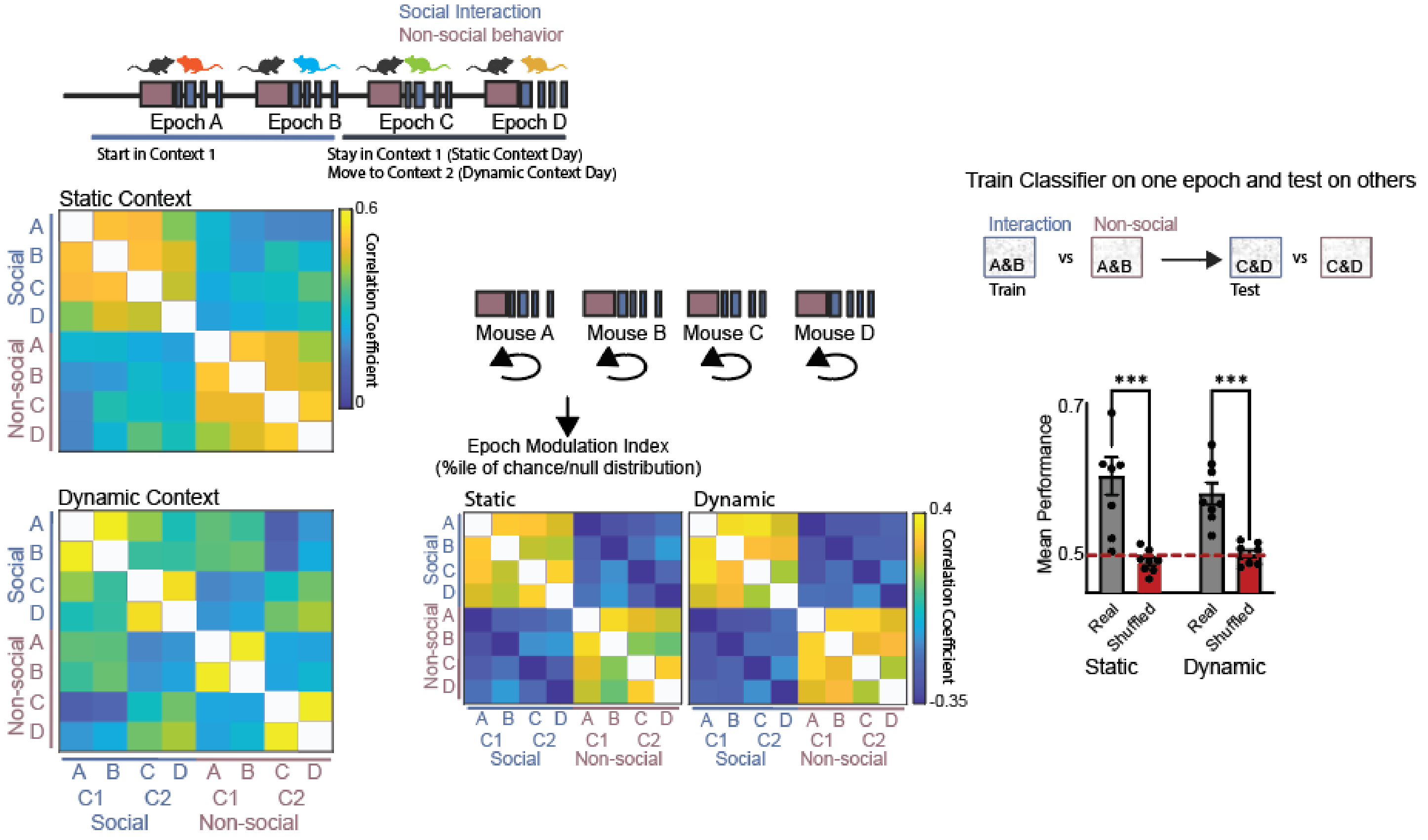

Social frames vs non-social frames after introduction of conspecific

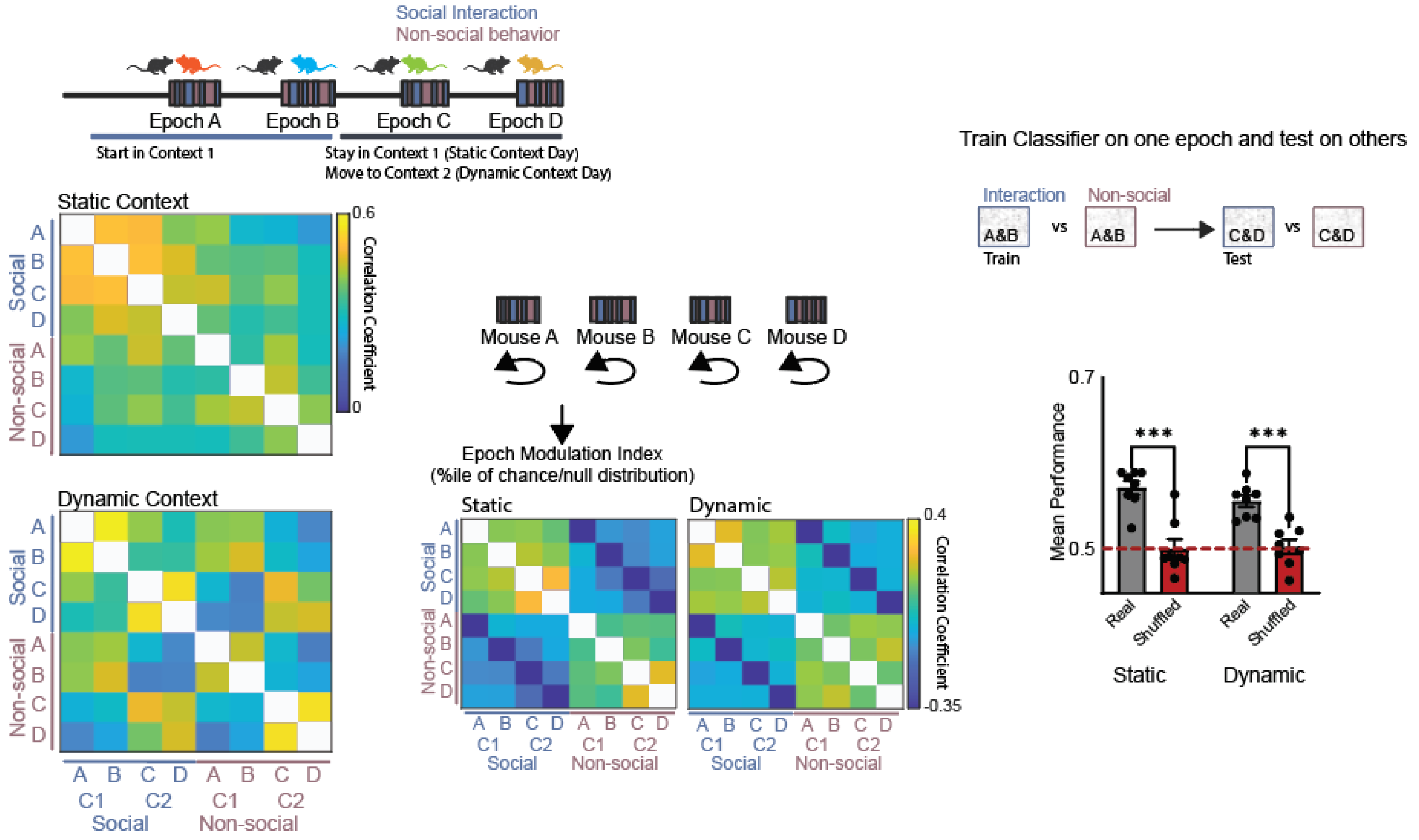

Supplementary Figure 8: Activity underlying social interaction is distinct from activity when mouse is alone or not interacting but in the presence of conspecific.

We generated population vectors in which non-social time was defined only as periods in which the mouse was alone (top) or only when there was a conspecific present (but between periods of interaction). We observed qualitatively similar results in both cases. Left panels show population vector comparisons. Middle panels show modulation vector comparisons. Right panels show classifier trained to predict periods of social interaction during epochs A&B and then test on calcium data from epochs C&D (For alone vs social comparison,. 2-way RM ANOVA $p = 0.67$ for experimental day and $p < 0.001$ for shuffled data. For nonsocial vs social when conspecific present: 2-way RM ANOVA $p = 0.72$ for experimental day and $p < 0.005$ for shuffled data; asterisks represent results of Tukey's multiple comparisons test. N = 8 mice). *** indicates $p < 0.001$. Source data are provided as a Source Data file.

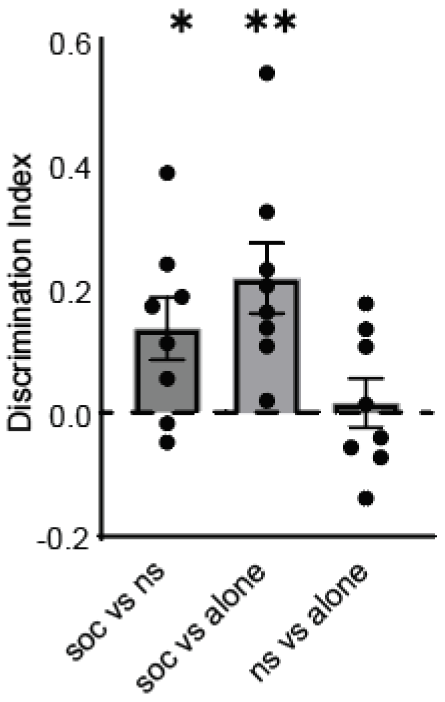

Supplementary Figure 9: Distinct neuronal ensembles permit discrimination of social behavior

We quantitatively compared population vectors generated in Supplementary Figure 8 to examine whether neuronal activity associate with periods of active social interaction ('soc') was distinct from that associated with periods in which the mouse was alone ('alone') or only when a juvenile conspecific was present, but the subject mouse was not actively engaged in interaction ('ns'). We calculated the 'Discrimination Index,' defined as the mean similarity of population activity vectors associated with different instances of the same behavior during different epochs minus the mean similarity of all activity vectors associated with two different behaviors. The Discrimination Index was significantly above zero when comparing periods in which the mouse was activity engaged in social interaction to periods in which te mouse was either alone or in the presence of a conspecific but not actively interacting (soc vs. alone: $p < 0.01$, two-tailed *t*-test; soc vs. ns: $p < 0.05$, *t*-test). By contrast the Discrimination Index was near zero for periods in which the mouse was alone compared to when it was in the

presence of a conspecific but not interacting: (ns vs. alone: $p = 0.71$). Source data are provided as a Source Data file.

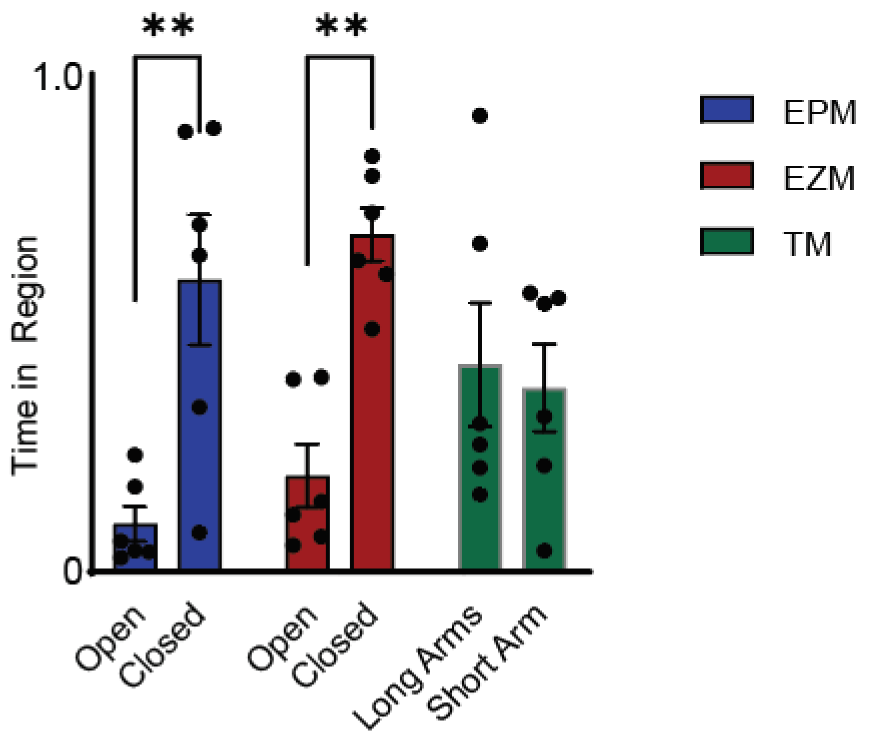

Supplementary Figure 10: Quantification of behaviors in anxiety-provoking mazes.

Bar graph depicting the proportion of time spent in the open and closed regions of the Elevated Plus Maze (Blue), Open and closed arms of the Elevated Zero Maze (Red), and Long and Shorty arms of the T Maze (Green). Mice spent significantly more time in the closed arms of the EPM and EZM, but there was no difference in the time spent in the long vs short arm of the T maze (Proportion of time EPM Open Arms 9.7+/-3.5%, Closed Arms 58.9+/-13.2%, Proportion of time spent in EZM Open Arms 19.4+/-6.4%, EZM Closed arms 68.0+/-5.4%, Tmaze Long 41.8+/-12.4, Tmaze Short 37.1+/-8.8%, $p = 0.0005$, ANOVA; posthoc testing with Šídák's multiple comparisons test, $n = 6$). ** indicates $p < 0.01$. Source data are provided as a Source Data file.

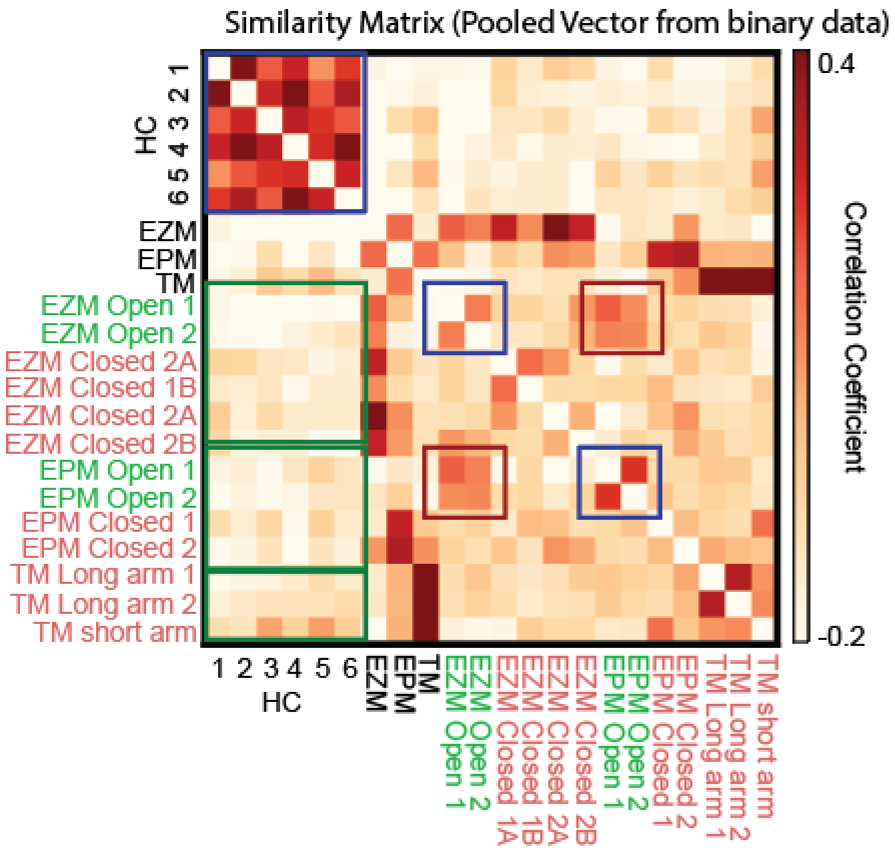

Supplementary Figure 11: Context invariant representation of anxiety-related information.

We generated a similarity matrix by computing correlations between population activity vectors corresponding to different epochs / specific arms. In this case population activity vectors consisted of all neurons pooled across mice. (HC vs HC epochs correlation coefficient 0.27, HC epochs vs all other epochs correlation coefficient -0.15, HC vs EZM correlation coefficient -0.16, HC vs EPM correlation coefficient -0.14, HC vs T-maze -0.09, all EZM subregions vs all EZM subregions correlation coefficient -0.01, all EPM subregions vs all EPM subregions correlation coefficient = 0.01, all T-maze subregions vs all T-maze subregions correlation coefficient 0.12, EZM open vs EPM open correlation coefficient 0.13, EZM Open vs T-Maze arms -0.08, EPM Open vs T-maze arms correlation coefficient -0.06, n = 633 neurons from 6 mice).

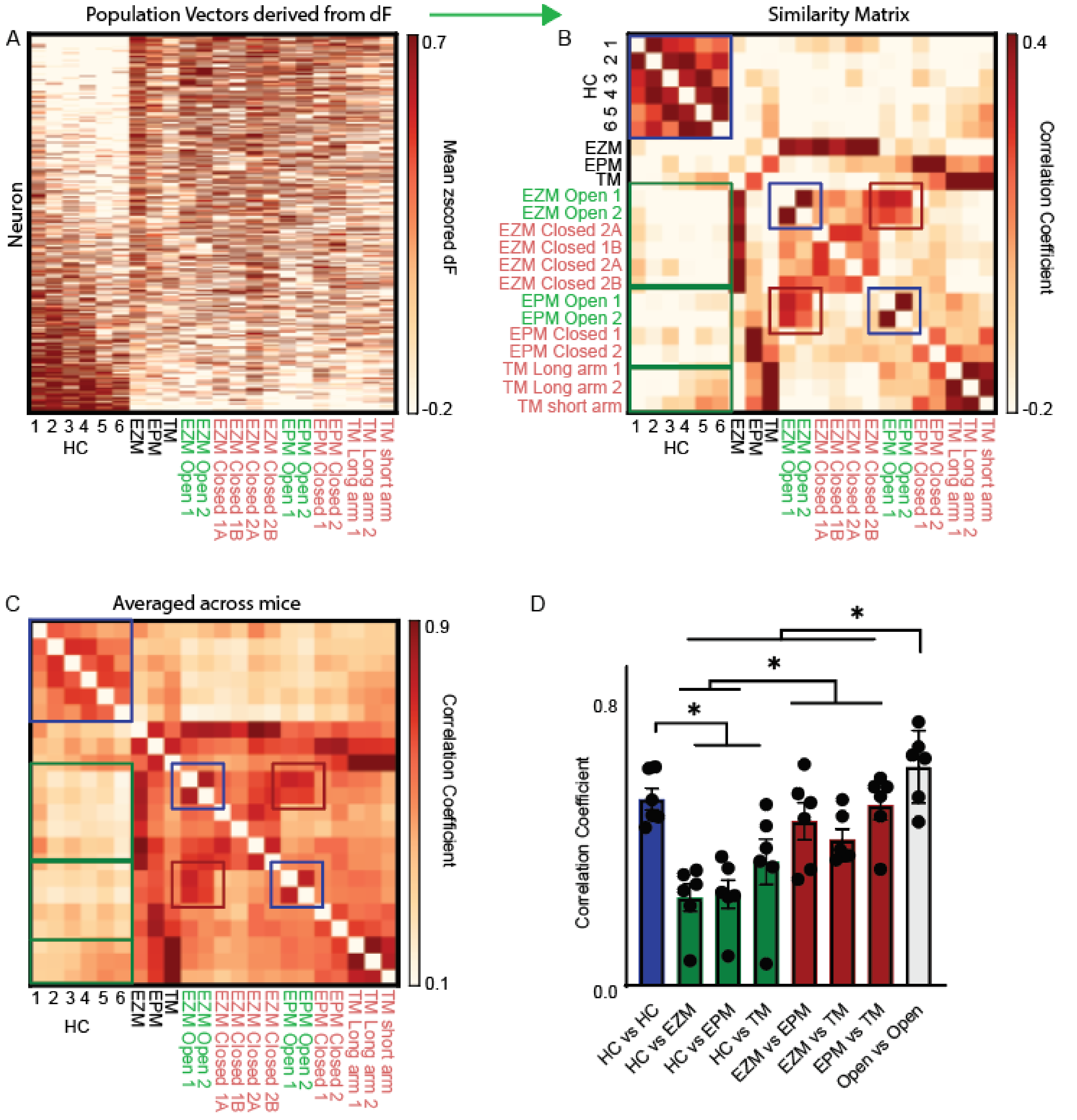

Supplementary Figure 12: Persistence of ensemble representations of anxiety-related information across contexts.

A. Behavior was subdivided into each home cage epoch and the time mice spent within each arm of the EZM, EPM, and T-Maze. EZM closed arms were further divided by the direction of travel (clockwise, CW; counter-clockwise, CCW). We generated population activity vectors for each epoch and arm by calculating the mean of the z-scored calcium trace for each neuron within each maze and each subregion (arm). Neurons were concatenated across all mice and then sorted the activity of neurons based on their average activity during home cage epochs; As in Figure 3 and 4 this revealed obvious differences between vmPFC activity during home cage exploration and time spent in EZM, EPM, or T-Maze.

B. We generated a similarity matrix by computing correlations between population activity vectors generated from averaged calcium traces corresponding to different epochs / specific arms. Population activity vectors were generated by concatenating all neurons from 6 mice (HC vs HC epochs correlation coefficient 0.31, HC epochs vs all other epochs correlation coefficient -0.18, HC vs EZM correlation coefficient -0.20, HC vs EPM correlation coefficient -0.18, HC vs T-maze -0.13, all EZM subregions vs all EZM subregions correlation coefficient 0.15, all EPM subregions vs all EPM subregions correlation coefficient = 0.06, all T-maze subregions vs all T-maze subregions correlation coefficient 0.37, EZM open vs EPM open correlation coefficient 0.24, EZM Open vs T-Maze arms-.12, EPM Open vs T-maze arms correlation coefficient -0.09, n = 633 neurons from 6 mice).

C. Same as B but here we generated an averaged similarity matrix by computing correlations between population activity vectors corresponding to different epochs / specific arms for each mouse, then averaging the resulting 6 similarity matrices..

D. Bar graph depicting the averaged similarity between the 6 home cage epochs for each mouse (mean correlation coefficient 0.58+/-0.03), each context and the adjacent two home cage epochs (EZM vs HC mean correlation coefficient 0.28+/-0.04; EPM vs HC mean correlation coefficient 0.29+/-0.04; Tmaze vs HC 0.38+/-0.06), and the population activity underlying exploration of each of the three contexts (EZM vs EPM mean correlation coefficient 0.52+/-0.06, EZM vs Tmaze 0.46+/-0.03, EPM vs Tmaze 0.57+/-0.04). ANOVA ($p < 0.0001$) with posthoc false discovery rate control using Benjamini, Krieger and Yekutieli). Asterisks represent discoveries. Source data are provided as a Source Data file.

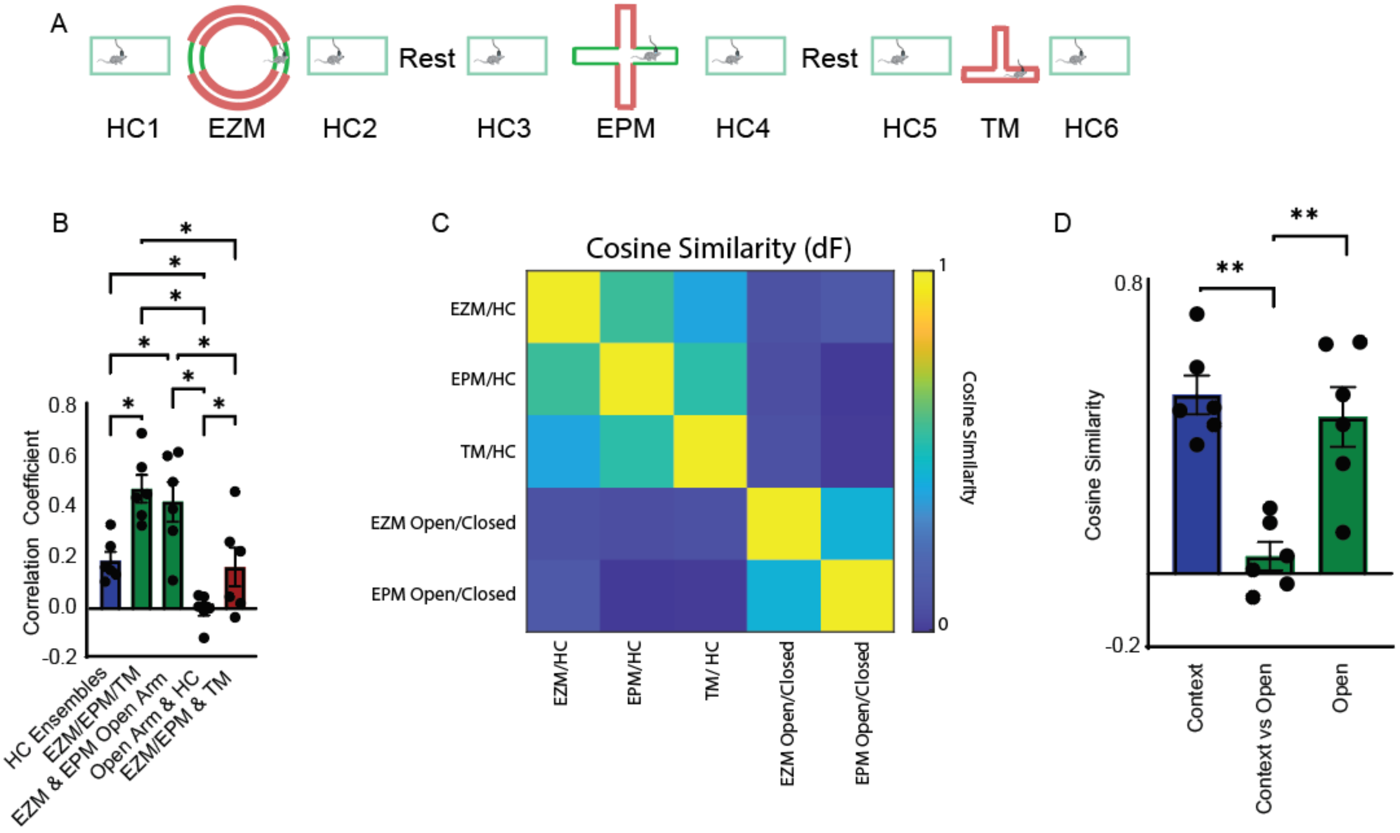

Supplementary Figure 13: Near-orthogonal representations of anxiety- and context-related information.

A-B. We compared modulation index derived ensembles underlying distinct behaviors and locations generated from the continuous calcium traces of each neuron. We generated HC vs HC ensembles by comparing activity in HC epoch 1vs2, 3vs4, and 5vs6. Comparison of the resulting vectors revealed little overlap (mean correlation coefficient 0.19+/-0.03). Context specific vectors were generated by comparing each context to adjacent periods of home cage exploration (mean correlation coefficient 0.48+/-0.06) and open arm specific ensembles for the EZM and EPM were generated by comparing activity during open arm exploration to closed arm exploration. Ensembles for open arm exploration were similar to ensembles generated for the open arm of the EPM (mean correlation 0.43+/-0.08). Open arm ensembles were distinct from HC vs HC ensembles (mean correlation coefficient -0.00+/-0.02). Finally, we quantified the similarity with which ensemble activity was modulated by exploration of the open arm in the EZM or EPM and the similarity with which ensemble activity was modulated as mice explored either of the two long arms of the Tmaze (mean correlation coefficient 0.17+/-0.07). Comparisons were made using ANOVA ($p < 0.0001$) with false discovery rate controlled using Benjamini, Krieger, and Yekutieli). Asterisks represent discovery.

C. To determine the similarity of population activity underlying context and anxiety-related behavior, we calculated the cosine similarity between context-specific vectors and anxiety-related vectors. This data is plotted in a 5x5 matrix containing pairwise comparisons made between each of three context-specific vectors and each of the two anxiety-specific vectors.

D. Bar graph depicting the mean cosine similarity within and between context- and anxiety-specific population vectors (mean cosine similarity context vs context: 0.49+/-0.05, mean cosine similarity context vs open arm: 0.05+/-0.04, mean cosine similarity open vs open: 0.43+/-0.08). RM ANOVA $P < 0.001$, posthoc Tukey's multiple comparisons test. ** indicates $p < 0.01$. Source data are provided as a Source Data file.

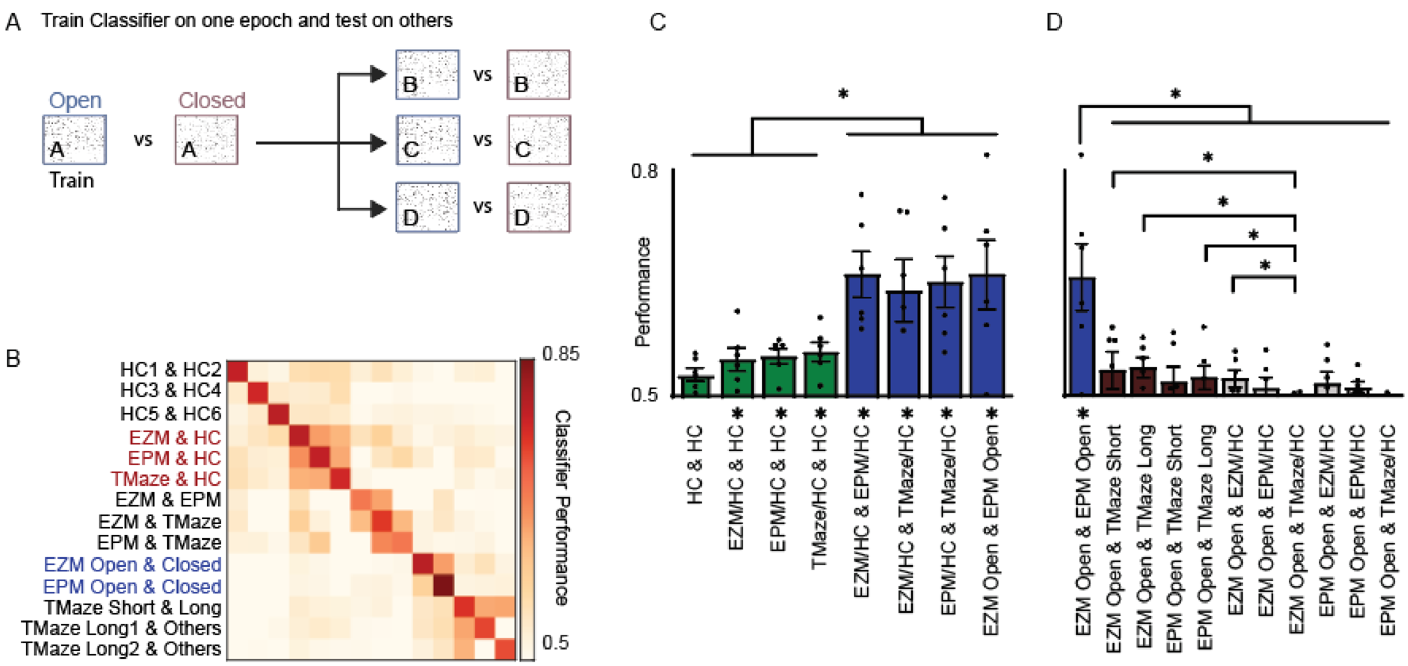

Supplementary Figure 14: Decoding of behavioral states across context.

A. We trained a linear classifier to distinguish frames corresponding to different mazes (ie home cage or EZM), epochs (home cage epoch 1 & 2), or subregions within specific mazes (ie EZM open & closed arms). In each case models were trained on binarized activity data corresponding to a given pair of behavioral regions or epochs, then tested the trained model on all 14 denoted comparisons (200 iterations, 0 hold out). Shuffled data was generated by circularly shuffling the 'testing' dataset, and reduced classifier performance to chance for all comparisons (not shown).

B. 14x14 matrix of classifier accuracy for all combinations of training and testing data.

C. Bar graph of the mean performance of the SVM trained and tested on the corresponding pairs. Here 'HC' corresponds to pairs of home cage epochs adjacent to a single maze (ie 'HC1 & HC2'), and X/HC indicates a model trained to distinguish frames corresponding to exploration of 'X' from frames spent exploring the adjacent two home cage epochs. The classifier performed significantly above chance when trained to distinguish any of the three mazes from adjacent home cage epochs and then tested on any of the other mazes. Notably, the classifier was equally able to distinguish open arms from closed arms when trained on the EZM or EPM and tested on the opposing maze. Data from panels C and D are subsets of same analysis; $p < 0.0001$ for ensemble comparison, p =< 0.01 for shuffling, and $p < 0.0001$ for interaction; 2 WAY RM ANOVA with posthoc control of false discovery using two stage linear procedure of Benjamini, Krieger, and Yekutieli. Shuffled data not shown but * below x-axis indicate comparisons that differ significantly from shuffled data.

D. Bar graph of within-context, and within-context vs context comparisons. The classifier performed above chance only when trained to distinguish the open arm of one maze and tested on the opposing maze. All other comparisons were indistinguishable from shuffled data.. Shuffled data not shown but * below x-axis indicate comparisons that differ significantly from shuffled data. Asterisks represent discoveries. Source data are provided as a Source Data file.

All Frames

	Static F1	SEM	Dynamic F1	SEM	Static Precision	SEM	Dynamic Precision	SEM	Static Recall	SEM	Dynamic Recall	SEM
Real	0.62	0.02	0.60	0.02	0.63	0.02	0.62	0.02	0.63	0.03	0.61	0.03
Shuffled	0.47	0.01	0.47	0.01	0.52	0.01	0.51	0.01	0.45	0.02	0.45	0.02

Epoch A only

	Static F1	SEM	Dynamic F1	SEM	Static Precision	SEM	Dynamic Precision	SEM	Static Recall	SEM	Dynamic Recall	SEM
Real	0.59	0.03	0.58	0.03	0.61	0.02	0.61	0.03	0.60	0.04	0.58	0.04
Shuffled	0.46	0.02	0.48	0.01	0.51	0.01	0.52	0.01	0.45	0.02	0.47	0.02

ABCD Within, Averaged

	Static F1	SEM	Dynamic F1	SEM	Static Precision	SEM	Dynamic Precision	SEM	Static Recall	SEM	Dynamic Recall	SEM
Real	0.55	0.02	0.53	0.02	0.57	0.02	0.57	0.02	0.53	0.03	0.51	0.02
Shuffled	0.43	0.02	0.44	0.01	0.49	0.01	0.50	0.01	0.41	0.03	0.42	0.02

Train-Test Between Epochs

Static F1

	A	B	C	D
A	0.79	0.48	0.49	0.45
B	0.51	0.74	0.50	0.49
C	0.47	0.45	0.77	0.52
D	0.44	0.46	0.40	0.72

Static Precision

	A	B	C	D
A	0.77	0.57	0.60	0.53
B	0.60	0.75	0.57	0.61
C	0.57	0.57	0.74	0.58
D	0.58	0.56	0.57	0.79

Static Recall

	A	B	C	D
A	0.81	0.42	0.44	0.42
B	0.47	0.76	0.47	0.45
C	0.43	0.40	0.80	0.47
D	0.37	0.40	0.34	0.69

Dynamic F1

	A	B	C	D
A	0.73	0.55	0.51	0.48
B	0.53	0.72	0.45	0.48
C	0.47	0.44	0.74	0.51
D	0.47	0.46	0.52	0.69

Dynamic Precision

	A	B	C	D
A	0.73	0.59	0.56	0.53
B	0.59	0.71	0.55	0.56
C	0.60	0.59	0.76	0.59
D	0.53	0.56	0.61	0.71

Dynamic Recall

	A	B	C	D
A	0.74	0.53	0.51	0.47
B	0.48	0.74	0.41	0.45
C	0.43	0.40	0.75	0.46
D	0.44	0.42	0.46	0.70

Supplementary Table 1: Accuracy of classifiers. To evaluate the performance of the SVM trained to classifiy social interaction frames we examined the F1 Score, Precision, and Recall for each model trained and tested on real or shuffled data from static and dynamic experiment days. The classifier statistics aligned with the performance of the classifier and we observed the highest F1, Precision, and Recall when classifier was trained and tested on all frames from the experiment (top). The performance dropped somewhat when the classifier was trained and tested only within a single epoch (middle shows data from Epoch A only and data averaged across epochs A, B, C and D; n = 8 mice). We next examined errors made by the classifier when trained on 100 percent of the frames from one epoch and tested on 100 percent of the frames from other epochs. Here precision was generally higher than 0.5 whereas recall was less than 0.5 suggesting that frames predicted to be social were generally accurate but that a substantial number of frames were missed (n = 8, mean is displayed).
