# Supplementary material for: Context-invariant socioemotional encoding by prefrontal ensembles
Source: Nat Commun. 2025 Jul 1;16:5455. doi: 10.1038/s41467-025-59575-8 (PMC12218259; doi:10.1038/s41467-025-59575-8)
Supplement: Supplementary file 3 — Transparent Peer Review file [file 41467_2025_59575_MOESM3_ESM.pdf]

nature portfolio

Peer Review File

Context-invariant socioemotional encoding by prefrontal ensembles

Corresponding Author: Dr Nicholas Frost

This manuscript has been previously reviewed at another journal. This document only contains information relating to versions considered at Nature Communications.

This file contains all reviewer reports in order by version, followed by all author rebuttals in order by version.

Version 0:

Reviewer comments:

Reviewer #1

(Remarks to the Author)
Frost et al. used calcium imaging to examine how prefrontal neurons encode social and context information in different environments with four novel mice. Their analyses showed that representations of two types of information are partly overlapped but almost independent. In addition, they tested representation patterns of context and anxiety-related information. These results provide crucial insights into how prefrontal cortex multiplexes parallel information to achieve socioemotional and context-invariant encoding. Overall, the experiments are well designed, and I am very enthusiastic about this manuscript. However, there are several analytical issues that need to be clarified. Especially, reaching the conclusion that "prefrontal neurons show orthogonal representations" based on their results alone seems a significant logical jump.

(Major)
(1) Overall, the authors defined two periods: social and non-social. They described "'Social interaction' was limited to timepoints when the mouse was actively interacting with the novel juvenile, whereas 'nonsocial' included both timepoints when the juvenile was present but the mouse was not engaged in interaction plus the preceding period when the mouse was alone (before the juvenile was introduced)." There is a need to verify whether this definition is appropriate.
(1-1) First, please further explain how they defined "social bouts". In the methods, they described it was scored manually, but what were more objective factors such as the distance, angle, and posture of the mice? As this definition affects all the results, it would be beneficial to have objective scoring metrics rather than subjective ones. However, even if it is unavailable, at the very least, detailed descriptions should be provided. If necessary, please include photos of typical mouse behaviors in the figures.
(1-2) As the authors described, the "nonsocial" periods contain two completely different timepoints: (i) when the juvenile was present but the mouse was not engaged in interaction, and (ii) the preceding period when the mouse was alone. In a strict sense, the former is social environment without social interaction, while the latter is completely nonsocial. Therefore, neuronal activity may be considerably different between the two periods. Before combining these periods (before Fig. 1F), please first provide analyses of neuronal activity to validate and claim that these activity patterns in the two periods are actually identical and could be combined. Otherwise, all analyses may need to be performed by classifying into three periods: social, when the juvenile was present without social interaction, and when it was alone.
(2) Interpretations of overlap and orthogonalization. For example, the authors repeatedly used the term "orthogonalization" (such as in the Abstract), but based on the most important data in Figure 3, this interpretation seems insufficient.
(2-1) Figure 3E shows that, as the author described, there are no significant differences between real and shuffled datasets in the comparison of Soc AB-Context AB. However, these real data seem to have some positive similarities. To more precisely claim that they are orthogonal, in addition to this comparison, statistical tests versus 0 are necessary.
(2-2) Related to this, Figure 3F shows that some neurons seem to more preferentially show both social and context information (as indicated by the dense plots on the upper-right area), suggesting a possibility that these neurons encode both information more than chance levels. To further test whether there are overlapped representations, testing whether these individual proportions in Figure 3G are explained by chance levels or not by using shuffled datasets.
(2-3) In addition, Figure 3G showed the increases in the percentages of neurons showing modulation in the dynamic context, rather than static context as shown by green arrows and the authors concluded that "however this overlap includes neurons modulated in opposing directions, maintaining an approximately orthogonal relationship between these representations".

This is overstatement as there are no data to support that these increases in individual neuronal types are perfectly counterbalanced. Please consider to add some statistics or resume this statement.
(2-4) According to these analyses, they claimed that these representations were “heavily” overlapping. But there seems only a minority of neurons (few %). Is it just a “partial” overlap? Similarly, the term “orthogonalization (cosine similarity=0)” may be rephrased as it may not be actually “orthogonal” but just “independent representations” (In my personal opinion, I believe it might not be necessary to force the use of the term 'orthogonalization).
(3) Figure 3 uses cosine similarity but Figure 5 uses “cosine similarity (-shuffled)”. Why did they present the data in a different manner? As these graphs are most important to interpret orthogonalization or independency of representations compared with chancel level, please be consistent.
(4) Please provide more information about linear classifier. How many cells or what percentage of cells actually contributed to the classification? Was there a correspondence between the number of calcium events (baseline excitability) and the modulation index exhibited by these cells?
(5) Overall, the schematic illustration at the beginning should be improved, especially in Figure 1D. For example, the illustrations could be improved by showing when, where, and how many novel mice were tested in each epoch and day. The best way would be to have someone unfamiliar with this experiment look at it, ensuring they can quickly understand the experimental paradigm from this illustration.
(6) Were the mice tested in Figure 1-3 and Figure 4-6 similar? If so and they could monitor the same neurons across these behavioral tests, they could further obtain new insights into how social interaction-related cells are overalled with anxiety-related cells (optional).
(7) The Discussion part “it is unclear whether the context-invariance of these representations arises within the prefrontal cortex or upstream regions, or via interactions between them”. Please elaborate a bit more on the potential for detailed mechanisms (e.g. brain regions? sparsity of connectivity?).

(Minor)
(1) Overall, why the unit of neuronal activity is frame? Please change it to Hz (or /min) as it is easier to understand in that unit.
(2) The picture in Figure 1B is poor. Please provide clearer photos. In addition, please provide typical calcium fluorescent (dfoverf) traces around Figure 1B.
(3) Overall, in the Legends, please describe how many asterisks correspond to which P-values.
(4) Figure 1G: why they choose this threshold (>7.5% of frames) to define active cells? What is the rationale?
(5) Figure 3D: the two matrices of Static and Dynamic appear very similar. Are they really correct graphs?

Reviewer #2

(Remarks to the Author)
In this manuscript the authors investigate the key role of mPFC in representing social interaction and anxiety-related avoidance across environments. The author imaged neuronal activity in the medial prefrontal cortex of mice engaged in social interactions or anxiety behaviours, and found that population-level activity within the mPFC is dynamically reorganized in a context-dependent manner, while the specific neuronal ensembles with socioemotional information are context-invariant. Moreover, they found the overlapped neuronal ensembles representing social and anxiety-related information maintain a near-orthogonal orientation relative to representations of contextual information.
Although the topic proposed is somewhat novel and potentially important, the experiments were not logically designed. In particular, the classifier and the data analysis on the socioemotional and context-encoding ensembles was ambiguous. The following concerns should be addressed before publication.

Major comments:
1. According to the logic of the project, the authors intended to see if the environment had an impact on social or anxiety like behavior, and the results they obtained were that the cluster was very stable and less susceptible to environmental influences, which may be correct. But social or anxiety involves many processes, and the responsibility or association of each step in the cluster would not be the same.
2. It is not convincing that the authors just performed correlation analysis through simple of the average discharge of cells over a period of time. According to this method, only the average intensity of discharge (or GCaMP) and the average amplitude of brightness change can be seen. This is completely not the case with cluster coding, as the latter needs to be synchronized or sequentially distributed over time. More feasible evidence or literature should be provided to support the reliability of this method.
3. For population activity for social interaction and nonsocial activity, putting social and emotional together may create clusters of interacting neurons, but it is incomprehensible to compare the similarities of several mazes (elevated zero maze, elevated plus maze, and T-shaped maze) later on. The significance of the differences in similarity obtained by the intermediate statistical methods is not clearly explained.

Minor

1. The manuscript wrote that “We performed optical GCaMP measurements of prefrontal circuit activity (Fig. 1A-C) in 8 mice”. I think the author just explore the role of mPFC and didn’t explore the role of circuit, so “circuit” is not accurate.
2. It’s hard to understand Fig 1D according to text. The text and annotations are unclear and lack details. Why the size of blue bars is inconsistent? What do context1 and 2 refer to respectively? What does “expt day” mean?
3. In Fig. 1G,1H,1J,and 1K, the percentage of active neurons during social interaction is significantly more than the percentage of active neurons during non-social stages and it is easy to understand. But why is the percentage of inactive neurons during social interaction also significantly more than the percentage of inactive neurons during non-social stages ?

4. The abbreviation should be explained in the figure annotation, such as the “C1””C2” in the Fig. 1M.
5. Does diagonal blocks from top left to bottom right in Fig.1M represent the similarity value of 1? Why use a dark blue block with a similarity value of 0 to represent it? I have similar question in Fig. 4D.
6. There are many typos that need to be corrected.
(1) Page 2: “Each mouse was tested during interactions interacted with novel sex-matched juvenile conspecifics on two days.” in which the word “interacted” should be deleted.
(2) Page 26: “… indicates a model trained to distingish frames corresponding”, “distingish” should be “distinguish”
(3) Page 23: “Ca2+ activity was recorded throughout a session in which mice explored the elevated zero maze (EZM), elevated plus maze (EPM), and a T-shaped mazel)”, “mazel” should be “maze”.
(4) Page 17: “Bar graph depecting the mean similarity (correlation)”, “depecting” should be “depicting”

Reviewer #3

(Remarks to the Author)
The manuscript by Frost et al. entitled ‘Context-invariant socioemotional encoding by prefrontal ensembles’ seeks to evaluate the role of neuronal ensembles in the medial prefrontal cortex (mPFC) in encoding socioemotional information across different environments. Using deep brain calcium imaging, the authors imaged neuronal activity in the medial prefrontal cortex of mice when they were engaged in social interactions or anxiety-related avoidance within different environments. Their rigorous computational analysis shows that there are overlapping neuronal ensembles that encode context invariant social interaction and anxiety-related representations.

While the premise of this study is interesting, the experimental design is problematic, the behavior of the subjects is not presented or discussed sufficiently, and the main results lack novelty. Moreover, the manuscript is written in very technical language, which is hard to follow, and there is no detailed interpretation of the results. My main comments are below, and they are followed by more technical comments.

Main Comments:
1. There is a wealth of literature describing the neuronal encoding of social behaviors by the mPFC, and a rich literature describing the neuronal encoding of avoidance behavior by the mPFC. The extant literature includes detailed descriptions of the mPFC cell types, circuits, and pathways important for various aspects of social behavior and anxiety-like behavior, whereas the manuscript under review uses a pan-neuronal GCaMP recording strategy and only superficially reports behavior. This severely detracts from the novelty of the study.
2. The mPFC is an extensively studied region, which can be further subdivided into anterior cingulate cortex (ACC), prelimbic (PL) and infralimbic (IL) subdivisions, amongst others. The authors did not clarify from which region they are recording. This is important because these regions are known to differentially regulate the behavior of animals.
3. The anxiety-like behavior test design is busy and seems overdone. There are 6 home cage recordings and 3 mazes in a day, which could be stressful to the mice and affect behaviors. Further, each test is 30 minutes long and there is no acknowledgement of within- or between-test habituation. In fact, there is no behavioral data presented.
4. The authors did not report the number of each sex they recorded from, and there is no mention of whether the data differ by sex. In mice, there are very strong sex differences in both social and anxiety-related behaviors, thereby making sex differences in the data a likely possibility.
5. In the social behavior analysis, “non-social” periods combined epochs when the test subject was alone with epochs when there was a juvenile in the context but no active interaction. It seems counterintuitive to combine these epochs as “non-social”. This analysis approach is not explained and there is no justification for its use.

Technical comments:
1. What exactly is the second familiar context? It is not sufficiently described.
2. Why is the second context considered a “dynamic context” if the animal is habituated to it for 5 days?
3. The authors need to better detail the experimental methods. Examples: how many juvenile mice were subjected to homecage, how many times were they subjected, why are more than 2 juvenile mice used?
4. Add the Y axis scale in figure 2D.

Version 1:

Reviewer comments:

Reviewer #1

(Remarks to the Author)

(Remarks on code availability)
The codes appeared no problem. I could not find a README file with enough instructions.

Reviewer #2

(Remarks to the Author)

This manuscript by Frost et al. aimed to explore whether contextual and socioemotional representations of neuronal ensembles in mPFC are segregated or intermixed. The author used calcium imaging to achieve neuronal activity in mPFC of mice engaged in social interactions or anxiety-related behavior across different environments and found that social interaction-related representations and anxiety-related representations were both largely orthogonal to contextual representations even if they were overlapped partly.

Although the author provided detailed responses to the review comments, some problems proposed by the reviewer has not been resolved and the overall quality of the article is not improved satisfactorily. In particular, the author intended to prove “Social and context representations could be orthogonal because they are composed of non-overlapping neuronal ensembles”, but the most intuitive way to prove whether the non-overlap between two neuron ensembles is to display a real-time signal recording video or image in which two ensembles of discharged neurons are separate when socializing in different environments and the these data are not analyzed thoroughly and interpreted precisely. The author used various algorithms to calculate whether the neuron cluster is overlapped which make the problem even complicated.

The problem regarding correlation analysis through simple of the average discharge of cells proposed in the last review version are not solved. If calculating similarity based on the average value of neuronal signals, it is incorrect and infeasible to use this method to explore the similarity of neuronal ensembles in different environments. Since the similarity analysis based on epoch is too rough and not precise enough, and could lose a lot of information, other factors should be involved to analyze the similarity, such as firing duration, delay, firing intensity and frequency, rather than a single average value of a neuron ensemble which only reflects the average discharge intensity of the entire ensemble. The high similarity of average signal intensity between two ensembles can not prove these two ensembles have similar encoding or activity pattern.

Technically, to prove contextual and socioemotional representations are orthogonal, these data would be needed, such as the overlap percent of contextual and socioemotional representations and the discharge pattern of neurons on the singe-cell level. However, the pan-neuronal GCaMP recording and data analysis strategy in this paper is too rough and this method can not supply the data mentioned above at all. There has been lots of research describing the neuronal encoding pattern of contextual or socioemotional representations in the mPFC with more precise and advanced methods on the single cell level. In contrast, the method of pan-neuronal GCaMP recording severely decreased the accuracy and reliability of the study’s conclusion and the novelty of the whole study.

Moreover, there are still some problems with the mouse behavioral paradigm and experimental design. The paradigm design is too complex and weird. It is redundant to set up the static group. Given that the context of epoch A and B is the same and the comparison between epoch A and B in dynamic group is totally equivalent to the comparison between A, B, C, and D in static group. The paradigm can be further simplified, such as adding two social objects into the context 1 and 2 respectively. In other words, static can be totally deleted. In the paradigm design, context 1 is homecage which brings other interference factors. For example, the difference of familiarity and safety assessment of mice with context 1 and 2 would interference the results.

The overall logic of the paper is also problematic. Figure 4-5 seem to be redundant. Figure 1-3 focus on the similarity between social and environmental representations, while Figure 4-5 shift to focus on the similarity between anxiety and environmental representations. However, these two part of the article are independent and have little connection, resulting in a logical disconnection of the entire article. In this article, exploring the similarity between anxiety and environmental representations is redundant and makes no sense.

Minor

1) As pointed out in the last review version, Fig.1 D is still complicated and difficult to understand. The size of blue bars is still inconsistent and these bars and arrows make no sense. The author did not explain what “expt day” actually mean. The picture design of the behavioral paradigm in Fig.1 D is hard to follow.

2) For fig.1 H and K, why are there more silent neurons during social interaction compared to non-social interaction? Does that mean the number of relatively active neurons (not highly active neuron) during social interaction is much lower than that during non social interaction?

3) The charts in Figure 3 A-C should be redesigned as it does not show the conclusion, and does not provide any effective information.

(Remarks on code availability)

Reviewer #3

(Remarks to the Author)
In the revised manuscript, Frost et al. have added new analyses as well as major additions to the text that clarify numerous prior concerns. The manuscript is improved but some serious concerns remain.

1. Abstract lines 33-34: “This shows how prefrontal cortex multiplexes parallel information streams by intermingling ensembles rather than transmitting information through distinct subcircuits”. Do the authors demonstrate that information is

not transmitted through distinct subcircuits? I don't think this is conclusively demonstrated and this sentence should be reworded.
2. The use of the term "dynamic context" does not seem appropriate given that the context is a slightly larger rodent cage that contains only bedding and the animals are habituated to the context. "Context shift" would be more appropriate.
3. For Figure 2C-D, Supplementary Figure 8, and Supplementary Figure 13D. Although the classifiers performed statistically better than chance, there are numerous instances where the accuracy was 56% or 54%, or similar. What types of errors are the classifiers making? What is the precision and sensitivity of the classifiers? The authors should report the F1-scores.
4. In Supplementary Figure 8, the authors report that they "observed qualitatively similar results in both cases". Although this figure is especially difficult to interpret because of the poor labeling and complete lack of panel labeling, the correlation coefficients in the left panels look much lower in what I think are the "nonsocial in the presence of a conspecific" matrix. Are they significantly different from the homecage without conspecific values?
5. Supplementary Figures 1, 2, 6, 7, and 8 need panel labeling.
6. Please report the number of mice from the social experiment that were also run through the anxiety assays.
7. From the rebuttal: "Our study is based on identifying the degree to which encoding by individual neurons is conserved across contexts – for this it is not clear why cell type specific information would be useful." It is not clear to me how this information would NOT be useful. Generalization of cell-type specific information across anxiogenic or social contexts would be of tremendous interest as it could lead to identification of genetic factors influencing abnormal social behavior or maladaptive anxiety.
8. From the rebuttal: "The aforementioned studies of anxiety-related behavior (Adhikari et al., Neuron, 2011; Padilla-Coreano et al., Neuron, 2016) record from the mPFC generally and have not found differences between encoding in the prelimibic vs. infralimbic cortices." There is a wealth of literature that supports functional differences between the PL/dorsomedial and IL/ventromedial in fear, anxiety, reward, and social behaviors. Here are a few reviews: doi.org/10.1016/bs.irn.2020.11.014; doi.org/10.1016/j.biopsych.2017.10.030; doi.org/10.1038/s41380-020-0685-9. From what the authors report, it seems that they were recording from the ventromedial prefrontal cortex. This should be explicitly stated in the abstract and throughout the manuscript.
9. From the rebuttal: "It is not standard to habituate mice to either the EZM or EPM, as mice tend not to explore these mazes and may lose their preference for the closed arms once they have been habituated." I was referring to the psychological term habituation, which would have been a potential issue if the maze exposure time was 30 minutes. My previous concern has been addressed by clarifying that the maze exposure time was 10 minutes and by the addition of the behavioral data in the supplement.
10. From the rebuttal: "We did utilize both male and female mice (5 female and 3 male mice for social experiments and equal numbers of male and female mice for the anxiety experiments. Unfortunately these experiments were performed several years ago and information which would allow us to assign genders of individual mice has been lost." It is unfortunate that the authors did not employ better record keeping.
11. "These confirm the key principle that that the prefrontal cortex multiplexes contextual vs. socio-emotional information using orthogonal but largely orthogonal representations in both male and female mice." It is not clear to me how this statement is substantiated. All mice in Rebuttal Fig 2 are male and the authors are unable to identify the sex of the mice in each data set of the manuscript. The data are similar across mice but as the authors state, they are underpowered to detect sex differences. The authors should add a caveat to their discussion regarding potential sex differences.

(Remarks on code availability)

Version 2:

Reviewer comments:

Reviewer #2

(Remarks to the Author)
The manuscript by Frost et al. aimed to use microendoscopic calcium recording to explore whether contextual and socioemotional representations of neuronal ensembles in mPFC are segregated or intermixed and whether this cause socioemotional encoding to remap or generalize across environments. The author found that social interaction-related representations and anxiety-related representations were both largely orthogonal to contextual representations even if they were overlapped partly.
Although the author provided detailed responses to the review comments, major problems proposed previously has not been resolved and the overall quality of the article is not good enough for publication.

1) The author calculated similarity based on the average value of neuronal signals to explore the similarity of neuronal ensembles in different environments. Although this method can achieve the conclusion "Neuronal ensembles representing context and social interaction overlapped more than expected by chance while nevertheless remaining largely orthogonal. Anxiety-related representations similarly generalized across environments while remaining largely orthogonal to contextual information", such similarity analysis is still too rough and not precise enough for a solid conclusion. The author should conduct more precise analysis to provide further verification.

2) The author claimed that "It was our goal not to define the activity of specified neuronal populations but to understand how the prefrontal circuit as a whole functions to encode different behaviors in parallel." Ignoring the specific pattern between single neuron or ensemble and behavior and understand how the prefrontal circuit as a whole functions to encode different

behaviors only by the average value of neuronal signals makes no sense. The average value of neuronal signals can be affected by many factors such as the virus, the fiber and the surgery accuracy, etc. And the average value of neuronal signals of social behavior is greatly decided by social frequency. The smaller social place leads to the higher social frequency which leads to higher average value, let along that average value can change greatly among mice. What keeps consistent is the single curve pattern of single specific behavior and that is critical rather than just average value. Fig.3F is too rough to provide valuable information.

3) The author used the word “orthogonal” for many times but what is the clear meaning of this word to the neuronal ensemble? The author achieved the conclusion “social interaction-related representations and anxiety-related representations were both largely orthogonal to contextual representations” by different algorithm analysis but what is the specific biological significance? My understanding of this paper is that the author found social/anxiety ensemble and contextual representations have different average signal value. If so, this conclusion is meaningless.

4) What is the onset of social interaction? Fig.1 E showed that the signal recorded for 60 s but a single social behavior can not last for 60 s. The author intended to calculate the average signal value as a whole, but as what we comments previously, it would lost many information and such kind of analysis fail to give information with sufficient value.

5) In Figure 4, the author concluded that “Context-invariant patterns of activity for anxiety-related information”. Does that mean the average signal value of vmPFC is the same among different mazes? If so, what is the meaningfulness? The mice could all be anxious among these mazes and average signal value just should be the same.

6) How did the data in Fig.1 E transform into the data in Fig.1 F and what is the calculation process to produce the cumulative distribution curve. What is the meaning and calculation method of the abscissa “frame active”. The authors did not elaborate this issue very well.

7) What does “frame” mean specifically? Such as “Bar graph showing the proportion of highly active neurons (>7.5% of frames)”. The word “frame” is hard to understand. Please use more precise biological phrases or neuroscience-related words or give a definition.

8) As pointed out before, in Fig.1 D there are some blue bars representing social interaction and there are four blue bars in each epoch. Does that mean the author would make the test mice social with partner for four times in a single epoch? Furthermore, these blue bars are of inconsistent thickness. Some blue bars are obviously more thick and the distance between blue bars is different. Does that mean the social time is different? Do thick blue bar mean more social time and the bigger distance between blues bars mean longer non-social interval? If so, the author should note it in the legend. And if not, then these elements should be deleted.

(Remarks on code availability)

Reviewer #3

(Remarks to the Author)
The revised version of the manuscript has been improved by the addition of a Supplementary Table showing classifier performance, updated figure labeling, and editing of the text. I have two remaining concerns.
1. The authors should interpret the significance of the high false negative rate in the performance of the classifier in the Train-Test Between Epoch condition.
2. My previous comment about Supp Fig. 8 was not fully addressed. I had asked whether there are significant differences between the homecage alone and the nonsocial with conspecific conditions. This is important because reviewers had concerns about pooling these periods together for analysis. If the conditions are statistically different than this should be stated and pooling the data should be justified.

(Remarks on code availability)

Open Access This Peer Review File is licensed under a Creative Commons Attribution 4.0 International License, which permits use, sharing, adaptation, distribution and reproduction in any medium or format, as long as you give appropriate credit to the original author(s) and the source, provide a link to the Creative Commons license, and indicate if changes were made.
In cases where reviewers are anonymous, credit should be given to 'Anonymous Referee' and the source.
The images or other third party material in this Peer Review File are included in the article’s Creative Commons license, unless indicated otherwise in a credit line to the material. If material is not included in the article’s Creative Commons license and your intended use is not permitted by statutory regulation or exceeds the permitted use, you will need to obtain permission directly from the copyright holder.
To view a copy of this license, visit https://creativecommons.org/licenses/by/4.0/

REVIEWER COMMENTS

Reviewer #1 (Remarks to the Author):

Frost et al. used calcium imaging to examine how prefrontal neurons encode social and context information in different environments with four novel mice. Their analyses showed that representations of two types of information are partly overlapped but almost independent. In addition, they tested representation patterns of context and anxiety-related information. These results provide crucial insights into how prefrontal cortex multiplexes parallel information to achieve socioemotional and context-invariant encoding. Overall, the experiments are well designed, and I am very enthusiastic about this manuscript. However, there are several analytical issues that need to be clarified. Especially, reaching the conclusion that "prefrontal neurons show orthogonal representations" based on their results alone seems a significant logical jump.

(Major)
(1) Overall, the authors defined two periods: social and non-social. They described "'Social interaction' was limited to timepoints when the mouse was actively interacting with the novel juvenile, whereas 'nonsocial' included both timepoints when the juvenile was present but the mouse was not engaged in interaction plus the preceding period when the mouse was alone (before the juvenile was introduced)." There is a need to verify whether this definition is appropriate.

Our Response: To address this concern, we confirmed that our findings do not depend on defining nonsocial timepoints to include both periods when the mouse was alone and those when a juvenile was present but they were not interacting. Specifically, Supplementary Figure 8 shows separate analyses which compare periods of social interaction to periods when either a juvenile was present but there was no interaction (top panels), or when no juvenile was present (bottom panels). We observed similar findings in both cases, which recapitulate our key findings that the PFC encodes both contextual and social information, and that social representations are persist across contexts.

(1-1) First, please further explain how they defined "social bouts". In the methods, they described it was scored manually, but what were more objective factors such as the distance, angle, and posture of the mice? As this definition affects all the results, it would be beneficial to have objective scoring metrics rather than subjective ones. However, even if it is unavailable, at the very least, detailed descriptions should be provided. If necessary, please include photos of typical mouse behaviors in the figures.

Our Response: Similar to many previous studies of social interaction in mice (*Frost, 2021; Brumback, 2018; Liang, 2016; Gunaydin, 2014; Murugan, 2017*), we defined social bouts by manually identifying periods in which the test mouse interacts with a conspecific, i.e. from the moment they approach and sniff the conspecific until that conspecific breaks away. We apologize that the details of this were not previously present. As is now explicitly stated in the methods "Each social bout began when the test mouse moved towards the conspecific target, with its nose oriented towards the conspecific, and its nose either made contact with or was immediately adjacent to the conspecific. Each social bout ended when the test mouse or the conspecific target moved apart such that the nose of the test mouse was no longer in contact with, or immediately adjacent to, the target." We now include relevant example images as well as quantification of social interaction by epoch in Supplementary Figure 1.

(1-2) As the authors described, the "nonsocial" periods contain two completely different timepoints: (i) when the juvenile was present but the mouse was not engaged in interaction, and (ii) the preceding period when the mouse was alone. In a strict sense, the former is social environment without social interaction, while the latter is completely nonsocial. Therefore, neuronal activity may be considerably different between the two periods. Before combining these periods (before Fig. 1F), please first provide analyses of neuronal activity to validate and claim that these activity patterns in the two periods are actually identical and could be combined. Otherwise, all analyses may need to be performed by classifying into three periods: social, when the juvenile was present without social interaction, and when it was alone.

Our Response: We are not sure what the Reviewer means by “to validate and claim that these activity patterns in the two periods are actually identical.” (It is extremely unlikely that neural activity recorded at different timepoints would ever actually be identical.) But we very much appreciate their concern that activity in these two periods might be different in ways that affect our conclusions. As noted above, we have therefore performed separate comparisons of activity during social bouts to these the two non-social periods (Supplementary figure 8) and found the same results (confirming our original conclusions) in both cases.

(2) Interpretations of overlap and orthogonalization. For example, the authors repeatedly used the term “orthogonalization” (such as in the Abstract), but based on the most important data in Figure 3, this interpretation seems insufficient.
(2-1) Figure 3E shows that, as the author described, there are no significant differences between real and shuffled datasets in the comparison of Soc AB-Context AB. However, these real data seem to have some positive similarities. To more precisely claim that they are orthogonal, in addition to this comparison, statistical tests versus 0 are necessary.

Our Response: We have performed the statistical tests requested by the Reviewer; none of the similarities between Soc AB-Context AB are significantly different from 0. That said, all of the p-values are approaching significance ($p = 0.06 – 0.11$). This underscores that for neural data it is always a bit of a judgement call whether two representations should be considered orthogonal, and this is why we originally used terms such as “near-orthogonal” or “approximately orthogonal” in the main text. We have changed the use of “orthogonal” in the Abstract to “largely orthogonal” and used similar terminology throughout the manuscript.

(2-2) Related to this, Figure 3F shows that some neurons seem to more preferentially show both social and context information (as indicated by the dense plots on the upper-right area), suggesting a possibility that these neurons encode both information more than chance levels. To further test whether there are overlapped representations, testing whether these individual proportions in Figure 3G are explained by chance levels or not by using shuffled datasets.

Our Response: This is an interesting point and we have added additional analysis and discussion regarding this point. Notably, we show that neurons do in fact have a greater than expected chance of being modulated by both behaviors; however it should be noted that neurons which are positively modulated by one behavior have a comparable chance of being positively or negatively modulated by the opposing behavior, and this permits an approximately orthogonal relationship to be maintained.

(2-3) In addition, Figure 3G showed the increases in the percentages of neurons showing modulation in the dynamic context, rather than static context as shown by green arrows and the authors concluded that “however this overlap includes neurons modulated in opposing directions, maintaining an approximately orthogonal relationship between these representations”. This is overstatement as there are no data to support that these increases in individual neuronal types are perfectly counterbalanced. Please consider to add some statistics or resume this statement.

Our Response: It is unlikely that modulations would be “perfectly counterbalanced” in neural data, but it is not necessary for the number of neurons modulated in either direction to be perfectly counterbalanced to maintain approximately orthogonal representations. As long as the neuronal ensemble modulated in one direction for one type of encoding includes sufficient positively / negatively-modulated numbers of neurons with respect to the other type of encoding, approximate counterbalancing of activity patterns can be achieved and near-orthogonality can be maintained. The point we made in the original manuscript was that each of the four ensembles (positively or negatively modulated by social or context) contains *greater-than-expected overlap* with *both* the positively and negatively-modulated ensembles associated with the other type of encoding. There are only two ways to achieve near orthogonality between representations – either you use non-overlapping neuronal ensembles to encode the different representations, or you counterbalance modulation in opposing directions for one variable within each (positively or negatively-modulated) ensemble encoding the other variable. Thus, our

finding that social / context overlap in the dynamic context is *consistently* (in all cases) greater than in the static context case (which controls for chance levels of overlap expected based on time-dependent effects) indicates that near-orthogonality is maintained via the latter strategy (counterbalancing, rather than minimizing, overlap between social and context ensembles). We have rewritten the text associated with this point on page 5-6 to hopefully make this more clear.

(2-4) According to these analyses, they claimed that these representations were "heavily" overlapping. But there seems only a minority of neurons (few %). Is it just a "partial" overlap? Similarly, the term "orthogonalization (cosine similarity=0)" may be rephrased as it may not be actually "orthogonal" but just "independent representations" (In my personal opinion, I believe it might not be necessary to force the use of the term 'orthogonalization).

Our Response: Only a few % of the *total* number of neurons are overlapping. But among neurons which are strongly modulated by one variable (e.g., social interaction) a very large fraction (~40%) are also modulated by context. The key point here, as noted above, is that representations for context and social interaction consistently overlap *significantly more than expected by chance* (based on statistical comparisons of overlap on the static context vs. dynamic context days). This confirms that these representations are not independent but rather intermingled in a statistically meaningful way.

(3) Figure 3 uses cosine similarity but Figure 5 uses "cosine similarity (-shuffled)". Why did they present the data in a different manner? As these graphs are most important to interpret orthogonalization or independency of representations compared with chancel level, please be consistent.

Our Response: We initially chose to present "cosine similarity (- shuffled)" in Fig. 5 (instead of separately plotting cosine similarity for real and shuffled data) so as to reduce the number of quantities being plotted and number of visual comparisons, in order to hopefully make the figure simpler / more digestible. However, in response to the Reviewer's comment we have reintroduced the shuffled values in F and G.

(4) Please provide more information about linear classifier. How many cells or what percentage of cells actually contributed to the classification? Was there a correspondence between the number of calcium events (baseline excitability) and the modulation index exhibited by these cells?

Our Response: We have added new plots which should address these questions. Specifically, in Supplementary Figures 6-7, we show that there is a very weak relationship between the activity of each neuron and its modulation index / classification weight. Supplementary Figure 7 shows that there is a very strong relationship between modulation index and classifier weight. As shown by the bottom panels in Supplementary Figure 7, there is a continuous distribution of classifier weights associated with individual neurons so there is no simple answer to "how many cells actually contributed to the classification," but clearly the classification is based on distributed network activity rather than activity in a small subset of neurons.

(5) Overall, the schematic illustration at the beginning should be improved, especially in Figure 1D. For example, the illustrations could be improved by showing when, where, and how many novel mice were tested in each epoch and day. The best way would be to have someone unfamiliar with this experiment look at it, ensuring they can quickly understand the experimental paradigm from this illustration.

Our Response: We have rewritten the legend for Figure 1D to present additional details and hopefully make this more explicit / clear.

(6) Were the mice tested in Figure 1-3 and Figure 4-6 similar? If so and they could monitor the same neurons across these behavioral tests, they could further obtain new insights into how social interaction-related cells are overalled with anxiety-related cells (optional).

Our Response: We agree that this would be of substantial interest and would allow us to ask corollary questions about the relationship between anxiety and social ensembles. While a subset of mice did run both assays not all were run in both, and the two assays were not run on consecutive days so we feel it would be difficult to make claims about individual neurons imaged across days.

(7) The Discussion part "it is unclear whether the context-invariance of these representations arises within the prefrontal cortex or upstream regions, or via interactions between them". Please elaborate a bit more on the potential for detailed mechanisms (e.g. brain regions? sparsity of connectivity?).

Our Response: We have added additional text in the Discussion to highlight the potential roles of distributed inputs (synapsing onto distinct populations of cells) and of local recurrent connectivity.

(Minor)
(1) Overall, why the unit of neuronal activity is frame? Please change it to Hz (or /min) as it is easier to understand in that unit.

Our Response: Because we are using Ca^{2+} imaging, it is not straightforward to directly convert our calcium signal to a spike rate. Like many other groups, we have used binarized activity rasters to differentiate periods when Ca2+ signals rise rapidly, in a manner reflective of high spiking, from quiescent periods which presumably correspond to much lower / baseline levels of spiking. Thus, the number / fraction of active frames is a natural unit / metric to use for quantifying activity. While we could in principle convert this to Hz, we feel this would be misleading as it would suggest that we are measuring something directly equivalent to spike rate.

(2) The picture in Figure 1B is poor. Please provide clearer photos. In addition, please provide typical calcium fluorescent (dfoverf) traces around Figure 1B.

Our Response: We have increased the size of the photos, and added example Ca^{2+} traces from this recording.

(3) Overall, in the Legends, please describe how many asterisks correspond to which P-values.

Our Response: Thank you, this has been added.

(4) Figure 1G: why they choose this threshold (>7.5% of frames) to define active cells? What is the rationale?

Our Response: This threshold is somewhat arbitrary, but is a somewhat 'round' number which, as shown in Figure 1F and 1I, is well past the inflection point for the cumulative distribution of activity levels, and identifies a small, but meaningful fraction of cells (~10%).

(5) Figure 3D: the two matrices of Static and Dynamic appear very similar. Are they really correct graphs?

Our Response: There are in fact subtle differences between the two panels; the similarity in this graph largely reflects the similarity between the graphs in 2H.

Reviewer #2 (Remarks to the Author):

In this manuscript the authors investigate the key role of mPFC in representing social interaction and anxiety-related avoidance across environments. The author imaged neuronal activity in the medial prefrontal cortex of mice engaged in social interactions or anxiety behaviours, and found that population-level activity within the mPFC is dynamically reorganized in a context-dependent manner, while the specific neuronal ensembles with socioemotional information are context-invariant. Moreover, they found the overlapped neuronal ensembles representing social and anxiety-related information maintain a near-orthogonal orientation relative to representations of contextual information.
Although the topic proposed is somewhat novel and potentially important, the experiments were not logically designed. In particular, the classifier and the data analysis on the socioemotional and context-encoding ensembles was ambiguous. The following concerns should be addressed before publication.

Major comments:
1. According to the logic of the project, the authors intended to see if the environment had an impact on social or anxiety like behavior, and the results they obtained were that the cluster was very stable and less susceptible to environmental influences, which may be correct. But social or anxiety involves many processes, and the responsibility or association of each step in the cluster would not be the same.

Our Response: There are many different questions that one may chose to study in an experiment like this one. It sounds like the Reviewer is interested in how different sub-processes within social interaction or anxiety-related avoidance are associated with different groups (clusters) of neurons. This is a very interesting question, but it is not the specific one we decided to pursue here. In particular, we were interested in the generalization of behavioral encoding across contexts. For this reason, we decided to focus on broad categories of behaviors (social interaction vs. non-interaction, approach to potentially anxiety-provoking regions vs. avoidance of such regions, etc.) rather than more specific behaviors that might not generalize as readily. More critically, quantitatively assessing generalization requires robust identification of activity patterns associated with specific behaviors, the use of classifiers, etc. The more you subdivide a behavior, the less samples you have for each behavioral subdivisions, and as a result, the less robust estimates for these activity patterns become, reducing our ability to accurately assess generalization. Similarly, subdividing the behavior would reduce the frames available for training classifiers. In particular, given the limited number of frames available for specific behaviors (social interaction, time spent in open arms, etc.) we do not believe we could have robustly analyzed our data if we had further subdivided these behaviors. For these reasons we chose to focus on broad categories of behaviors instead of further subdivisions.

2. It is not convincing that the authors just performed correlation analysis through simple of the average discharge of cells over a period of time. According to this method, only the average intensity of discharge (or GCaMP) and the average amplitude of brightness change can be seen. This is completely not the case with cluster coding, as the latter needs to be synchronized or sequentially distributed over time. More feasible evidence or literature should be provided to support the reliability of this method.

Our response: We are well versed in this issue as we are the only ones (as far as we know) who have actually demonstrated that synchronous co-activity between neurons adds information about social interaction over and above the information that is transmitted by the activity levels of individual neurons (Frost et al., *PLoS Biology*, 2021). In that paper, we quantified information transmitted by activity levels as well as by synchronous co-activity between neurons. While synchronous co-activity did convey additional information (which was an important finding), the bulk of information was transmitted by activity levels alone. So it is in fact reasonable to ask how much of this information, transmitted by neurons' average activity levels, is conserved across contexts. In particular, we are interested in whether the same set of neurons encode contextual information vs. social or anxiety-related signals, and the most direct way to address this is by quantifying the variation in neurons' activity levels, as we have done.

Perhaps the most compelling argument supporting our approach is that using this approach did in fact reveal reliable and consistent representations of social information across multiple epochs separated by time.

3. For population activity for social interaction and nonsocial activity, putting social and emotional together may create clusters of interacting neurons, but it is incomprehensible to compare the similarities of several mazes (elevated zero maze, elevated plus maze, and T-shaped maze) later on.

Our Response: Both sections of the paper measure neural activity during a given behavior (social interaction in part 1 or anxiety-related approach/avoidance in part 2), occurring across multiple contexts (different environments in part 1, or different mazes in part 2). In both cases, we find major context-driven changes in overall PFC activity (c.f., Figs. 1M and 4E). In particular, in Fig. 4D-E, contextual remapping is inferred from the dissimilarity between overall activity patterns in the HC vs. those observed within the other mazes (green or red bars vs blue bars). However despite these maze-dependent changes, open arm activity patterns from one maze are significantly more similar to open arm activity patterns than closed arm activity patterns in the other maze (and vice-versa; Fig. 4G). We are not sure what the Reviewer means by saying this is 'incomprehensible' – the question of whether portions of different mazes which are presumed to elicit similar anxiety-related states are also associated with similar neural representations seems very straightforward and is indeed that basis for other prominent studies, e.g., Grundemann et al., Science, 2019.

The significance of the differences in similarity obtained by the intermediate statistical methods is not clearly explained.

Our response: If the Reviewer can identify a specific figure panel, etc. that is confusing, we would be happy to provide additional explanation.

Minor

1. The manuscript wrote that “We performed optical GCaMP measurements of prefrontal circuit activity (Fig. 1A-C) in 8 mice”. I think the author just explore the role of mPFC and didn’t explore the role of circuit, so “circuit” is not accurate.

Our response: We have changed this to ‘multineuron patterns of prefrontal activity’ to emphasize the local nature of these recordings.

2. It’s hard to understand Fig 1D according to text. The text and annotations are unclear and lack details. Why the size of blue bars is inconsistent? What do context1 and 2 refer to respectively? What does “expt day” mean?

Our Response: This has been made clearer (e.g., blue bars are now more consistent). Additional clarification is presented in the legend.

3. In Fig. 1G,1H,1J,and 1K, the percentage of active neurons during social interaction is significantly more than the percentage of active neurons during non-social stages and it is easy to understand. But why is the percentage of inactive neurons during social interaction also significantly more than the percentage of inactive neurons during non-social stages？

Our Response: This is a great question. First is it important to note that what the Reviewer calls ‘inactivity’ is not just the absence of an increase in activity – rather it corresponds to a significant decrease in activity. We and others have previously reported that neurons may be positively or negatively modulated during social interactions (c.f., Liang et al., *Neuron*, 2018; Frost et al., *PLoS Biology*, 2021). However in our previous study we did not observe negative modulation to the same degree observed here. There are a number of methodological differences; most notably whereas Frost et al. 2021 utilized 4 sequential interactions in the home cage, the latter interactions used familiar mice (the same mice as the first two interactions), and thus did not elicit as much activity modulation within the mPFC.

4. The abbreviation should be explained in the figure annotation, such as the “C1””C2” in the Fig. 1M.

Our Response: Further clarification has been added to the legend.

5. Does diagonal blocks from top left to bottom right in Fig.1M represent the similarity value of 1? Why use a dark blue block with a similarity value of 0 to represent it? I have similar question in Fig. 4D.

Our Response: Thank you for pointing this out - we have altered all such graphs so that the diagonal uses a color not included in the look up table.

6. There are many typos that need to be corrected.
(1) Page 2: “Each mouse was tested during interactions interacted with novel sex-matched juvenile conspecifics on two days.” in which the word “interacted” should be deleted.
(2) Page 26: “… indicates a model trained to distingish frames corresponding”, “distingish” should be “distinguish”
(3) Page 23: “Ca2+ activity was recorded throughout a session in which mice explored the elevated zero maze

(EZM), elevated plus maze (EPM), and a T-shaped mazel)", "mazel" should be "maze".
(4) Page 17: "Bar graph depecting the mean similarity (correlation)", "depecting" should be "depicting"

Our Response: Thank you for pointing these out – they have been remedied.

Reviewer #3 (Remarks to the Author):

The manuscript by Frost et al. entitled 'Context-invariant socioemotional encoding by prefrontal ensembles' seeks to evaluate the role of neuronal ensembles in the medial prefrontal cortex (mPFC) in encoding socioemotional information across different environments. Using deep brain calcium imaging, the authors imaged neuronal activity in the medial prefrontal cortex of mice when they were engaged in social interactions or anxiety-related avoidance within different environments. Their rigorous computational analysis shows that there are overlapping neuronal ensembles that encode context invariant social interaction and anxiety-related representations.

While the premise of this study is interesting, the experimental design is problematic, the behavior of the subjects is not presented or discussed sufficiently, and the main results lack novelty. Moreover, the manuscript is written in very technical language, which is hard to follow, and there is no detailed interpretation of the results. My main comments are below, and they are followed by more technical comments.

Main Comments:
1. There is a wealth of literature describing the neuronal encoding of social behaviors by the mPFC, and a rich literature describing the neuronal encoding of avoidance behavior by the mPFC. The extant literature includes detailed descriptions of the mPFC cell types, circuits, and pathways important for various aspects of social behavior and anxiety-like behavior, whereas the manuscript under review uses a pan-neuronal GCaMP recording strategy and only superficially reports behavior. This severely detracts from the novelty of the study.

Our Response: We agree that the literature describes a wealth of information regarding the role of the mPFC in encoding and regulating both social and anxiety-related behaviors. We ourselves have published on the role of specific cell types in social (Brumback et al., *Molecular Psychiatry*, 2018) and anxiety related behavior (Lee et al., *Neuron*, 2019). It is also true that many highly influential studies have recorded nonspecifically from prefrontal neurons to study both social (Murugan et al., *Cell*, 2017; Liang et al., *Neuron*, 2018 Levy et al., *Nat Neurosci*, 2019; Kingsbury et al., *Cell*, 2019; Kingsbury et al., *Neuron*, 2020; Zhang et al., *Nature*, 2024) and anxiety-related behavior (Adhikari et al., *Neuron*, 2011; Padilla-Coreano et al., *Neuron*, 2016). To our knowledge, the number of studies which have examined population-level activity with individual neuron resolution and cell type specificity is actually very low.

Our study is based on identifying the degree to which encoding by individual neurons is conserved across contexts – for this it is not clear why cell type specific information would be useful. We agree that recording from identified cell types is inherently more 'novel' but are not clear on why it is better for answering the particular question we are interested in. In particular, because typically only 1-2 cell types are resolved in a given study, it is not clear that this would be a useful approach for answering the general question of how the prefrontal cortex encodes information across contexts.

Furthermore, we were interested in the generalization of behavioral encoding across contexts. For this reason, we decided to focus on broad categories of behaviors (social interaction vs. non-interaction, approach to potentially anxiety-provoking regions vs. avoidance of such regions, etc.) rather than more specific behaviors that might not generalize as readily. More critically, quantitatively assessing generalization requires robust identification of activity patterns associated with specific behaviors, the use of classifiers, etc. The more you subdivide a behavior, the less samples you have for each behavioral subdivisions, and as a result, the less robust estimates for these activity patterns become, reducing our ability to accurately assess generalization. Similarly, subdividing the behavior would reduce the frames available for training classifiers. In particular, given the limited number of frames available for specific behaviors (social interaction, time spent in open arms, etc.) we do not believe we could have robustly analyzed our data if we had further subdivided these behaviors. For these reasons we chose to focus on broad categories of behaviors instead of further subdivisions.

2. The mPFC is an extensively studied region, which can be further subdivided into anterior cingulate cortex (ACC), prelimbic (PL) and infralimbic (IL) subdivisions, amongst others. The authors did not clarify from which

region they are recording. This is important because these regions are known to differentially regulate the behavior of animals.

Our Response: We have clarified in the methods that our GRIN lenses were placed near the dorsal border of the infralimbic cortex which encodes both social information and anxiety-related behavior. The aforementioned studies of anxiety-related behavior (Adhikari et al., *Neuron*, 2011; Padilla-Coreano et al., *Neuron*, 2016) record from the mPFC generally and have not found differences between encoding in the prelimibic vs. infralimbic cortices. The coordinates we used for our GRIN lens (1.7mm anterior, 0.3mm lateral, 2.1mm depth) are similar to those used by other studies that have used GCaMP imaging in prefrontal cortex, e.g., Murugan et al.: 1.8mm anterior, 0.3mm lateral, 1.9-2.0mm depth; Zhang et al.: 1.7mm anterior, 0.25mm lateral, 1.8mm depth.

3. The anxiety-like behavior test design is busy and seems overdone. There are 6 home cage recordings and 3 mazes in a day, which could be stressful to the mice and affect behaviors. Further, each test is 30 minutes long and there is no acknowledgement of within- or between-test habituation. In fact, there is no behavioral data presented.

Our Response: We have added behavioral data (Supplementary Figure 9) which confirms that mice prefer the closed arms / avoid the open arms in both the EPM and EZM. Thus, our paradigm does not appear to alter the well-known behavioral preferences of mice in these mazes. It is not standard to habituate mice to either the EZM or EPM, as mice tend not to explore these mazes and may lose their preference for the closed arms once they have been habituated. Mice had however been extensively habituated to the miniscope prior to testing. A priori, it is hard to say how much recording is too much and would be stressful for mice. I.e., is there any reason we should not assume that 5 min of recording would be stressful? We do note that many other studies have used a similar design, e.g., Adhikari et al. (2011) recorded while mice explored two different EPM-like mazes with a 1 hour rest period in between, and Muruhan et al. (2017) imaged some mice during four consecutive sessions in a linear arena (each of which was 10 min long). We should note that each recording session within a maze lasted 10 min (not 30 min) – this is now noted in the revised manuscript.

4. The authors did not report the number of each sex they recorded from, and there is no mention of whether the data differ by sex. In mice, there are very strong sex differences in both social and anxiety-related behaviors, thereby making sex differences in the data a likely possibility.

Our Response: It is certainly true that sex can influence both social and anxiety-related behaviors. However the question of whether the

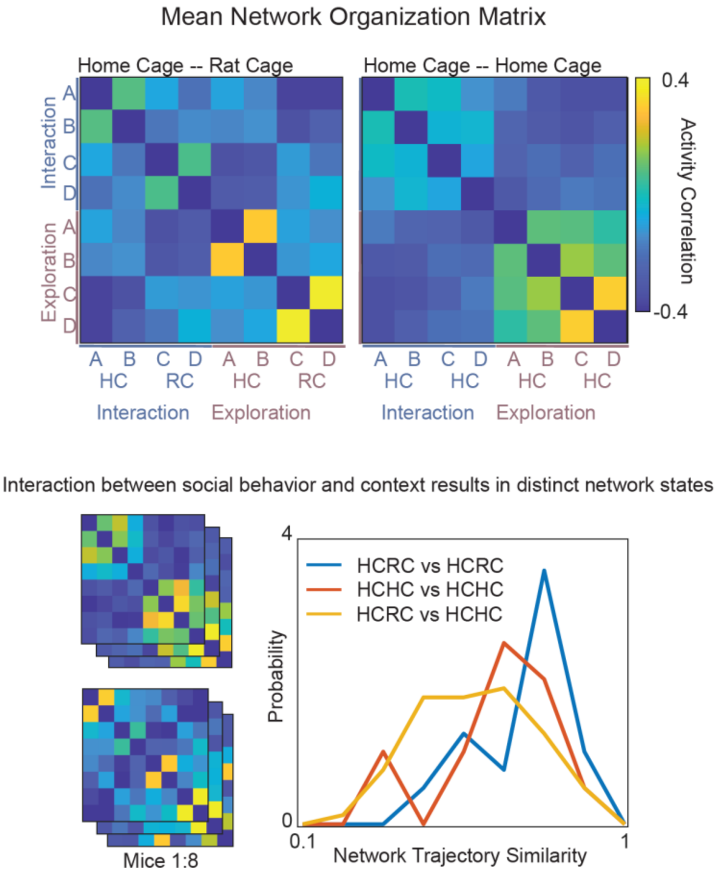

HCRC	median 0.73
HCHC	median 0.64
HCRCvHCHC	median 0.55

KS test	HCRC vs HCHC -	p = 0.016
	HCRC vs HCRCvHCHC	p = 0.0000003
	HCHC vs HCRCvHCHC	p = 0.017

Figure 1: Similar network trajectories across individual mice. We examined the degree to which prefrontal networks from individual mice followed similar trajectories of reorganization during static and dynamic experiments (top). We generated similarity matrices for each mouse during static and dynamic experiments (bottom left). We then examined the similarity between resulting matrices using Pearsons's pairwise correlations. We observed the greatest degree of similarity between dynamic context similarity matrices (followed by static experiment day), and the lowest similarity when comparing static and dynamic similarity matrices for each mouse.

prefrontal cortex multiplexes contextual vs. socio-emotional information as a general principle is a different question. We did utilize both male and female mice (5 female and 3 male mice for social experiments and equal numbers of male and female mice for the anxiety experiments. Unfortunately these experiments were performed several years ago and information which would allow us to assign genders of individual mice has been lost. Because the total number of mice used in these experiments is relatively small we would likely be underpowered to perform quantitative comparisons to assess for between-sex differences in either case.

We have examined the degree to which these findings generalize. We first examined our dataset to see if individual mice or groups of mice might encode social and contextual information differently. To do this we performed analysis of correlation plots (such as those in 1M) and these plots look very similar when plotted for individual mice. This can be seen in Fig. 1N for example – consistently across mice, population activity vectors during social interaction are more similar in the static vs. dynamic context. In support of this, we examined similarity matrices from each mouse and compared them to similarity matrices collected for both static and dynamic experiments. We predicted that if mice multiplex social and contextual information in a similar manner, similarity matrices collected on the same experiment day (ie static or dynamic) should be more like similarity matrices collected on the same experiment day than similarity matrices collected on the opposing day. This was in fact the observed result and suggests that our central finding (that social and contextual information are multiplexed in the mPFC) generalizes across the mice used in this study.

We have also examined data from ongoing experiments in our laboratory which demonstrate qualitatively similar findings in 7 male WT mice. These mice were control animals to two different experimental groups and therefore consisted of mice of two different ages, both of which were distinct from those used in these studies. While technical differences in analysis (both in terms of how the behavior was scored and how calcium data was segmented and analyzed) as well as ages of the mice preclude direct quantitative comparisons with the data presented in this manuscript (See Modulation Index Similarity Plot for these mice which corresponds to Figure 2G), they demonstrate that our findings qualitatively generalize across an independent dataset consisting of male mice and of mice of different ages. These confirm the key principle that that the prefrontal cortex multiplexes contextual vs. socio-emotional information using orthogonal but largely orthogonal representations in both male and female mice.

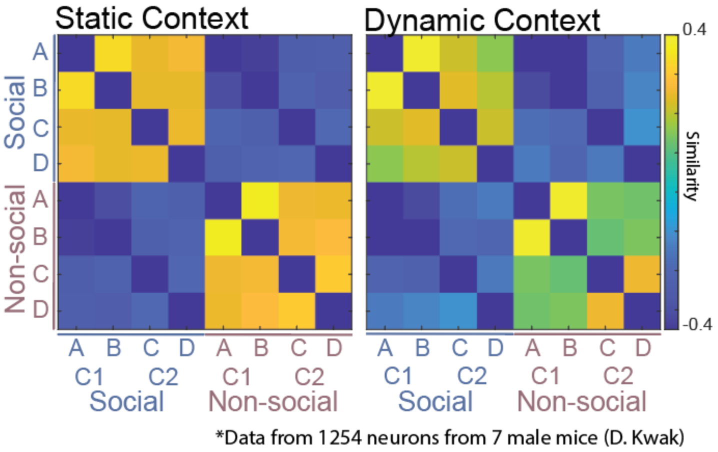

Figure 2: Preserved context invariant social encoding in 7 male mice of different ages. Here we calculated the epoch-specific modulation index to demonstrate that network representations of social information are context-independent in an independent cohort of 7 male mice. These were from mice of different ages and were Wildtype controls from experiments examining network re-organization in response to social interaction/context in disease models (credit D. Kwak).

5. In the social behavior analysis, "non-social" periods combined epochs when the test subject was alone with epochs when there was a juvenile in the context but no active interaction. It seems counterintuitive to combine these epochs as "non-social". This analysis approach is not explained and there is no justification for its use.

<u>Our Response</u>: We now performed separate comparisons of activity during social bouts to these the two non-social periods (subject alone or conspecific present but no interaction occurring). The results are shown in Supplementary figure 8 – we found the same results (confirming our original conclusions) in both cases.

Technical comments:
1. What exactly is the second familiar context? It is not sufficiently described.

<u>Our Response</u>: The second context is a larger rodent cage which measures 10.5x14 inches (roughly twice the size of the home cage) and filled with fresh bedding prior to habituation over a 5 day period. Each

mouse is habituated to a different cage so that there are no unfamiliar rodent odors. This is now explained in the Methods.

2. Why is the second context considered a “dynamic context” if the animal is habituated to it for 5 days?

Our Response: We consider the *experiment* (not the context) to include ‘dynamic context’ because the context changes (ie from context 1- 2), whereas in the ‘static context’ experiment the mice remain in context 1 (their home cage) for the entire duration of the experiment.

3. The authors need to better detail the experimental methods. Examples: how many juvenile mice were subjected to homecage, how many times were they subjected, why are more than 2 juvenile mice used?

Our Response: This information has been added to the methods under ‘social behavior.’

4. Add the Y axis scale in figure 2D.

Our Response: Thank you for pointing this out, it has been added.

Reviewer #1 (Remarks on code availability):

The codes appeared no problem. I could not find a README file with enough instructions.

Our Response: We thank the Reviewer for pointing out this omission. We have added a README file to the Github which also includes link to the zenodo repository.

Reviewer #2 (Remarks to the Author):

This manuscript by Frost et al. aimed to explore whether contextual and socioemotional representations of neuronal ensembles in mPFC are segregated or intermixed. The author used calcium imaging to achieve neuronal activity in mPFC of mice engaged in social interactions or anxiety-related behavior across different environments and found that social interaction-related representations and anxiety-related representations were both largely orthogonal to contextual representations even if they were overlapped partly.

Although the author provided detailed responses to the review comments, some problems proposed by the reviewer has not been resolved and the overall quality of the article is not improved satisfactorily. In particular, the author intended to prove "Social and context representations could be orthogonal because they are composed of non-overlapping neuronal ensembles", but the most intuitive way to prove whether the non-overlap between two neuron ensembles is to display a real-time signal recording video or image in which two ensembles of discharged neurons are separate when socializing in different environments and these data are not analyzed thoroughly and interpreted precisely. The author used various algorithms to calculate whether the neuron cluster is overlapped which make the problem even complicated.

Our Response: We thank the reviewer for their comments. This is indeed an important point. When we started these experiments we imagined that there were many potential mechanisms through which ensembles could encode different types of information (such as information related to social interactions vs. context, or information related to anxiety states vs. context). For example, it is possible that one set of neurons (set A) encodes one type of information, while another set of neurons (set B) encodes a different type of information, such that there is no overlap at all between sets A and B. This would indeed be the most conceptually straightforward way to encoding multiple types of information in parallel. If this was the case, then one could "display a real-time signal recording video or image in which two ensembles of discharged neurons are separate" as the Reviewer suggests. However, as we explicitly state many time this is NOT the case, e.g., the Abstract states "Neuronal ensembles representing context and social interaction overlapped more than expected by chance." Furthermore, it is not clear to us how simply by displaying a real-time video or image one could assess whether two ensembles associated with different behaviors overlap more or less than expected by chance. This is exactly the sort of question that requires the use of "various algorithms" which we admit is complicated but is necessary to answer the specific questions we laid out.

An alternative to the scenario in which distinct groups of neurons encode different types of information, is that these two types of information might be encoded by overlapping populations of neurons. In this case, we might find that ensembles encoding A and B might interact linearly or nonlinearly. Taking the example of social encoding one could imagine that we might have found in the latter case that social information was encoded in a context-dependent manner. In this case we would not have been able to identify persistent representations of social information across context. Instead, by using multiple different analytical methods (correlation, cosine similarity, and classifier performance), we confirmed that there are persistent representations of social and anxiety-related information despite dramatic context-dependent changes in mPFC network activity.

To aid in the interpretation of these studies, we have added additional text to the introduction and an additional explanatory figure (Figure 6) which demonstrates how orthogonal ensembles for two different behaviors may combine linearly.

The problem regarding correlation analysis through simple of the average discharge of cells proposed in the last review version are not solved. If calculating similarity based on the average value of neuronal signals, it is incorrect and infeasible to use this method to explore the

similarity of neuronal ensembles in different environments. Since the similarity analysis based on epoch is too rough and not precise enough, and could lose a lot of information, other factors should be involved to analyze the similarity, such as firing duration, delay, firing intensity and frequency, rather than a single average value of a neuron ensemble which only reflects the average discharge intensity of the entire ensemble. The high similarity of average signal intensity between two ensembles can not prove these two ensembles have similar encoding or activity pattern.

Our response: The Reviewer is correct that for some of our analysis we utilize the average activity of each neuron over a number of frames during which a mouse is either interacting or not interacting during each epoch. We then calculate the correlation coefficient or cosine similarity to determine the similarity of mean activity during a given behavior within or between environments. The inclusion of a static experiment serves as a control to examine the degree to which activity would change as a function of time if a mouse was not introduced to a new environment. Critically, we observe that population activity underlying social interaction is similar across 4 epochs in the static experiment, but it changes in a context-dependent manner in the dynamic context experiment (See Figure 1M, 1N, 1O). Furthermore, despite these context-dependent changes in activity, we observe that neurons are modulated in a persistent fashion by social interaction, regardless of context (Figure 2).

While both of these types of analysis rely on the average activity across many frames and ignore moment to moment fluctuations in activity which indeed may underlie different types of behaviors, these analysis do in fact demonstrate that similar populations of neurons reliably become active during repeated episodes of social interaction, and that 'context' encoding neurons become reliably active during multiple epochs within the same environment. We observe similar findings in the EPM or EZM (Figure 4F, G), in which population vectors in one open arm are more like the activity in the other open arm than the activity in closed portions of the maze. Thus, these analysis demonstrate that we can utilize this approach to identify neurons whose activity is modulated by socioemotional or contextual information.

Notably, we include a further classifier-based analysis in which we train a linear classifier to predict the behavior or location of a mouse based on the calcium activity within individual frames of calcium activity. This approach by its nature works on a frame-by-frame basis and therefor has higher temporal precision (a single frame is 50 ms) and would thus capture the faster timescale information the Reviewer is wondering about. Using this approach we first demonstrate that a linear classifier can utilize single frame calcium activity to predict whether the mouse is engaged in social interaction (Figure 2B, Supplementary Figure 5) , meaning that similar patterns of activity must be repeatedly recruited during social interaction which differ from those recruited during individual frames in which the mouse is not interacting. Moreover, we show that we can utilize a classifier trained in one epoch to predict activity within a different epoch (Figure 2C) – demonstrating conclusively that activity recruited during social interaction in one context is distinct from that recruited when the mouse is not interacting, and that these same patterns of activity may be persistently recruited across different epochs of behavior which occur in different contexts.

The fact that we reach the same conclusion using a frame-by-frame analysis (classifier) as we do using an averaged activity analysis (correlation between population activity vectors) thus addresses the Reviewer's concern. We apologize if the nature of the classifier analysis was not originally clear to the Reviewer.

Technically, to prove contextual and socioemotional representations are orthogonal, these data would be needed, such as the overlap percent of contextual and socioemotional representations and the discharge pattern of neurons on the singe-cell level. However, the pan-neuronal GCaMP recording and data analysis strategy in this paper is too rough and this method can not

supply the data mentioned above at all. There has been lots of research describing the neuronal encoding pattern of contextual or socioemotional representations in the mPFC with more precise and advanced methods on the single cell level. In contrast, the method of pan-neuronal GCaMP recording severely decreased the accuracy and reliability of the study's conclusion and the novelty of the whole study.

Our response: We agree with the Reviewer that "Technically, to prove contextual and socioemotional representations are orthogonal, these data would be needed, such as the overlap percent of contextual and socioemotional representations and the discharge pattern of neurons on the singe-cell level." Indeed, we provided exactly this information in Figure 3F-G. Figure 3F shows the discharge pattern of each single cell relates to both social interaction and context. Figure 3G lists the overlap percent of contextual and socioemotional representations – exactly the information the Reviewer seems to have wanted.

The key finding is that the fraction of neurons which are modulated by both types of information is quite high, but the direction in which a neuron is modulated by context cannot be predicted by the direction it is modulated by social interaction. This can clearly be visualized in Figure 3G, which demonstrates the population of positively modulated social neurons contains subpopulations which are either positively or negatively modulated by context. Critically, the overlap between positively or negatively modulated social neurons, and neurons which are positively or negatively modulated by context is significantly greater (based on chi-squared tests) in the dynamic context compared to the static context, showing that this overlap is indeed related to context, not time or some other factor. (We observe a similar finding when we examine the neurons positively modulated by context). We apologize if these figures and the information they provide were not previously clear to the Reviewer.

The Reviewer makes the point that subsets of neurons identified either genetically or anatomically (i.e. based on projection target) might overlap or encode these behaviors in different ways (or even not respond to certain behaviors). It was our goal not to define the activity of specified neuronal populations but to understand how the prefrontal circuit as a whole functions to encode different behaviors in parallel. Even if we were to perform further experiments targeting many different projection or otherwise labeled populations one at a time and were to find that some of these populations encoded two types of information using nonoverlapping populations, the fact would remain that some subpopulations must encode these two behaviors using overlapping, orthogonal representations, as we have demonstrated that more individual cells are significantly modulated by social interaction and context than expected by chance (by comparing the dynamic context experiment to the static context experiment).

Moreover, there are still some problems with the mouse behavioral paradigm and experimental design. The paradigm design is too complex and weird. It is redundant to set up the static group. Given that the context of epoch A and B is the same and the comparison between epoch A and B in dynamic group is totally equivalent to the comparison between A, B, C, and D in static group. The paradigm can be further simplified, such as adding two social objects into the context 1 and 2 respectively. In other words, static can be totally deleted. In the paradigm design, context 1 is homecage which brings other interference factors. For example, the difference of familiarity and safety assessment of mice with context 1 and 2 would interference the results.

Our response: Our principle goal was to identify prefrontal representations of both social information and contextual information, and then determine the relationship between these two different representations. It should be noted that each mouse was habituated over multiple days to the second context so that it is not to be construed as a anxiety-provoking or unfamiliar setting, rather just a different setting then the home cage.

The Reviewer seems to suggest that we could perform the experiment in a single day by having two social epochs in each of the two contexts. This is in fact exactly what we did on the dynamic context day; this provides the ability to examine the similarity between social encoding within vs. between context. Using this paradigm we demonstrate that there is a dramatic context-specific reorganization of mPFC activity, and yet, despite this shift, we observe persistent representations of social information. The static experiment day then exists as a control to demonstrate conclusively that when there is not a shift in context, social representations are relatively stable and similar to one another, implying that the shift in network activity we observed in the dynamic context was related specifically to a change in the environment and not driven by another factor, e.g., the passage of time.

The overall logic of the paper is also problematic. Figure 4-5 seem to be redundant. Figure 1-3 focus on the similarity between social and environmental representations, while Figure 4-5 shift to focus on the similarity between anxiety and environmental representations. However, these two part of the article are independent and have little connection, resulting in a logical disconnection of the entire article. In this article, exploring the similarity between anxiety and environmental representations is redundant and makes no sense.

Our Response: The Reviewer is correct that “Figure 1-3 focus on the similarity between social and environmental representations, while Figure 4-5 shift to focus on the similarity between anxiety and environmental representations.” Our central goal was to identify co-existing socioemotional and context representations, then determine how the relationship between them. First we did this for representations of social interactions and context, then we verified that the principles we uncovered were true for representations of anxiety-related information and context. In both cases, we observe that socioemotional representations are context-invariant, providing evidence that our finding is not behavior-specific but rather generalizes across multiple types of information, thereby permitting the mPFC to regulate behavior in a flexible manner. We have added text in the results to further flesh out the reasoning behind this transition: “These findings might be specific for particular types of behavior (e.g., social interaction) or alternatively, might generalize across different behaviors. We therefore tested whether these general principles (orthogonal representations for different types of information enabling context-invariance) extend to a distinct prefrontal-dependent behavior, namely anxiety-related avoidance.”

Minor

1) As pointed out in the last review version, Fig.1 D is still complicated and difficult to understand. The size of blue bars is still inconsistent and these bars and arrows make no sense. The author did not explain what “expt day” actually mean. The picture design of the behavioral paradigm in Fig.1 D is hard to follow.

Our response: We have added different colored mice to emphasize that each epoch is defined by interactions with a distinct, novel, juvenile mouse. We have changed the narrative prompts to ‘Start in Context 1, and either Stay in Context 1 (Static Context Day) or Move to Context 2 (Dynamic Context Day). We have also added further clarifying text to the legend: “Ca^{2+} activity was visualized during serial interactions between one test mouse and 4 distinct novel juvenile mice on two experimental days. On the day of the static context experiment, serial interactions occurred within a single environment (the home cage of the test mouse). To examine mPFC activity during social interactions occuring in different contexts we performed a dynamic context experiment on a separate day. During the dynamic context experiment, mice

were imaged during interactions in their home cage, then moved to a distinct, familiar environment where they were imaged during interactions with two additional mice.”

For consistency we also updated this schematic in Fig 2,3 as well as Supp Fig 3, 5 & 8.

2) For fig.1 H and K, why are there more silent neurons during social interaction compared to non-social interaction? Does that mean the number of relatively active neurons (not highly active neuron) during social interaction is much lower than that during non social interaction?

Our response: This is indeed the case and can be visualized in Figure 1F and 1I. Note: we observed a similar finding in Frost et al., PLoS Biol, 2021 – many neurons significantly decrease their activity during social interaction (c.f., Fig. 1D and 6C).

3) The charts in Figure 3 A-C should be redesigned as it does not show the conclusion, and does not provide any effective information.

Our Response: Figure 3 has been re-designed to more effectively convey the goal of the analysis (to identify the ensembles associated with specific behaviors/types of information). We added the title “Ensemble Generation by Information Type” and added labels conveying the type of ensembles being generated within the figure.

Reviewer #3 (Remarks to the Author):

In the revised manuscript, Frost et al. have added new analyses as well as major additions to the text that clarify numerous prior concerns. The manuscript is improved but some serious concerns remain.

1. Abstract lines 33-34: "This shows how prefrontal cortex multiplexes parallel information streams by intermingling ensembles rather than transmitting information through distinct subcircuits". Do the authors demonstrate that information is not transmitted through distinct subcircuits? I don't think this is conclusively demonstrated and this sentence should be reworded.

Our response: The Reviewer is correct, the wording here is somewhat ambiguous and does not convey what we found with the proper specificity. While we demonstrate that a substantial proportion of neurons are modulated by both behaviors there is also a population of neurons which are modulated only by one or the other in our experiments. We have changed the wording in the abstract to 'using overlapping ensembles rather than transmitting information through largely distinct populations.'

2. The use of the term "dynamic context" does not seem appropriate given that the context is a slightly larger rodent cage that contains only bedding and the animals are habituated to the context. "Context shift" would be more appropriate.

Our response: At the end of the day, we are willing to make this change if the Reviewer insists. But the point is we did one experiment in which the context did not change ('static context') and another in which it did change, hence 'dynamic context'. We think the exact nature of the dynamic context is quite clear from the text and figures, and that this is an issue of preferred wording rather than a substantive scientific issue. But again, if the Reviewer feels that the current wording is scientifically problematic, we will change it as requested.

3. For Figure 2C-D, Supplementary Figure 8, and Supplementary Figure 13D. Although the classifiers performed statistically better than chance, there are numerous instances where the accuracy was 56% or 54%, or similar. What types of errors are the classifiers making? What is the precision and sensitivity of the classifiers? The authors should report the F1-scores.

Our response: The reviewer is correct that the classifier performs marginally above chance when trained on one epoch and tested on another. To examine the types of errors being made we calculated F1, Recall and Precision for classifiers trained to identify social interaction frames (See Supplementary Table 1). The classifier statistics aligned with the performance of the classifier and we observed the highest F1, Precision, and Recall when classifier was trained and tested on all frames from the experiment (top). The performance dropped somewhat when the classifier was trained and tested only within a single epoch (middle shows data from Epoch A only and data averaged across epochs A, B, C and D; n = 8 mice). We next examined errors made by the classifier when trained on frames from one epoch and tested on frames from other epochs. Here precision was generally higher than 0.5 whereas recall was less than 0.5 suggesting that frames predicted to be social were generally accurate but that a substantial number of frames were missed (n = 8, mean is displayed).

4. In Supplementary Figure 8, the authors report that they "observed qualitatively similar results in both cases". Although this figure is especially difficult to interpret because of the poor labeling and complete lack of panel labeling, the correlation coefficients in the left panels look much lower in what I think are the "nonsocial in the presence of a conspecific" matrix. Are they significantly different from the homecage without conspecific values?

Our response: We apologize that the original figure was not more clear. We have added labels and headings. The Reviewer is correct that in the static context there is less similarity between non-social periods in different epochs compared to when we use either all nonsocial frames, or only nonsocial frames during which the mouse is alone. While there are a number of potential reasons, the more critical analysis is that of the dynamic context which shows a checkerboard pattern consistent with context-dependent changes in activity underlying both social and nonsocial epochs. Critically, when we subsequently shuffle data to examine within-epoch modulation during social interaction, we still observe stable representations of both social and non-social periods. Despite this qualitative similarity the correlation coefficients are again somewhat lower, and this most likely stems from the limited number of frames corresponding to non-social behavior in this analysis.

5. Supplementary Figures 1, 2, 6, 7, and 8 need panel labeling.

Our response: Labels have been added to all figures as requested.

6. Please report the number of mice from the social experiment that were also run through the anxiety assays.

Our response: 6 of the mice were run in both experiments – this is now stated in the methods.

7. From the rebuttal: "Our study is based on identifying the degree to which encoding by individual neurons is conserved across contexts – for this it is not clear why cell type specific information would be useful." It is not clear to me how this information would NOT be useful. Generalization of cell-type specific information across anxiogenic or social contexts would be of tremendous interest as it could lead to identification of genetic factors influencing abnormal social behavior or maladaptive anxiety.

Our response: Of course cell type information would be useful – the question is just would it be necessary for answering the specific question we set out to answer? Our central finding is that highly overlapping populations of prefrontal neurons are utilized to encode different behaviors simultaneously. And we are not trying to identify the role of a specific pathway in a specific behavior, for which it would make sense to look at a specific cell type. Rather, we are just trying to understand basic computational principles that apply to the PFC network. For this, it is useful to look at the entire population of PFC neurons. Of course, one might find interesting details by looking at specific cell populations, but if one had started out by doing this, one might not have gotten an accurate picture of how the PFC as a whole operates. E.g., if we were in fact to perform myriad experiments examining different subpopulations of neurons, we might identify some subpopulations which encode one behavior but not another. But based on what we have observed, some other (potentially unsampled) subpopulations must encode both two behaviors using overlapping, and orthogonal representations. To put it simply, to answer basic questions about population-level encoding, there is value in starting off by recording from as many neurons / as broad of a population as possible.

8. From the rebuttal: "The aforementioned studies of anxiety-related behavior (Adhikari et al., Neuron, 2011; Padilla-Coreano et al., Neuron, 2016) record from the mPFC generally and have not found differences between encoding in the prelimibic vs. infralimbic cortices." There is a wealth of literature that supports functional differences between the PL/dorsomedial and IL/ventromedial in fear, anxiety, reward, and social behaviors. Here are a few reviews: doi.org/10.1016/bs.irn.2020.11.014; doi.org/10.1016/j.biopsych.2017.10.030; doi.org/10.1038/s41380-020-0685-9. From what the authors report, it seems that they were recording from the ventromedial prefrontal cortex. This should be explicitly stated in the abstract and throughout the manuscript.

Our response: As requested, we have made clear that our recordings are from vmPFC.

9. From the rebuttal: "It is not standard to habituate mice to either the EZM or EPM, as mice tend not to explore these mazes and may lose their preference for the closed arms once they have been habituated." I was referring to the psychological term habituation, which would have been a potential issue if the maze exposure time was 30 minutes. My previous concern has been addressed by clarifying that the maze exposure time was 10 minutes and by the addition of the behavioral data in the supplement.

Our response: We thank the reviewer.

10. From the rebuttal: "We did utilize both male and female mice (5 female and 3 male mice for social experiments and equal numbers of male and female mice for the anxiety experiments. Unfortunately these experiments were performed several years ago and information which would allow us to assign genders of individual mice has been lost." It is unfortunate that the authors did not employ better record keeping.

Our response: We agree. To provide some context, the first author had stored this information along with surgery records in the senior author's lab space when he left to start his independent lab. Quite some time later, after an IACUC inspection, the new laboratory manager threw away a stack of old surgical records, which included this information. We have learned from this regrettable incident.

11. "These confirm the key principle that that the prefrontal cortex multiplexes contextual vs. socio-emotional information using orthogonal but largely orthogonal representations in both male and female mice." It is not clear to me how this statement is substantiated. All mice in Rebuttal Fig 2 are male and the authors are unable to identify the sex of the mice in each data set of the manuscript. The data are similar across mice but as the authors state, they are underpowered to detect sex differences. The authors should add a caveat to their discussion regarding potential sex differences.

Our response: The point we were trying to make was that yes, we are underpowered to detect sex differences, but because our cohort includes both male and female mice we can still assess whether the effects we see are present at the level of individual mice (because most of our figures we show the individual datapoints corresponding to single mice). If these effects are present across individual mice, it stands to reason that they are present in both male and female mice, although of course effects might be larger in one sex than the other (although we were underpowered to detect such quantitative differences). That said, we have clarified in the methods that we did not specifically examine sex-differences, and added a caveat at the end of the discussion: "while this study included both sexes, we were underpowered to detect sex-

differences in socioemotional information processing. This remains an important future direction given the strong sex biases in many neuropsychiatric conditions.”

Reviewer #2 (Remarks to the Author):

The manuscript by Frost et al. aimed to use microendoscopic calcium recording to explore whether contextual and socioemotional representations of neuronal ensembles in mPFC are segregated or intermixed and whether this cause socioemotional encoding to remap or generalize across environments. The author found that social interaction-related representations and anxiety-related representations were both largely orthogonal to contextual representations even if they were overlapped partly. Although the author provided detailed responses to the review comments, major problems proposed previously has not been resolved and the overall quality of the article is not good enough for publication.

1) The author calculated similarity based on the average value of neuronal signals to explore the similarity of neuronal ensembles in different environments. Although this method can achieve the conclusion "Neuronal ensembles representing context and social interaction overlapped more than expected by chance while nevertheless remaining largely orthogonal. Anxiety-related representations similarly generalized across environments while remaining largely orthogonal to contextual information", such similarity analysis is still too rough and not precise enough for a solid conclusion. The author should conduct more precise analysis to provide further verification.

Our Response: We measured activity patterns which underlie social interactions occurring in multiple contexts, quantified the similarity of these activity patterns, and performed statistical tests which showed that similar neuronal ensembles are repeatedly recruited during multiple rounds of social interaction occurring in different contexts. Furthermore, these same neuronal ensembles are consistently recruited by social interactions, even though this recruitment is superimposed on distinct changes in neuronal activity associated with contextual encoding. Indeed, we examined activity patterns using multiple approaches (population activity vectors, SVM classifiers, modulation indices of individual neurons), and obtained similar results in each case, consistently supporting our main conclusion.

The Reviewer seems to acknowledge that these analyses all support our conclusion. It is standard to develop metrics which quantify measures of interest, perform statistical tests to detect when these metrics change in a meaningful manner, and draw conclusions based on these statistical tests. This is what we have done, and as noted above, we have actually done this using multiple different metrics, and obtained complementary results. Perhaps there are specific questions that must be answered to support our conclusions, which have not been addressed by our multiple analyses so far, but could be addressed by specific alternative analyses. If this is the case, then we would request that the Reviewer explicitly identify both these critical questions and the specific additional analyses they would like us to perform.

2) The author claimed that "It was our goal not to define the activity of specified neuronal populations but to understand how the prefrontal circuit as a whole functions to encode different behaviors in parallel."

Our response: Our comment, "It was our goal not to define the activity of specified neuronal populations but to understand how the prefrontal circuit as a whole

functions to encode different behaviors in parallel," was made to address the concern previously raised by the Reviewer about the utility of pan-neuronal GCaMP recording. The Reviewer now raises a new set of concerns which we address one at a time below.

Ignoring the specific pattern between single neuron or ensemble and behavior and understand how the prefrontal circuit as a whole functions to encode different behaviors only by the average value of neuronal signals makes no sense.

Our response: We are actually using multiple methods to measure the relationship between single neuron and ensemble activity and behavior – specifically, how well do individual neurons (via their modulation index) encode social interaction, how well can ensemble activity be used to decode (using an SVM classifier) periods of social interaction vs. periods during which such interaction is absent, what is the distribution of neurons which significantly increase or decrease their activity during social interaction, etc. Thus, the purpose of this paper is to elucidate, not ignore, the relationship between single neuron or ensemble activity patterns and behavior.

The average value of neuronal signals can be affected by many factors such as the virus, the fiber and the surgery accuracy, etc.

Our response: There is no optical fiber here. Re: the fact that the virus and surgery influence signals, this is true for all experiments that use viral expression and fluorescent proteins.

And the average value of neuronal signals of social behavior is greatly decided by social frequency. The smaller social place leads to the higher social frequency which leads to higher average value, let along that average value can change greatly among mice.

Our response: There are several issues here. First, it is not clear exactly what the Reviewer means by '*average value of neuronal signals of social behavior,*' '*social frequency*', or '*social place*.' Perhaps '*average value of neuronal signals of social behavior'* refers to the average value of the measure of a neuron's activity during periods of social behavior? Perhaps 'social frequency' means the number of social interactions which occur, and 'social place' implies that social interactions occur at a specific location? The Reviewer seems to be implying something like the size of the social location in which social interaction occurs influences the number of social interactions, and the number of social interactions would influence the average activity level of an individual neuron during each social interaction. It is important to note that in our study, social interactions do not happen in a specific location, as both the subject mouse and the novel juvenile were freely moving. Furthermore, even if social interactions did occur in a specific 'social place' (which they do not), activity levels are computed as the fraction of time bins during which an individual neuron is active during a specific behavior, so the number of social interactions would still not simply equate to 'higher average value.'

What keeps consistent is the single curve pattern of single specific behavior and that is critical rather than just average value. Fig.3F is too rough to provide valuable information.

Our response: We are not sure what the Reviewer means by ‘the single curve pattern of single specific behavior.’ Perhaps the Reviewer is implying that there is some kind of curve that represents a mapping between behavior and neural activity? This does not correspond to the actual analysis we performed. In our analysis, frames are assigned binary labels corresponding to whether or not mice were engaged in social interaction, and we looked at differences between population activity vectors, modulation indices reflecting the activity levels of individual neurons, and/or SVM outputs during one type of frame (e.g., social) vs. another (e.g., nonsocial). Figure 3F plots the distribution of modulation indices for every individual neuron for social behavior on the y-axis and for context on the x-axis. As a result, far from being ‘rough,’ Fig. 3F actually shows the exact value of social and context modulation for every neuron.

3) The author used the word “orthogonal” for many times but what is the clear meaning of this word to the neuronal ensemble? The author achieved the conclusion “social interaction-related representations and anxiety-related representations were both largely orthogonal to contextual representations” by different algorithm analysis but what is the specific biological significance?

Our response: The Reviewer is asking what is the meaning and biological significance of the word ‘orthogonal’ in the context of neuronal ensembles. The term ‘orthogonal’ comes from geometry and linear algebra and refers to two lines or vectors which are at right angles relative to each other. Because vectors which are at right angles have a dot product of zero, and the normalized dot product a measure of correlation, it can also refer to the absence of correlation. In neuroscience, orthogonal is typically used to refer to patterns of neural activity which have a dot product of zero, i.e., when plotted as vectors in state space would be oriented at right angles relative to each other. Biologically, patterns of neural activity which are orthogonal are of great interest because they do not interfere with each other, i.e., the can be superimposed with no loss of information. Thus if two different types of information (e.g., related to social interaction and context) are encoded via orthogonal patterns of activity they can be represented simultaneously in the same population of neurons without interfering with each other. This has led to great interest in orthogonal patterns of neural representations. For example, a PubMed search for ‘orthogonal’ and ‘neural’ yields 1,991 results. Another example which shows that the topic of orthogonality between neural ensembles is highly timely, is the recent publication from Sun, Spruston et al: ‘Learning produces an orthogonalized state machine in the hippocampus’, Nature, 2025.

My understanding of this paper is that the author found social/anxiety ensemble and contextual representations have different average signal value. If so, this conclusion is meaningless.

Our response: We apologize for the misunderstanding. The Reviewer’s statement, ‘*that the author found social/anxiety ensemble and contextual representations have different average signal value’* is not an accurate description of what we found. We found that social/anxiety ensembles and contextual representations are overlapping but largely orthogonal. Critically, we are not simply computing the average value of signals across an entire neural ensemble.

During the encoding of a specific behavior such as social interaction, we observe that some neurons increase their activity, while others decrease their activity. Thus, a neuronal ensemble consists of positively and negatively modulated neurons both of which contribute to the encoding of the behavioral information. The critical point that may not have been clear is that ensembles for different behaviors (for example social behavior and contextual encoding) utilize overlapping populations of neurons. Rather that compare the level of activity averaged across a population of neurons, we are examining the orientation of these patterns of activity in a high dimensional space.

Prior to undertaking these experiments we reasoned that it was quite possible that social encoding might be accomplished in a context-dependent manner, meaning that entirely different patterns of activity might represent social interaction in different contexts. Alternatively, it was possible that social behavior and contextual information would be sparsely encoded by distinct, non-overlapping sets of neurons. We show that this is not the case, and that the PFC actually encodes social information and contextual information using highly overlapping sets of neurons, but nevertheless encodes social information in a context-invariant manner by using orthogonal representations for social interaction vs. context. Critically, when social interaction occurs in a new context, neurons which are positively modulated by social interaction in the previous context continue to be positively modulated in the second context, ands similarly neurons which are negatively modulated in one context continue to be negatively modulated in another, permitting social information to be reliably encoded despite the context-dependent reorganization of prefrontal activity.

4) What is the onset of social interaction? Fig.1 E showed that the signal recorded for 60 s but a single social behavior can not last for 60 s. The author intended to calculate the average signal value as a whole, but as what we comments previously, it would lost many information and such kind of analysis fail to give information with sufficient value.

Our Response: There seems to be a misunderstanding about Figure 1E. The x-axis in this experiment goes from 0 to 60 minutes – this represents an entire experiment, not a single period of social interaction. The black bars at the bottom indicate periods when the mice are engaged in social interaction. We often observe that single bouts of social interaction, particularly following introduction of a novel intruder can be quite prolonged, lasting 10s of seconds to over a minute, though most of the bouts are significantly shorter.

5) In Figure 4, the author concluded that "Context-invariant patterns of activity for anxiety-related information". Does that mean the average signal value of vmPFC is the same among different mazes? If so, what is the meaningfulness? The mice could all be anxious among these mazes and average signal value just should be the same.

Our response: Again, we never average the activity level across neurons. We are measuring multineuron patterns of activity. Some neurons increase their activity and other decrease their activity during specific behaviors. All of our analyses are based on examining the pattern of these increases and decreases across neurons, not any kind of value averaged across neurons.

6) How did the data in Fig.1 E transform into the data in Fig.1 F and what is the

calculation process to produce the cumulative distribution curve. What is the meaning and calculation method of the abscissa “frame active”. The authors did not elaborate this issue very well.

Our response: The term ‘frame’ refers to a single image within a series that forms a movie. As stated in the methods, we perform calcium imaging with a frame rate of 20 Hz, and compute the frames in which each neuron was active. As stated in the legend, Fig. 1F plots the “cumulative probability function for the proportion of frames that each neuron was active during social bouts.”

7) What does “frame” mean specifically? Such as “Bar graph showing the proportion of highly active neurons (>7.5% of frames)”. The word “frame” is hard to understand. Please use more precise biological phrases or neuroscience-related words or give a definition.

Our response: We apologize that this was not clear. ‘Frame’ is commonly used to describe the basic unit of movies (including Ca2+ imaging datasets). We have added a sentence to the Methods clarifying this.

8) As pointed out before, in Fig.1 D there are some blue bars representing social interaction and there are four blue bars in each epoch. Does that mean the author would make the test mice social with partner for four times in a single epoch? Furthermore, these blue bars are of inconsistent thickness. Some blue bars are obviously more thick and the distance between blue bars is different. Does that mean the social time is different? Do thick blue bar mean more social time and the bigger distance between blues bars mean longer non-social interval? If so, the author should note it in the legend. And if not, then these elements should be deleted.

Our response: As stated in the methods, each epoch consists of 5 minutes when mice are alone in their home cage followed by 5 min when they are exposed to a novel juvenile mouse. Mice are freely behaving, so they initiate and control the duration of each individual period of social interaction. As this is a cartoon, the variable widths of these bars and of the associated gaps is simply meant to convey that the exact durations and timings of these interactions are variable. We have added the following sentence to the figure legends to make this more clear: “Each epoch (brown box) consisted of 5 min during which the mouse was alone followed by 5 min during which it was exposed to a novel juvenile mouse and could engage in multiple interactions of variable duration (blue bars).”

Reviewer #3 (Remarks to the Author):

The revised version of the manuscript has been improved by the addition of a Supplementary Table showing classifier performance, updated figure labeling, and editing of the text. I have two remaining concerns.

1. The authors should interpret the significance of the high false negative rate in the performance of the classifier in the Train-Test Between Epoch condition.

Our response: We believe this is most likely related to the sparse nature of calcium activity (i.e., a lot of frames are empty or have minimal activity). We have added text stating this on page 4 of the Results.

2. My previous comment about Supp Fig. 8 was not fully addressed. I had asked whether there are significant differences between the homecage alone and the nonsocial with conspecific conditions. This is important because reviewers had concerns about pooling these periods together for analysis. If the conditions are statistically different than this should be stated and pooling the data should be justified.

Our Response: We added additional analysis in Supplementary Figure 9 which shows that periods of active social interaction/investigation can be readily discriminated from periods where the mouse is alone or periods in which the juvenile conspecific is present, but the subject mouse is not actively engaged in interaction/investigation. On the other hand, periods in which the mouse is alone are poorly differentiated from periods in which the juvenile conspecific is present but the subject mouse is not actively engaged in social interaction. We analyzed this by computing a ‘Discrimination Index’ based on the similarity between population activity vectors associated with each behavior. We believe this addresses this very reasonable question.
